# Alternative splicing and related RNA binding proteins in human health and disease

**DOI:** 10.1038/s41392-024-01734-2

**Published:** 2024-02-02

**Authors:** Yining Tao, Qi Zhang, Haoyu Wang, Xiyu Yang, Haoran Mu

**Affiliations:** 1 Department of Orthopedics, Shanghai General Hospital, Shanghai Jiao Tong University School of Medicine, 200000 Shanghai, China; 2 Shanghai Bone Tumor Institution, 200000 Shanghai, China; 3 Department of Biochemistry and Molecular Cell Biology, Shanghai Key Laboratory for Tumor Microenvironment and Inflammation, Shanghai Jiao Tong University School of Medicine, 200000 Shanghai, China

**Keywords:** Diseases, Developmental biology, Cancer, Cancer

## Abstract

Alternative splicing (AS) serves as a pivotal mechanism in transcriptional regulation, engendering transcript diversity, and modifications in protein structure and functionality. Across varying tissues, developmental stages, or under specific conditions, AS gives rise to distinct splice isoforms. This implies that these isoforms possess unique temporal and spatial roles, thereby associating AS with standard biological activities and diseases. Among these, AS-related RNA-binding proteins (RBPs) play an instrumental role in regulating alternative splicing events. Under physiological conditions, the diversity of proteins mediated by AS influences the structure, function, interaction, and localization of proteins, thereby participating in the differentiation and development of an array of tissues and organs. Under pathological conditions, alterations in AS are linked with various diseases, particularly cancer. These changes can lead to modifications in gene splicing patterns, culminating in changes or loss of protein functionality. For instance, in cancer, abnormalities in AS and RBPs may result in aberrant expression of cancer-associated genes, thereby promoting the onset and progression of tumors. AS and RBPs are also associated with numerous neurodegenerative diseases and autoimmune diseases. Consequently, the study of AS across different tissues holds significant value. This review provides a detailed account of the recent advancements in the study of alternative splicing and AS-related RNA-binding proteins in tissue development and diseases, which aids in deepening the understanding of gene expression complexity and offers new insights and methodologies for precision medicine.

## Introduction

Alternative splicing (AS) of pre-mRNA is a crucial aspect of gene regulation, significantly enriching transcriptome content and promoting diversity of both transcriptome and proteome.^1^ AS plays a pivotal role in tissue development and differentiation, and key cellular pathways of higher eukaryotes. A multitude of studies have underscored the ability of AS to allow each gene to generate multiple mRNA variants, showcasing the evolutionary advantage of higher eukaryotes. Intriguingly, AS is not exclusive to genes that encode mRNA but also extends to noncoding RNAs. These variants may or may not give rise to protein variants.^2,3^

Mechanically, AS of pre-mRNA is facilitated by the spliceosome, a significant macromolecular complex that comprises five small nuclear RNAs (U1, U2, U4, U5, and U6) and hundreds of protein combinations, collectively known as small nuclear ribonucleoproteins (snRNPs), which is assembled through the recruitment of cis-acting elements and trans-acting factors. This complex directs a series of RNA–RNA, RNA-protein, and protein–protein interactions. Moreover, splicing regulatory elements (SREs), located in the enhancer or silencer regions of gene introns and exons, are known as exon or intron splicing enhancers (ESE or ISE) or silencers (ISS or ESS), regulating AS by binding to corresponding trans-acting factors.^4^ RBPs, crucial regulators of AS, interact with RNA to form ribonucleoprotein complexes. This interaction determines the maturation and fate of their target RNA substrates and regulates various aspects of gene expression, including pre-mRNA splicing and polyadenylation, RNA stability, RNA localization, RNA editing, and translation.

Numerous RBPs participate in one or more of the physiological and pathological processes. Normal functions of RBPs are vital for human physiology, as defects in RBP function have been associated with genetic and somatic diseases such as neurodegeneration, autoimmune diseases, and cancers.^5^ A comprehensive analysis of AS in 8705 patients with 32 types of cancer in the TCGA database has revealed a significant upregulation of alternative splicing events (ASEs) in pan-cancer cells.^6^ RBPs can induce exon inclusion or exclusion or alternative use of 5′ or 3′ splice sites by binding to pre-mRNA exons (or their flanking introns).^7^ Key components of various signal transduction pathways also exhibit a multitude of ASEs that regulate biological functions such as normal cell growth, development, differentiation, migration, and apoptosis.^8^ The advent of high-throughput sequencing methods in transcriptome research has revolutionized our understanding of AS. Under both physiological normal and disease states, functionally coordinated and biologically significant ASE networks are being discovered in an increasingly diverse array of cell types.^9^ As the pathogenesis of various diseases is elucidated, numerous studies have demonstrated that abnormal AS of pre-mRNA plays a pivotal role in the onset and progression of diseases.^10,11^

In this review, we begin by summarizing the key milestones in the research history of AS of pre-mRNA. We then delve into the specific mechanisms of pre-mRNA AS, the structure and function of RBPs related to AS, and recent developments in AS and AS-related RBPs (Table 1). Subsequently, we discuss the regulatory role of AS-related RBPs under both physiological and disease states, with a particular emphasis on areas such as tumors that have attracted significant attention in recent years. Lastly, we have compiled the documented interactions of AS-related RBPs in the realm of health and disease, as elaborated in this review. In addition, we present a comprehensive summary of the advancements in targeted therapies pertaining to AS-related RBP, which includes both the drugs that have been reported (Table 2) and those that have progressed to clinical trials (Table 3).

**Table 1:** Overview of AS-related RBPs: composition, structure, binding sites, and function

RBP	Family members	Structure	Reported binding sites	Functions in cells
ESRP^460–462^	ESRP1, ESRP2	RRMs, N-terminal DNAQ-like exonuclease domain	GU-rich sequence motifs in the ISE/ISS-3	Development of cells, especially in epithelial-related genes such as FGFR2.
CPEB^463–467^	CPEB1, CPEB2, CPEB3, CPEB4	RRMs, ZnF	Cytoplasmic polyadenylation elements in the 3’UTR of mRNA	Processes of the human nervous system development, such as learning and long-term memory. Also in oogenesis and embryonic development.
ELAV/Hu^468,469^	HuA, HuB, HuC, HuD	RRMs	AU-rich elements in the 3’UTR of mRNA	RNA stability and prevention of mRNA degradation and deadenylation.
HNRNP^470^	HNRNPA, HNRNPAB, HNRNPC-U	RRMs, quasi-RRM, glycine-rich domain constituting an RGG box and a KH domain	Motif rich in uridine tracts.Typical sequences at 3’UTR and 5’UTR of mRNA, such as UAGGGA/U (HNRNPA1), UUAGGG (HNRNPA2/B1).	Stabilize nascent RNA, regulate alternative splicing (exon skipping or intron retention), transport mature RNA, and control translational repression or enhancement.
IGF2BPs(VICKZ, IMP)^471–473^	IGF2BP1, IGF2BP2, IGF2BP3	RRMs, KH domain	Recognize and selectively bind m6A-modified mRNAs	Cell growth, stem cell maintenance, and differentiation during development
ZEB^474–477^	ZEB1/2	homeodomain (HD), ZFD, coactivator binding domain (CBD), CtBP interaction domain (CID), and the p300-CBP-associated factor (P/CAF) binding domain	E-promoter DNA sequence [CACCT(G)], interaction with corepressor CtBP	Function in mammals as transcriptional repressors via cooperation with activated SMAD proteins and by recruitment of either the corepressor C-terminal binding protein (CtBP) or histone deacetylase complexes
KHSRP^478,479^	KHSRP	KH domain	G/U-rich intronic splicing enhancer element, 3’UTR AU-rich elements	mRNA decay, microRNA biogenesis and KHSRP-long Noncoding RNA Interaction in Gene Expression Control.
LARP^480–482^	LARP1, LARP3, LARP4, LARP6, LARP7	La motif (LAM), RRM	Stem-loop structures in or around the start codon of target mRNA transcripts, interaction with 5’TOPs, poly(A)-mRNAs, and RNA polymerase III transcripts	Protect nascent RNAP III transcripts from untimely 3′ exonucleolytic digestion, coordinating RNA binding and subcellular trafficking
Lin28^483^	Lin28A, Lin28B	CSD, Cys-Cys-His-Cys (CCHC) ZnF	Binds pre-let-7 at the terminal loop and the bulge GGAG motif	Germ lineage, cellular metabolism, and stem cells during the development
MSI^484^	MSI1, MSI2	RRM	Motifs variously defined as [(G/A)U(n)AGU, r(GUAG) and r(UAG), (UAG), and other poly-U motifs. ACCUUUUUAGAA’ motif and other poly-U sequences, UAG motifs, and UAG-containing motifs +/− additional flanking nucleotides	Regulating normal cell differentiation and organ development, regulating spermatogenesis and embryogenesis
PUM^485^	PUM1, PUM2	PUF domain	UGUANAUA motif	Directs mRNA degradation, stabilizes mRNA, activates translation, and/or storage in specific subcellular compartments. Control stem cell fate in many contexts
QKI^171^	QKI-5, QKI-6, QKI-7	KH domain, QUA1 domain, QUA2 domain	Recognize and bind a bipartite consensus sequence ACUAAC motif	Contribute to neural stem cell, vascular, muscle, and monocyte cell differentiation via regulating AS, RNA stability, and gene transcription.
RBM^486,487^	RBMY, RBM3, RBM5, etc. Up to now, more than 50 RBM proteins have been identified	RRMs, RBM, RNP, CSD, ZnF	Binding to the exon/intron region near the splice site of mRNA, depending on the specific RBM	Functions in mRNA transport, translation, capping, splicing and stability Splice site selection (Spermatogenesis and germ cell development)
SAM68^488^	SAM68	KH domain	Poly(U) and poly(A) RNA, and high-affinity binding sequences UAAA or UUAA	Regulation of G1/S transition during the cell cycle
SRSF^415^	SRSF1-12, TRA2A, TRA2B	Serine/arginine-rich domain at the N-terminal RRMs at the C-terminal	Exonic or intronic splicing enhancers(ESEs or ISEs) or silencers (ESSs or ISSs)	Regulate splice site recognition and spliceosome assembly Regulate RNA metabolic events, including transcription, nonsense-mediated decay mRNA export
TIA1/TIAR^489,490^	TIA1a/TIARa, TIA1b/TIARb	RRM, glutamine, and asparagine (Q/N)-rich carboxyl-terminal domain	Uridine-rich sites located in the 3′-UTR regions	Apoptosis, Apoptosis, Cell Proliferation and Cell Cycle, Mitochondrial Dynamics, Embryonic Development, associated with tissue-specific splicing events
UNR/CSDE1^491^	UNR/CSDE1	CSDs	Cis-acting instability element in the 3’UTR, 3’ A-rich region	Cell cycle, apoptosis, differentiation, and dosage compensation acting as a bridge to connect RNAs and proteins that cannot bind directly to each other

**Table 2:** Related therapeutic targets: targeting specific RBP

Drugs	Target	Therapeutics	Reference
VPC-80051	HNRNPA1	VPC-80051 directly interacts with HNRNPA1 RBD and reduces the level of AR-V7 mRNA in the 22Rv1 CRPC cell line	^492^
SPHINX31	SRPK1	Inhibits the phosphorylation of SRSF1 and promotes angiogenesis caused by VEGF-A isoforms.	^493^
Tasisulam, Chloroquinoxaline, sulfonamide, Indisulam	RBM39	Promotes RBM39 recruitment to the CUL4-DCAF15 E3 ubiquitin ligase, leading to multi-ubiquitination and proteasomal degradation of RBM39	^494^
SM09419	CLK/DYRK	Causes erroneous splicing and inactivation of the apoptosis inhibitor XIAP, downregulating the anti-apoptotic protein BCL2A1 related to venetoclax resistance	^295^
Auranofin	NONO	Regulates the abnormal ASE of GPX1, inhibiting tumor growth, invasion, and redox balance	^221^
TG003	SRSF1	Inhibits the activity of Clk1/4, leading to the dephosphorylation of SFRS1, thereby inducing the subcellular relocalization of SFRS1 and inhibiting the ASE of SFRS1-dependent pre-mRNA.	^495^
TG693	SRSFs	Inhibits the phosphorylation of SRSFs that are substrates of CLK1, and regulates pre-ribonucleic acid splicing in skeletal muscle	^496^
T025	SRSFs	Inhibits the phosphorylation of SRSFs that are substrates of CLK2, inducing exon skipping of pre-mRNA AS regulated by SRSF1	^497^
PRMT5-IN-31	PRMT5/HNRNPE1	Acts as PRMT5 inhibitor/HNRNPE1 upregulator	^498^
Manumycin-A	Ras signaling, HNRNPH1	Inhibits the Ras signaling pathway and HNRNPH1 expression to suppress the biogenesis and secretion of CRPC cell exosomes	^499^
Riluzole	HNRNPA1	Inhibits IRES-dependent translation, and blocks the binding of HNRNPA1 with cyclin D1 and MYC IRES, thereby significantly reducing the translation efficiency of these transcripts	^500^
JL014	HNRNPE1	Increases the mRNA and protein levels of HNRNP1 in HUVEC	^501^
Spinraza	HNRNPA1	Blocks the intron binding site of HNRNPA1, promotes the inclusion of exon 7 of SMN2 and the level of full-length SMN2 mRNA, treating SMA	^502^

**Table 3:** Published or ongoing Clinical Trials on RBPs related to AS

Diseases	Targets	Finding/purpose	Reference
Myelodysplastic syndromes (MDS)	SF3B1^MUT^	In SF3B1^MUT^ MDS patients, the alternative transcript of FAM132B/ERFE^+^12 is translated into ERFEVPFQ protein, and together with the standard transcript, leads to overexpression of ERFE. The plasma hepatic phospholipid concentration in SF3B1MUT MDS is similar to that in healthy non-donor controls, and the prognosis is better than SF3B1WT, but the excessive iron load in cardiac and hepatic tissues when transfusion-dependent can affect the expected lifespan. Taking erythropoietin agonists or targeting overexpression of ERFE may provide potential strategies for SF3B1MUT MDS patients to prevent iron overload and improve erythrocyte production	^503^
Diffuse large B-cell lymphoma (DLBCL)	Genome-wide gene and exon expression profiles	AS plays an important role in the pathogenesis of DLBCL. ASEs may affect drug resistance by regulating the function and activity of ATP-binding cassette transporters. The alternative exon usage of the APH1A gene has an impact on the prognosis of DLBCL and has therapeutic significance. Exon 2 skipping in the promoter region of the ABCB1 gene is an adverse event related to lymphoma progression.	^504^
Chronic lymphocytic leukaemia (CLL)	SET mRNA isoforms	CLL patients with relatively higher expression levels of SETA isoform mRNA (high SETA/B mRNA ratio) have significantly shorter TTFT and OS. Moreover, this ratio can identify patients with poorer clinical prognosis in the previously defined high-risk CLL population	^505^
Systemic lupus erythematosus (SLE)	SRSF1	SRSF1 can regulate the AS of CD3ζ 3’-UTR, enhancing the expression of CD3ζ in human T cells. The average expression of Srsf1 mRNA in T cells of patients with ALE is lower, and the expression of RSF1 protein is reduced. The expression is even less in patients with severe conditions, and this change is mainly affected by SRSF1 ubiquitination.	^506^
Spinal muscular atrophy (SMA)	SMN	The SMN splicing modifier RG7800 has good tolerance at test dose levels. PD data from healthy adults and SMA patients have demonstrated that oral SMN splicing modifiers can upregulate SMN2 FL mRNA and increase systemic SMN protein levels, potentially becoming the first oral treatment for SMA	^507^
Myeloproliferative neoplasms (MPNs)	HNRNPH1 HNRNPK	Evaluate the expression patterns of HNRNPH1 and HNRNPK genes in myeloproliferative tumors as potential indicators of disease progression and potential therapeutic targets	NCT05782985
Neurodevelopmental disorders	HNRNPs	Analyze the patterns of individuals with HNNRPs gene mutations, including their neurological comorbidities, other medical issues, and any treatments	NCT03492060
Prostate cancer (PCa)	Alternative splicing events	Analyze PCa biopsy tissue data from African and Caucasian patients to explore AS as a novel molecular mechanism for the more aggressive PCa observed in African me	NCT03424213
Type 2 diabetes	HuR	The impact of metformin on alternative gene splicing (which depends on the HuR protein) in patients with type 2 diabetes, including genes encoding insulin receptors	NCT01349387
Frontotemporal dementias (FTD)	RNA splicing alterations	Researching gene expression and RNA splicing changes in the lymphocytes of patients and “high-risk groups” will utilize RNA sequencing to identify peripheral biomarkers for disease onset and progression	NCT04014673
Adult forms of myotonic dystrophies type 1	Disrupted AS of insulin receptor and Tau protein	Cognitive impairment in patients with type 2 diabetes can be explained by the acceleration of brain lesions (especially Tau protein lesions and brain atrophy)	NCT04656210
AML, MDS, CMML	Mutations in Splicing Factor Genes	Phase II Clinical Trial of E7820 in the Treatment of Recurrent/Refractory Myeloid Malignancies with Splicing Factor Gene Mutations	NCT05024994
AML, MDS, CMML	SRSF2- and SF3B1-mutation	Evaluating the Safety, Pharmacokinetics, and Pharmacodynamics of Splicing Regulator H3B-8800 (RVT-2001) in Subjects with Myelodysplastic Syndromes, Acute Myeloid Leukemia, and Chronic Myelomonocytic Leukemia	NCT02841540^508^
Huntington’s disease, HD	Pre-mRNA - U1 snRNP complex	Branaplam Study in Adult Patients with Huntington’s Disease (HD), Aiming to Determine the Correct Dosage Required to Reduce Mutant Huntingtin Protein (mHTT) Levels in Cerebrospinal Fluid (CSF) to Achieve Long-Term Efficacy”	NCT05111249^509^
Spinal muscular atrophy (SMA)	Survival motor neuron (SMN)	Investigating the Safety, Tolerability, and Pharmacokinetics/Pharmacodynamics of Risdiplam (RO7034067) in Adult and Pediatric Patients with Spinal Muscular Atrophy	NCT03032172^510^
Spinal muscular atrophy (SMA)	Survival motor neuron (SMN)	Investigating the potential value of SMN circRNAs as biomarkers of SMA, in terms of prediction of disease severity and response to treatments	NCT05760209
AML and HR-MDS	Cdc2-like kinase, SRSF2	Evaluating the Safety, Tolerability, Pharmacokinetics, and Pharmacodynamics Characteristics of CTX-712 in Patients with Recurrent/Refractory (R/R) Acute Myeloid Leukemia (AML) and High-Risk Myelodysplastic Syndromes (HR-MDS)	NCT05732103^511^

## Evolution of research developments on the alternative splicing and AS-related RNA-binding proteins

**Fig. 1:**
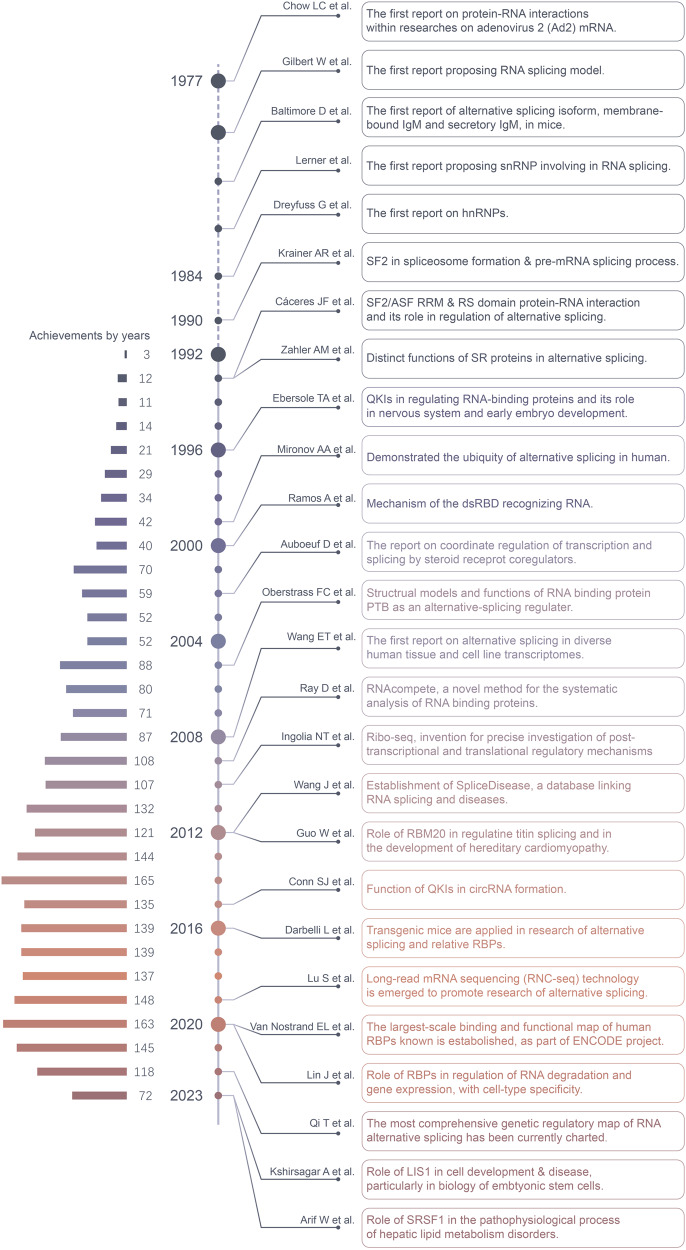
The historical timeline on milestones in RBPs related to alternative splicing. The study of AS and AS-related RBPs can be traced back to the 1970s. In 1977, the interaction between RBPs and mRNA was first reported. The following year, the mechanism of AS was proposed, which marked the official debut of AS and AS-related RBPs in the scientific community. Early research focused mainly on two types of RBPs: SR proteins and HNRNP proteins, which recognize different splicing sites and thus affect splicing choices. In recent years, with the development of technology, more and more RBPs have been identified, such as ESRPs, SRSFs, and HNRNPs, which play important roles in the process of alternative splicing. With the development of sequencing technology, the functional map of human RBPs and the genetic regulatory map of RNA alternative splicing were published in 2020 and 2022, respectively, providing new directions for studying the mechanisms of complex human diseases. This figure was drawn by Adobe Illustrator

The evolution of research developments of AS and RBPs is a complex and continuously evolving field (Fig. 1). In the 1970s, scientists first observed the phenomenon of AS, a significant discovery as it revealed that a single gene could encode multiple proteins. As our understanding of RNA biology deepened, scientists began to study RBPs, proteins that can bind with RNA and influence their stability, transport, translation, and splicing. In the early 21st century, researchers began to discover that RBPs play a key role in many diseases, including neurodegenerative diseases and cancers. With the development of high-throughput sequencing technology, scientists have been able to study AS and RBPs at the whole-genome level, greatly enhancing our understanding of these two fields. The advent of CRISPR/Cas9 technology has allowed scientists to precisely edit genes, including those that encode RBPs. This provides a powerful tool for studying how RBPs influence AS. The field of AS and RBPs continues to evolve and deepen.

Rapid advancements are currently being made in the field of AS-related RBPs:

1. RBPs exert control over ASEs, by identifying and adhering to distinct RNA sequences and by recognizing unique secondary or tertiary structures in RNA, which in turn influences the assembly of the spliceosome complex and its interaction with pre-mRNA (refer to Table 3).
2. RBPs are integral to the normal developmental processes within the human body, including critical functions such as cell differentiation, lineage determination, acquisition, and maintenance during tissue identity and organ development, which will be discussed subsequently focusing on various systems.
3. RBPs play a pivotal role in the progression of a diverse range of diseases, including both oncological and non-oncological diseases. The research is centered on the aberrant ASEs and the subsequent alterations in downstream pathways. Highly heterogeneous tumors, including glioblastoma, breast cancer, lung cancer, and liver cancer, have been the subject of extensive investigation. Moreover, neurodegenerative diseases have recently become a significant point of contention, with a primary focus on the regulatory mechanisms of PTBP1 in the context of neuronal development and differentiation. These topics will be discussed in greater detail in the subsequent sections of the text.
4. Cutting-edge technologies, designed for the study of AS and associated RBPs, are capable of visualizing and pinpointing ASEs at both cellular and subcellular levels. This advancement provides new pathways for the identification of RBPs that play a role in regulation.
5. Advanced molecular mechanisms govern the regulatory role of AS and AS-related RBP in both health and disease. These mechanisms encompass a comprehensive understanding of poison exons, the impact of methylation modifications mediated by RBPs, and the distinctive mutations associated with AS. These topics will be elaborated upon in the discussion section.

## Overview of AS mechanisms and RBP families: components and working models

### Alternative splicing: cis-acting elements

Cis-acting elements are concise nucleotide sequences situated in pre-mRNA exons and introns, functioning as binding sites for trans-acting elements, and guiding the assembly of spliceosomes and the recruitment of SFs. The 5’ and 3’ splice sites are consensus motifs positioned at the intron/exon boundaries, while the polypyrimidine tract and branch point adenine reside within the intron. These sequences can be recognized by spliceosome components, thereby catalyzing AS. SREs are located near splice sites and recruit SFs to regulate the assembly of spliceosomes either negatively or positively. This facilitates the inclusion/exclusion of specific exons or the use of alternative splice sites. SREs are categorized into four types: exon splicing enhancers (ESEs), exon splicing silencers (ESSs), intron splicing enhancers (ISEs), and intron splicing silencers (ISSs).^12,13^ The third phase of the Encyclopedia of DNA Elements (ENCODE) project has introduced a new dataset of RNA elements recognized by RBPs in the human genome. This has broadened the catalog of functional elements encoded by the human genome, adding a significant number of elements that function at the RNA level through interactions with RBPs.^14^

### Alternative splicing: RNA-binding proteins (trans-acting elements/splicing factors)

Trans-acting elements, primarily composed of RBPs known as SFs, play a pivotal role in AS. RBPs govern various facets of cellular RNA, including production, maturation, localization, translation, and degradation.^15^ Many RBPs contain defined RNA-binding domains (RBDs) that bind to RNA in a sequence and/or structure-specific manner. The human genome encodes at least 1500 RBPs with defined RBDs.^16^ Two major classes of RBPs are commonly recognized based on their ability to enhance or inhibit exon inclusion: SRSFs and HNRNPs. ESEs and ISEs predominantly recruit SRSFs, acting as splicing activators, while ESSs and ISSs are typically recognized by HNRNPs and act as splicing inhibitors. SRSFs and HNRNPs often act competitively when selecting AS sites and exons.^17^ Moreover, genes encoding SRSFs and HNRNPs undergo AS and initiate nonsense-mediated mRNA decay (NMD), forming a negative feedback loop that introduces an autoregulation process to cellular ASEs. The normal function of SRSF family members depends on the phosphorylation regulation of Cdc2 kinase (CLKs) and SR-specific protein kinase (SRPKs). Structurally, AS-related RBPs commonly contain RBDs including RNA recognition motif (RRM), K homology domain (KH), double-stranded RBD (dsRBD), cold shock domain (CSD), arginine–glycine–glycine domain (RGG), tyrosine-rich domain as well as CCHC, CCCH, ZZ-type zinc finger (ZnF). Given the diverse functions in cells, RBPs can be divided into epithelial splicing regulatory proteins (ESRP1), cytoplasmic polyadenylation element binding protein family (CPEB1/2), Hu-antigen R (HuR), heterogeneous nuclear ribonucleoprotein family members (HNRNPA/D/H/K/M/E/L), insulin-like growth factor-2 mRNA family members (IMP1/2/3), zfh family of transcription factors (ZEB1/2), KH-type splicing regulatory protein (KHSRP), La ribonucleoprotein domain family members (LARP1/6/7), Lin28 homolog proteins (Lin28), Musashi protein family (MSI1/2), Pumilio protein family (PUM1/2), Quaking (QKI), RNA-binding motif protein family (4/10/38/47), Src-associated substrate during mitosis of 68 kDa (SAM68), serine and arginine-rich splicing factor (SRSF1/3), T-cell intracellular antigens (TIA1/TIAR), and Upstream of N-Ras (UNR)^18^ (refer to Table 1).

### Mechanism of alternative splicing

**Fig. 2:**
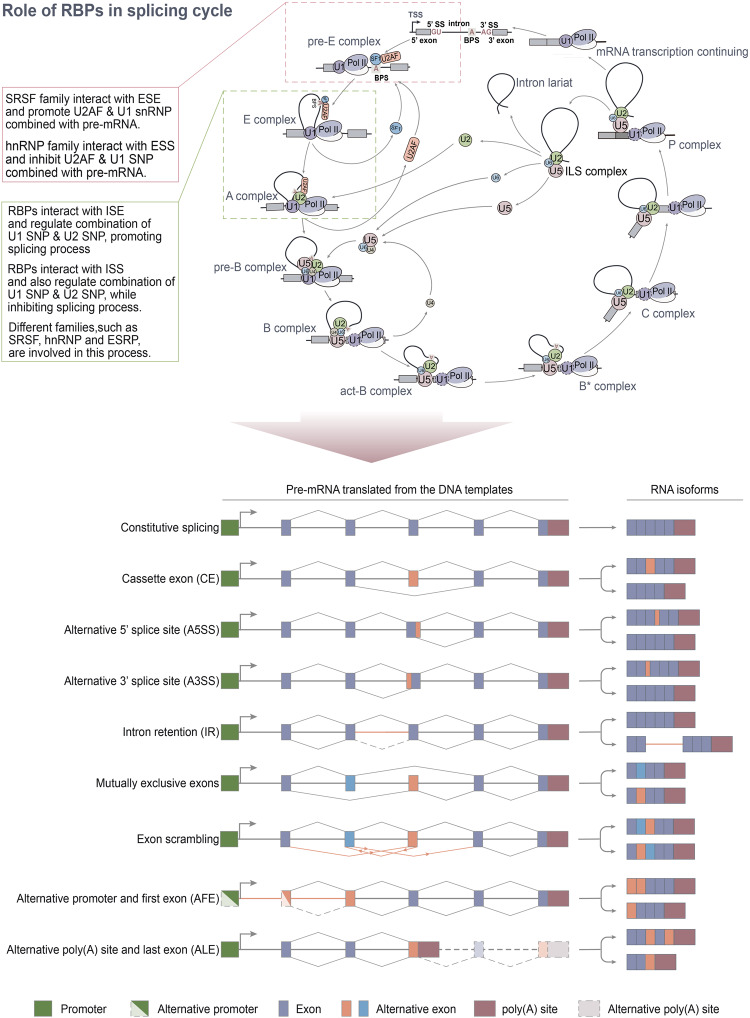
The splicing cycle, RBPs and final types of alternative splicing. The upper figure shows the detailed RNA splicing process. U1 snRNP, SF1, and U2AF recognize and bind to 5’ss, 3’ss, and branch point, respectively, forming the pre-E complex and E complex after conformational changes. U2 snRNP is recruited and displaces SF1, forming the A complex. U2AF leaves from the complex, and U4, U5, and U6 snRNP are recruited, forming the pre-B complex. The B complex is formed after U1 leaves. Then, the intron is spliced, and exons are ligated via two-step transesterifications. During the formation of the pre-E complex, members of the SRSF family typically interact with ESEs and facilitate the binding of U2AF and U1 snRNP to pre-mRNA. Conversely, members of the HNRNP family usually interact with ESSs and impede the binding of U2AF and U1 snRNP to pre-mRNA. Other AS-related RBPs, such as ESRPs, SRSFs, and HNRNPs, have been demonstrated to play a crucial role in the conformational changes of the E and A complexes. The bottom figure shows the identified alternative splicing (AS) in mammals, which mostly results in the binding of RNA polymerase II to RNA and regulation of exon identification. This figure was drawn by Adobe Illustrator

The spliceosome, recruited by cis-acting elements and trans-acting factors, regulates both constitutive splicing and ASEs. This substantial macromolecular complex comprises five small nuclear RNAs (U1, U2, U4, U5, and U6) and hundreds of protein combinations known as snRNPs. The complex AS regulation process is executed step by step through the dynamic assembly of snRNPs (Fig. 2).^19^ U1 snRNP binds to the 5′-ss GU dinucleotide, while SF1 and U2AF65 bind to the branch point site (BPS) and polypyrimidine tract (PPT), respectively, forming complex E. Subsequently, U2 snRNP interacts with BPS through base pairing, replacing SF1 to form complex A. This recruits U4/U6/U5-3-snRNP, with U5 snRNP binding to 3′-ss and U6 snRNP binding to U2 snRNP, forming complex B. Concurrently, U1 and U4 snRNPs are released, leading to the formation of complex C. Following two esterification steps, the intron folds into a lariat shape, and the 5′-ss is cleaved. Finally, the two exons are connected, and the lariat is released.^20^ Not all RBPs involved in regulating ASE bind to target pre-RNA with an open RRM structural domain. For instance, the C-terminal tyrosine-rich domain of RBFOX1 can promote aggregation, nucleolar localization, and splicing activation.^21^ Research has indicated that mutations in AS-related RBPs disrupt the expression ratio of small nuclear RNAs and the assembly of spliceosomes, leading to premature pathogenic termination of mRNA translation.^22^

AS can generate mRNA with different untranslated regions (UTRs) or coding sequences through several mechanisms^23^ such as constitutive AS, cassette exon (CE), intron retention (IR), mutually exclusive exon (MXE), selection use of alternative 5′ or 3′ splice site (A5SS/A3SS) and alternative first or last exon (AFE/ALE).^4,24^ These differences may affect mRNA stability, localization, or translation.^25,26^ It is important to note that there is a complex regulatory network among RBPs in the process of regulating ASE, and such interactions have not been elucidated clearly. Not all ASEs produce functional proteins. Firstly, transcripts may be noncoding and therefore will not be translated into proteins; secondly, RNA stability may be affected; thirdly, changes in mRNA localization may hinder the correct function of transcripts and/or proteins.^27–29^ AS is also influenced by epigenetic markers. Histone modifications and DNA methylation can impact exon usage by controlling the elongation speed of RNA polymerase II (RNA pol II), thereby influencing splice site selection. Additionally, these modifications may affect the recruitment of SFs to chromatin through adapters such as CHD1. Furthermore, it has been discovered that chromatin modifications can regulate the activity of alternative or latent transcription start sites (TSSs) within the genome.^30,31^

### AS-related RBPs demonstrate complex interactions modulating ASEs

RBPs independent of the spliceosome primarily regulate ASEs in a concentration-dependent manner. Numerous studies have underscored that maintaining a certain concentration of these RBPs is crucial for environmental stability.^32^ The regulatory crosstalk among major RBPs seems to be vital in maintaining such a stable status. The expression, turnover, and translation of regulatory RBPs (including AUF1, HuR, KSRP, NF90, TIA1, and TIAR) are at least partially regulated by complex interaction circuits of self-regulation and cross-regulation.^33^ However, the regulation of such regulation is not yet clear. Here is the overview of HNRNPs, SRSFs, and other AS-related RBPs.

SRSF1 can balance AS activity according to changes in total substrate load, performing such autoregulation at the single-cell level.^34^ Since most AS occurs co-transcriptionally,^35^ negative feedback autoregulation should produce feedback on local SRSF1 concentrations in the subnuclear neighborhood rather than on global average concentrations in the entire nucleus. Recent structural studies of spliceosome complexes have provided unprecedented insights into the organizational structure of this RNP machine and illustrated the diversity of splicing regulatory mechanisms.^36,37^ At the same time, genome-wide studies on RBP interactions and functions show that the complexity of physical RNA-protein and protein–protein interaction networks is as dense as the regulatory networks composed of these proteins.^1^ The process of AS is regulated by over a thousand RBPs, with RBFOX2 being one of the most extensively studied.^38,39^ RBFOX2 is widely expressed in human tissues, promoting different ASEs, thereby suggesting that other factors influence its regulatory role on AS. RBFOX2 has a central RRM that recognizes the consensus sequence (U)GCAUG, typically found in introns flanking target exons.^40,41^ When RBFOX2 proteins bind upstream of alternative exons, it promotes exon skipping, but when binding downstream of the exon, PBFOX2 produces an inclusion effect.^42,43^ RBFOX2 is part of a large assembly of splicing regulators (LASR), a multimeric complex containing HNRNPM, HNRNPH, HNRNPC, Matrin3, NF110/NFAR-2, NF45, and DDX5.^44^ HNRNPM promotes RBFOX2 to interact indirectly with RNA to regulate AS, prompting RBFOX2 to bind to non-(U)GCAUG sites on pre-mRNA.^44^ Zhou et al.^45^ pointed out that the selection of AS binding sites by RBFOX2 and the regulation of AS results are also regulated by HNRNPC and SRSF1. Of all ASEs affected by RBFOX2 deletion, 64% are directly bound to RBFOX2, and 51% are bound to pre-mRNA through typical RBFOX2 motifs, indicating that RBFOX2 uses various configurations of protein partners to recognize RNA with different patterns and binding sites. Depending on the composition of the binding complex and the characteristics of the main binding site, RBFOX2, and protein partners may target genes with different functions.

In addition to members of the RBFOX family, other AS-related RBPs have been reported to constitute an AS network. Proteins containing DZF modules play significant roles throughout gene expression from transcription to translation. ILF2, ILF3, and ZFR, three DZF proteins, are widely expressed in mammalian tissues and form mutually exclusive ILF2-ILF3 and ILF2-ZFR heterodimers.^46,47^ ZFR preferentially binds to dsRNA in vitro and is enriched on introns containing conserved dsRNA components in cells. Deletion of any one of the three DZF proteins results in similar changes in ASEs. DZF proteins also control the fidelity and regulation of dozens of highly validated mutually exclusive ASEs. DZF proteins form a complex regulatory network using ILF3 and ZFR dsRNA binding to regulate splicing regulation and fidelity.^48^ Abnormal self-regulation of AS-related RBPs promotes tumors. In oral squamous cell carcinoma (OSCC) cells, PTBP1 and PTBP2 bind to an ESS motif in exon 4 of SRSF3 and inhibit its inclusion, leading to overexpression of full-length functional SRSF3. Overexpression of SRSF3 in turn promotes PTBP2 expression.^49^ However, not all interactions between AS-related RBPs are promotive, and there are antagonistic ones. Hu et al.^50^ found that A-kinase anchoring protein (AKAP8) inhibits the splicing activity of HNRNPM that promotes EMT through protein–protein interaction, and targets CLSTN1 to cause AS isoform conversion thereby promoting EMT.

To summarize, the spliceosome, a complex macromolecular structure composed of species-specific snRNP and a multitude of RBPs, orchestrates the intricate process of AS in pre-mRNA through a series of complex interactions. Current research has unveiled interactions between non-RRM structural domains and pre-mRNA, which play a pivotal role in regulating ASEs. This introduces new avenues for future research to focus on novel structural domains in AS-RBPs and investigating the functionalities of already reported domains. Furthermore, it has been observed that the binding of RBPs at varying positions on pre-mRNA can induce a positional effect, leading to diverse ASEs. Future research endeavors could explore this positional effect of RBPs in diseases and devise corrective measures, potentially paving the way for innovative therapeutic strategies.^51^

### Summary of methods for studying alternative splicing and related RNA-binding proteins

Significant advancements have been made in systematically analyzing RBPs and their related regulatory mechanisms.^52^ This has been achieved through the application of in vitro binding, in vivo cross-linking and immunoprecipitation (CLIP) methods,^14,53^ proteomics,^54,55^ functional genomics,^56,57^ and increasingly powerful computational methods.^58^

Current research methods for RBPs primarily encompass homopolymer binding, ultraviolet cross-linking, SELEX, EMSA, genome-wide in vivo immunoprecipitation, and protein affinity purification.^59^ In addition, there is an online database (RBPDB), which includes 1171 known RBPs that users can browse by field and species. The TCGA database can be utilized to download RNA high-throughput sequencing and clinical pathological data to determine the abnormal expression of RBPs in cancers and normal issues. A CRISPR-based RNA proximity proteomics (CBRPP) method has recently been developed, which can be applied to identify proteins associated with endogenous RNA of interest in native cellular environments without pre-editing, cross-linking, or in vitro manipulation of RNA–protein complexes. CBRPP is based on the fusion of dCas13 and proximity labeling (PBL) enzymes. dCas13 can deliver PBL enzymes to target RNAs with high specificity, and PBL enzymes label proteins around the target RNA, which are then identified by mass spectrometry.^60^

Transcriptomic data utilized for AS detection primarily originate from three sources: expression sequence tags (EST), splice junction microarrays, and RNA-seq. However, there is currently a lack of universally recognized standardized AS detection methods. Each of these tools has its advantages and limitations, and when applied to the same dataset, they yield different output results.^61^ Compared to RNA-seq, single-cell RNA-seq (scRNA-seq) methods can analyze splicing heterogeneity between individual cells at a higher resolution and reveal high variability within tissues and between individuals.^62^ However, AS analysis by scRNA-seq has a strong 3′ bias, which poses a significant challenge for AS detection.^62,63^ Recently published computational methods employ large-scale RNA-seq and genotype datasets (such as the Illumina Human Body Map 2.0 project, GWAS, and GTEx) as “training” sources, predicting changes in AS between tissues and link gene mutations to specific AS patterns in health and disease.^64^ A deep neural network has been developed capable of accurately predicting splice junctions from any pre-mRNA sequence and identifying cryptic splice variants caused by noncoding mutations. These variants are deleterious in humans and are significantly enriched in patients with autism and intellectual disability.^65^ Besides, a model has recently been designed to predict how combinations of exon mutations cooperate to affect the exon 6 inclusion in mature mRNA in FAS and lead to phenotypic changes,^66^ which is based on deep learning that integrates sequence, conserved domains, and expression data into a unified predictive model.^67^

High-throughput, transcriptome-wide methods for the discovery of RNA-protein interactions are rapidly advancing. These include enhanced interactome capture (eRIC), chemistry-assisted interactome capture (CARIC), and total RNA-associated protein purification (TRAPP). These methods are complemented by high-throughput techniques that identify RNA-binding sites on RBPs (RBDmap) and RBP-binding sites on RNAs (CLIP-seq).^68^ Looking ahead, research on AS-related RBPs may trend toward the following developments, the development of new technologies and an intensified exploration into the impact of DNA methylation and histone modifications on AS-RBPs, with these findings being corroborated in disease contexts. Ye et al.^51^ recently pioneered the development of Capture RIC-seq (CRIC-seq) technology, which facilitates high-throughput analysis of specific RBP-mediated in-situ RNA–RNA interaction sites and has been successfully employed to construct a spatial interaction map of proteins such as PTBP1, HNRNPA1, and SRSF1 in HeLa cells. This groundbreaking research has shed light on the mechanism by which RBPs modulate ASEs through positional effects, mediated by alterations in RNA spatial conformation. In addition, Qi et al.^69^ have pioneered the development of a technique known as Testing for Heterogeneity between Isoform-eQTL Effects (THISTLE), which enables the efficient pinpointing of genetic regulatory sites associated with RNA AS, culminating in the creation of the most comprehensive genetic regulatory map for AS to date.

## Physiological functions of AS-related RBPs in tissue development

**Fig. 3:**
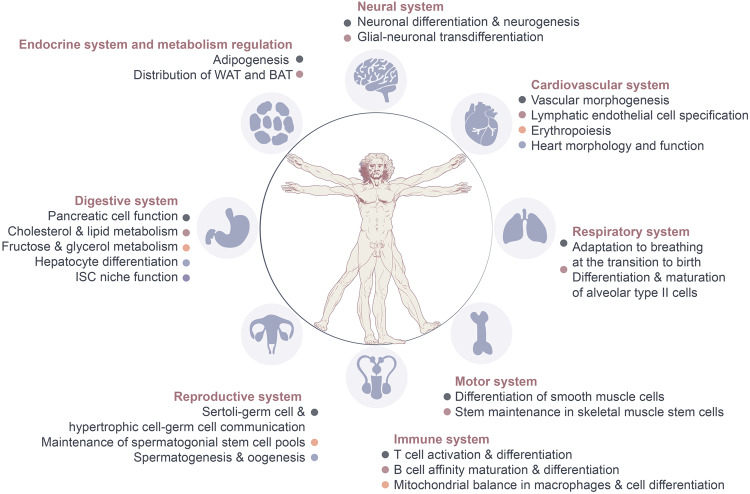
Overview of roles of related RBPs on the physiological regulation of AS in tissue development. Under normal physiological conditions, AS and RBPs enhance protein diversity by generating a multitude of protein isoforms, thereby bolstering the diversity and complexity of cellular functions. In neural system, these factors govern processes such as neuronal differentiation and neurogenesis. Within the cardiovascular system, they participate in the regulation of vascular formation, hematopoietic cell development and differentiation, and the maintenance of cardiac physiological structure. In the motor system, they primarily modulate the differentiation and stemness of skeletal muscle and smooth muscle stem cells. Within the immune system, they contribute to processes such as immune cell differentiation, maturation, and activation. In the reproductive system, they chiefly regulate cellular communication between germ cells, Sertoli cells, and hypertrophic cells, influencing spermatogenesis and oogenesis. Within the digestive system, they control liver metabolic function, pancreatic islet cell function, and the maintenance of the intestinal stem cell niche. Lastly, within the endocrine and metabolic systems, they predominantly impact fat formation and distribution. This figure was drawn by Adobe Illustrator

AS elucidates the process by which a single gene can produce multiple mature transcripts, thereby enhancing the complexity of the proteome. Under physiological conditions, numerous ASEs occur, and the transition of AS isoforms aids in acquiring the functions and characteristics of adult tissues. Coordinated alterations in a single AS are established during development to form an AS network. Recent advancements have improved our understanding of the mechanisms that coordinate AS networks and their roles in cell differentiation, organ development, and tissue homeostasis. Over the past decade, the targets of AS-related RBPs have been identified by applying high-throughput methods and transgenic animals (inducing or depleting RBPs in specific tissues). The primary aim of these studies is to identify the AS targets and binding sites of individual RBPs during development, describe the functions of RBPs in splicing coordination, and then infer the potential roles of these splicing networks in tissue and organ development.^70–72^ In this part, we provide a summary of current knowledge on tissue development regulated by AS-related RBPs (Fig. 3).

### Alternative splicing and related RBPs in the reproductive system

The testis is one of the tissues with the most AS mRNA variants, specifically manifested as a large number of exon skipping.^73^ Among these, Sertoli cells are key in creating a microenvironment to produce functional sperm. Communication between Sertoli–Sertoli cells and Sertoli–germ cells forms the ectoplasmic specialization and blood–testis barrier (BTB), which protects germ cells from immune attack and provides nutrients for germ cells.^74^ During spermatogenesis, splicing factors and AS are regulated at specific stages.^75^ With the application of gene-modified mice, novel AS-related RBPs involved in spermatogenesis have been continuously discovered, including SAM68, PTBP2, and RBM5.^76^ Notably, PTBP2 controls functional networks involved in cell adhesion and polarity and is crucial for Sertoli–germ cell communication.^77^ PTBP2 directly binds to AS targets to inhibit ASEs of multiple genes and controls ASEs that occur between mitotic and meiotic germ cells. PTBP2 also regulates the communication network between germ cells and hypertrophic cells by stabilizing the actin cytoskeleton in Sertoli cells.^77^ In addition, HNRNPH1, highly expressed in meiotic cells, is essential for ASE to regulate spermatogenesis. When HNRNPH1 is knocked out in male germ cells, abnormalities in ASEs affect meiosis and communication between germ cells and hypertrophic cells, ultimately leading to male infertility. HNRNPH1 directly binds to SPO11 mRNA and recruits PTBP2 and SRSF3 to cooperatively regulate the AS of target genes. The SPO11 gene encodes two primary isoforms (SPO11α and β), which differ due to exon 2 skipping (α) or inclusion (β). Early meiotic spermatocytes predominantly produce SPO11β, while the function of SPO11α is crucial in the late stage of meiosis. Interestingly, HNRNPH1 is also necessary for oogenesis. Deletion of HNRNPH1 in embryonic female germ cells leads to female infertility, with defects observed in meiosis and cell-cell connections.^78^ Recent studies on spermatogenesis in mice revealed that Bud31, an important component of AS, is crucial for the maintenance of spermatogonia stem cell pools and the initiation of spermatogenesis. Gene knockout leads to intron 1 retention of Cdk2, leading to reduced expression and resulting in loss of spermatogonia and male infertility.^79^ Members of the SRSF family are also involved in the regulation of spermatogenesis. In the absence of SRSF10, differentiation and initiation of meiosis fail in spermatogonia stem cells. The absence of SRSF10 interferes with ASEs in genes related to germ cell development, cell cycle, and chromosome separation, including Nasp, Bclaf1, Rif1, Dazl, Kit, Ret, and Sycp1.^80^ AS-related RBPs play a crucial role in spermatogenesis. Beyond the RBPs previously mentioned, RBM5, BCAS2, NANOS2, and DDX5 have been identified as indispensable SFs within spermatogenesis. These proteins regulate the ASEs of mRNA, which are integral to the production of sperm.^81–83^ Further exploration is needed for research on AS as well as different RBPs involved in its regulatory network.^75,84^ Recently, the continuous revelation of ASEs of noncoding RNAs has led to an increased focus on the role of circRNA in the self-renewal and differentiation of spermatogonia stem cells, as well as its impact on sperm motility.^85,86^ However, reports on the relationship between AS involved in circRNAs and spermatogenesis remain scarce. Future research endeavors should aim to delve deeper into this direction.

### Alternative splicing and related RBPs in neural system

The adult cerebral cortex and embryos show nearly 400 different ASEs. Among the genes found to be differently regulated by AS during development, 31% of genes did not alter their expression levels, indicating the involvement of ASE. Owing to the high expression of RBPs, RNA regulation in the brain is significant.^87^ This is significant as it highlights the role of ASE in gene regulation, independent of changes in overall gene expression. During brain development, a network composed of various AS-related RBPs is involved. There are dynamic changes in the expression levels of RBPs during neural development, further emphasizing the importance of these proteins in brain development.

In neural progenitor cells (NPCs), PTBP1 inhibits exon 10 inclusion of PTBP2, leading to exon skipping and transcripts with premature termination codons (PTC), as well as NMD.^88,89^ As NPCs gradually differentiate into neurons, PTBP1 is downregulated while SRRM4, which acts as a positive regulator for ASE in PTBP2, is upregulated. PTBP2, which is expressed in NPCs, is responsible for inhibiting adult-specific alternative exons in genes encoding proteins that control cell fate, proliferation, and the actin cytoskeleton. This contributes to neuronal development and tissue maintenance during tissue development.^90^ RBFOX1, another pivotal AS-related RBP in regulating ASEs during neural development, mediates the AS of exon 19 of RBFOX1 pre-mRNA, producing either nuclear (exon 19 excluded) or cytoplasmic (exon 19 included) protein isoforms. In RBFOX1 knockout neurons, more than 500 abnormal cassette-type exon ASEs occur on pre-mRNA, resulting in significant changes in exon inclusion or skipping. Further studies have discovered that PTBP1 and RBFOX1 play antagonistic roles in ASE. In NPCs, PTBP1 promotes the skipping of a toxic exon (exon-N) in filamin A transcripts to maintain NPC stratification. The inclusion of exon-N introduces a PTC, leading to protein truncation and/or NMD. Furthermore, Ninein, an important protein regulating the development of neuronal axons and the formation of centrosome structures in neural stem cells, transforms its pre-mRNA.^91^ Exon 18 of Ninein is excluded regulated by QKI-5 and, exon 29a is included mediated by RBFOX, causing its centrosome non-neuronal isoform to transform into a noncentrosome neuronal isoform related to microtubules. This induces NPCs to differentiate into neuronal cells.^92^ oreover, other RBPs are involved in neural differentiation. The neurotumor ventral antigen 2 (NOVA2) controls the exons 7b and 7c exclusion in disabled homolog 1 (DAB1), participating in microtubule signal transmission during mammalian cerebral cortex development. RBFOX3 promotes an alternative exon skipping in the signal adapter protein numb by binding to upstream intron UGCAUG elements. When RBFOX3 expression is inhibited in developing chicken spinal cord, this exon of NUMB is included, hindering neuronal differentiation.^93–96^

HuD and SAM68 play integral roles at various stages of neural development, suggesting that specific regulatory partnerships are manipulated during distinct phases of neural development.^97^ These studies provide new insights into how RBPs influence neural development through AS. Recently, the field of glial-neuronal trans-differentiation has seen significant debate surrounding PTBP1. Qian et al.^98^ have indicated that the downregulation of PTBP1 in astrocytes can stimulate the production of new functional dopamine neurons, facilitate the reconstruction of damaged neural circuits, and restore dopamine levels in the striatum in a Parkinson’s mouse model. However, subsequent studies have countered this by asserting that the knockout or downregulation of PTBP1 does not induce glial cells to differentiate into neurons.^99,100^ As we look to the future, further research is needed to delve deeper into the true role of PTBP1 in glial-neuronal trans-differentiation and to determine whether other factors may also play a part in this process.^51^

### Alternative splicing and related RBPs in the digestive system

#### Pancreas

NOVA1 plays a significant role in regulating over 5000 ASEs in pancreatic cells, primarily involving secretion, apoptosis, insulin receptor signal transduction, splicing, and transcription. In both rodent and human β cells, silencing NOVA1 inhibits insulin secretion and induces cell apoptosis after cytokine treatment.^101^ The absence of RBM4 in mice induces metabolic changes and erroneous ASE required for pancreatic cell differentiation and function. Specifically, RBM4 can regulate the AS of transcription factors ISL1 and PAX4, thereby regulating the expression of the insulin gene.^102^ This highlights the intricate role of RBPs in maintaining pancreatic cell function and overall metabolic health.

#### Liver

During liver development and maturation, a transition in AS occurs. The liver evolves from an embryonic hematopoietic tissue into a fully functional metabolic organ. Hepatocytes, which constitute more than 75% of the liver’s volume, transition from a highly proliferative stage to a state of quiescence. Mis-regulation of this quiescent state results in hypertrophic growth after birth. Various studies have elucidated the molecular mechanisms through which different SFs regulate liver development, homeostasis, and metabolism in both health and disease conditions. As the mouse liver grows, the expression of IGF2BP1 gradually diminishes, leading to a shortening of the RNA poly(A) tail and a decrease in RNA stability in the fetal liver. In addition, IGF2BP1 binds to insulin-like growth factor-2 mRNA to prevent it from binding to lysine demethylase 5B (KDM5B) mRNA, decreasing the stability of KDM5B mRNA.^103^ Concurrently, there is a gradual increase in RBPs that facilitate RNA degradation, such as CSDE1 and KSRP. This indicates the dynamic regulation of AS-related RBPs during liver development.^104^

The liver, the primary site for cholesterol balance and lipid metabolism regulation, is intricately linked with the synthesis of lipoproteins, which are essential in lipid metabolism and are partially controlled by AS-related RBPs.^105^ RBFOX2, for instance, regulates a range of ASEs involved in maintaining lipid balance. These include events related to Scavenger Receptor Class B Type I (Scarb1), Phospholipase A2 Group VI (Pla2g6), NUMB Clathrin Vesicle Adapter (a component of the Sec31a COPII vesicle transport system), and Oxysterol Binding Protein 9 (Osbpl9). Besides, hepatocyte-specific RBFOX2 gene knockout results in decreased blood cholesterol levels and increased levels of cholesterol, bile acids, and other lipids in the liver, suggesting that RBFOX2 plays a pivotal role in controlling lipid distribution and could potentially be targeted for therapeutic purposes.^106^ Vatandaslar et al.^107^ utilized viP-CLIP to identify RBP targets in mouse liver and discovered that TIAL1 can target Insig2 and ApoB to regulate their ASEs, thereby controlling cholesterol synthesis and secretion.^108^ Members of the SRSF family have been extensively studied for their physiological regulation in the liver. SRSF1, for example, regulates hepatocyte lipid metabolism and transport. In mice with targeted SRSF1 deficiency, acute liver injury is associated with excessive formation of harmful RNA-DNA hybrids (R-loops), which induces DNA damage and further leads to genomic changes in hepatocytes, metabolic disorders, and acute liver injury.^109^ Besides, SRSF3 is necessary for hepatocyte differentiation,^110^ SRSF10 is involved in regulating fat formation and obesity, and SLU7 is necessary for liver homeostasis.^111^ Recent research has highlighted that the postnatal remodeling and maturation of the liver are driven by coordinated changes in cell-type-specific transcription and post-transcription. Within the liver, one of the few RBPs that is induced after birth is ESRP2. ESRP2 regulates a series of conservative AS conversions in hepatocytes, thereby managing terminal differentiation and maturation.^112^ The downregulation of ESRP2 activates neonatal ASEs, weakens Hippo signal transduction, and enhances the transcription of downstream target genes. This process promotes liver tissue regeneration.^113^ However, excessive alcohol intake can release inflammatory cytokines that significantly inhibit this process, leading to alcoholic hepatitis.^114^

Fructose and glycerol are significant components of liver nutrient metabolism. Ketohexokinase (Khk), the rate-limiting enzyme for fructose decomposition, metabolizes fructose into 1-phosphate fructose.^115^ Meanwhile, glycerol kinase (GK) operates at the interface of carbohydrate and lipid metabolism by catalyzing the conversion of glycerol to glycerol-3-phosphate.^116^ Numerous studies have demonstrated that AS-related RBPs are involved in regulating the ASEs of KhK and GK. Exons 3a and 3c undergo mutually exclusion splicing to produce two isoforms, KHK-C and KHK-A.^117^ SF3B1 can regulate the AS pattern from the low-activity KHK-A to the high-activity KHK-C, with the generation of KHK-A requiring the involvement of HNRNPH1/2.^118^ Another HNRNP family member, APOBEC1 Complementation Factor (A1CF) contains three non-identical RRMs and is a crucial component of Apolipoprotein B mRNA editing.^119^ A1CF, as a hepatocyte-specific AS-related RBP, regulates the production of liver-enriched isoforms by controlling ASE. The most significant ASE is to regulate the production of the KHK-C isoform, a process that is antagonistic with HNRNPH1/2. In addition, A1CF in liver cells regulates the retention of exon 5 in GK to promote glycerol-stimulated glucose production.^120^

#### Intestine

The deletion of the PTBP1 in the Intestinal Epithelial Cells (IECs) of newborn mice disrupts neonatal immune adaptation, leading to early colitis and colorectal cancer. In adulthood, PTBP1 suppresses the expression of Phlda3 in Paneth cells, thereby enabling AKT activation. This may maintain the plasticity of Paneth cells and support Intestinal Stem Cell (ISC) niche functions, thereby regulating the regeneration of IECs.^121,122^

In summary, the role of AS in the development of the digestive system is mainly reflected in regulating the expression and function of specific genes, affecting the metabolic process and physiological state of the liver. These research results provide important clues for us to understand the mechanism of certain disease occurrences and find new treatment strategies. Future studies could focus on elucidating the role of specific AS networks, including AS-related RBPs and downstream isozymes, in response to varying metabolic demands.

### Alternative splicing and related RBPs in the immune system

Numerous genes involved in either innate or adaptive immune signal transduction undergo varying degrees of ASE. 60% of all genes in T lymphocytes or B lymphocytes possess AS isoforms with the application of RNA sequencing and microarrays.^123^ Besides, CD45, which is present on the surface of most immune cells, is encoded by the Ptprc gene.^124^ Members of the HNRNP family have been proven to regulate the AS of cassette exons 4, 5, and 6 of the Ptprc gene. This regulation affects the activation, proliferation, and cytokine production during the development of T cells and B cells. This aspect has been detailed in a review.^125,126^ Moreover, there are a significant number of ASEs in antiviral immune response. SRSF3 and PTBP1 have been found to play key regulatory roles in this process.^127^

#### T lymphocyte

T-cell activation and the subsequent changes in protein expression, triggered by signals from antigen-presenting cells, largely depend on alterations in transcription and post-transcriptional expression. RBPs play a pivotal role in this process.^128^ During T-cell activation, there is a comprehensive reduction in intron retention, which is associated with increased mRNA homeostasis. AS regulates T-cell activation through a feedback loop involving CELF2 and the c-Jun N-terminal kinase signaling cascade (JNK signaling cascade). T-cell activation induces exon 2 skipping of the dual-specificity mitogen-activated protein kinase kinase 7 (MKK7, also known as MAPKK7). This introduces additional docking sites for JNK and strengthens the JNK pathway. The latter induces CELF2 mRNA stability and upregulates CELF2 protein expression. CELF2 binds upstream of MKK7 exon 2 to further promote its skipping, forming a positive regulatory feedback loop that enhances JNK activity and promotes T-cell differentiation and cytokine production. CD45, a crucial cell surface molecule in the process of T-cell differentiation and activation, is one of the targets of HNRNPL and undergoes HNRNPL-dependent ASEs, leading to exon 4 skipping following T-cell activation. The deletion of PTBP1 disrupts T-cell homeostasis without affecting T-cell development and PTBP1 deletion enhances dendritic cell (DC) function. In DCs with PTBP1 knocked out, 33 different ASEs are identified, among which AS of PKM and a subset of IFN response genes are regulated by PTBP1. DCs lacking PTBP1 exhibit stronger antitumor effects, further suggesting that PTBP1 could be a potential therapeutic target.^129^ A recent study highlighted that AS plays a crucial role in the transition of double-positive thymocytes to single-positive thymocytes. This primarily involves precursor transcription factors Foxa1 and Foxa2, which regulate the expression of MBNL1, H1F0, SF3B1, HNRNPA1, RNPC3, PRPF4B, PRPF40B, and SNRPD3. In CD69^+^DP cells undergoing positive selection, the double conditional gene knockout of Foxa1/Foxa2 disrupts ASEs, leading to more than 850 differentially cassette exons.^130^ Another recent study showed that SRSF1 targets 189 and 582 genes specific to Tregs and effector T cells for AS, respectively. Most of these genes are related to autoimmune diseases, further confirming the significant role of SRSF1 in protecting healthy cells and tissues from immune system attacks.^131^

#### B lymphocyte

The maturation process of B cells is intricately regulated by AS-related RBPs. The germinal center (GC), a specialized microenvironment within secondary lymphoid organs, serves as the site for B-cell affinity maturation and differentiation into long-lived memory B cells and high-affinity antibody-secreting plasma cells. The affinity maturation of GC B cells necessitates a delicate balance between antigen recognition and activation via B-cell receptors (BCR), cellular proliferation, somatic hypermutation (SHM), and clonal selection of B cells.^132^ The upregulation of PTBP1 in B cells has been demonstrated to be crucial for early B-cell selection.^133^ Upon B-cell activation, PTBP1 is indispensable for the accurate expression of MYC-dependent gene programs. It directly modulates ASEs and transcript abundance that escalate during positive selection processes, thereby promoting cellular proliferation.^134^ HuR interacts with RNA transcripts from 134 MYC-regulated genes in B cells, orchestrating a program that governs GC B-cell proliferation and Ig somatic hypermutation. Moreover, HuR regulates the AS and abundance of mRNAs necessary for entry and progression through the S phase of the cell cycle, modulates features of genes associated with DNA deamination, and safeguards GC B cells from DNA damage and cell death.^135^ During the class-switching recombination (CSR) process, which enables B cells to produce diverse antibodies, HuR depletion in activated B cells induces an imbalance in energy metabolism, leading to a lethal accumulation of reactive oxygen species, thereby compromising B-cell proliferation and CSR occurrence.^136^ Recent work^137^ has highlighted that TIA1 and TIA-like 1 (TIAL1) are crucial RBPs for sustaining long-term GC responses and generating high-affinity class-switched antibodies. TIA1 and TIAL1, equipped with three RRM, recognize U-rich elements in target mRNA introns and 3’UTR, primarily participating in ASEs and translation regulation.^138^ In GC B cells, TIA1 and TIAL1 modulate MCL1 expression at the post-transcriptional level. MCL1 is the sole member of the BCL2 family required for GC B-cell survival.^139^

Beyond lymphocytes, recent evidence has also demonstrated the regulatory role of AS-related RBPs in macrophages. Transcriptome analysis of mouse macrophage lines has identified SRSF6 as a crucial regulator of mitochondrial balance. SRSF6 governs the AS of intron 1 of BAX by directly interacting with the ESE, thereby preventing an excessive accumulation of BAX-κ that could lead to macrophage death. Upon pathogen detection, macrophages modulate SRSF6 expression to control the release of immunogenic mtDNA and adjust the threshold for initiating programmed cell death.^140^ QKI-5 plays a role in macrophage differentiation.^141^ During the progression of atherosclerosis, QKI-5 serves as a dynamic regulator of ASE and expression profiles, driving monocyte activation, adhesion, and differentiation into macrophages, thereby contributing to disease progression.^142^

In summary, during the development of the activation immune system, RBPs are crucial as they regulate gene expression at the both transcriptional and post-transcriptional process, and the deletion of certain RBPs such as PTBP1 significantly disrupts immune cell homeostasis, highlighting the importance of AS-related RBPs. Continued exploration is of significant value, including their participation in immune signal transduction and the impact of differential ASEs within genetic regulatory mechanisms on physiological development. Furthermore, understanding the role of ASEs in the process of antigen presentation is also crucial.

### Alternative splicing and related RBPs in the cardiovascular system

Research on AS and AS-related RBPs in vascular development is somewhat limited.

NOVA2 has been identified as a tissue-specific regulator of AS-related RBPs expressed in vascular endothelial cells, influencing vascular morphogenesis^143–145^ and lymphatic endothelial cell specification.^146^ NOVA2 directly binds to L1CAM pre-mRNA, leading to the exons skipping related to the L1CAM transmembrane structure in ECs. This results in the release of soluble L1-ΔTM, which exhibits potent angiogenic function through autocrine and paracrine activities. In addition, NOVA2 regulates the Ppar-γ exon B and exon 5, and E2F dimerization partner 2 (Tfdp2) exon 7 inclusion. The isoforms produced by the former are associated with angiogenesis and vascular development, while those produced by the latter are involved in regulating cell apoptosis, angiogenesis, adipogenesis, and cell migration.^147^ NOVA2 also regulates UNC5B to skip exon 8 and produce UNC5B-Δ8 in ECs. The latter cannot transduce Netrin-1 signals and regulates blood vessel formation in a death-dependent manner.^148^ Endothelial cells lacking NOVA2 exhibit increased MAPK/ERK signal transduction. Prox1 expression is dynamically controlled by ERK signal transduction, playing a role in regulating lymphocyte differentiation.^149^ QKI-5 also plays a role in angiogenesis by binding with Myocardin and regulates its expression and ASEs, activating contractile protein expression to ensure vascular smooth muscle cell (VSMC) physiological function.^150^ Lack of QKI-5 leads to defects in VSMC generation and causes embryonic lethality in mice.^151^

The generation of red blood cells, a process that starts with the differentiation of hematopoietic stem cells, relies on both transcriptional and post-transcriptional programs to drive the synthesis of stage-specific proteomes.^152^ This process gradually refines the function of cells. During erythropoiesis, the expression level of HNRNPAB decreases.^153^ In parallel, RBM39 interacts with U2AF65 and SF3b155 to form a complex that recruits U2 snRNP to the BPS by binding to TIA1 and Pcbp1, facilitating the recruitment of U2 snRNP to branch points. Both processes foster stage-specific conversion (including both inclusion and skipping) of exon 16 in the gene encoding protein 4.1 R via AS, thereby regulating erythrocyte membrane stability.^154^ In addition, MBNL1, a regulator of AS transitions within coordinated AS networks, operates at the culmination of erythropoiesis. During the terminal development of mouse red blood cells, MBNL1 encourages the inclusion of a 35-nucleotide box-type exon in nuclear distribution protein nudE-like 1 (NDEL1). Notably, only NDEL1 isoforms that contain this alternative exon can partially rescue differentiation defects observed following NDEL1 deletion. These defects are similar to those observed when MBNL1 is deleted. RBM38, another AS-related RBP induced in late-differentiating erythroblasts, is related to the translation initiation factor eIF4G, and promotes the translation of select mRNAs with decreasing mRNA levels.^155^

AS also plays a crucial role in heart development. A comprehensive review of the crucial role of AS-related RBPs in maintaining normal heart morphology and function highlights the importance of the correct expression of AS isoforms in the heart for the regulation of AS networks. Key regulatory roles in normal heart morphology and function are played by CELF, MBNL1, RBM24, SRSF1, SRSF2, SRFS10, and HNRNPU.^156^ Furthermore, RBM20 and PTBP1 show combined effects in selecting specific exons in cardiac tissue, and the role of RBM20 in cardiovascular diseases seems to be vital.^157^ Recently, features of PTBP1 co-localized with endothelial cells during ventricular cavity development have been revealed.^158^ By regulating endothelial cell migration and cardiomyocyte proliferation, endothelial-specific knockout of PTBP1 leads to left ventricular noncompaction (LVNC). In endothelial cells with PTBP1 defects, changes in the expression ratio of two ARRB1 isoforms are observed, which has been proven to affect endothelial cell migration. RBM24 is crucial for myocardial development. More than 4000 erroneous ASEs occur in RBM24-/-hESCs leading to myofibrillogenesis stalling at an early pre-myofibril stage causing sarcomere disruption. At different stages of cardiac differentiation, RBM24 promotes the inclusion of α-actinin 2 exon 6, which is crucial for sarcomere assembly and integrity.^159^ Furthermore, RBP with multiple splicing (variants) 2 (RBPMS2) is a conserved AS-related RBP in zebrafish and human cardiomyocytes for AS, myofibril organization, and calcium handling, participating in the regulation of cardiac AS networks.^160^ QKI, another RBP recently been identified as a pivotal regulator in cardiovascular development, specifically modulates the ASE of Z-line structural genes, including ACTN2. This involvement aids in the formation of myofibril structures within cardiomyocytes. Furthermore, QKI orchestrates the interplay between the sarcomere cytoskeleton and the cell membrane, highlighting its integral role in cellular structure and function.^161^

In conclusion, AS-related RBPs play a multifaceted role in the normal development of the cardiovascular system. A recent study has highlighted the involvement of a cardiac-specific and conserved long noncoding RNA (lncRNA) in regulating ASEs, thereby contributing to the maintenance of normal cardiac function.^162^ Research on ASEs within the cardiovascular system holds significant research potential. Moreover, a more detailed mapping of the ASE spectrum during cardiac development could provide valuable insights into the transition from fetal to adult cardiovascular system.

### Alternative splicing and related RBPs in motor system

Within the motor system, AS and associated RBPs are believed to contribute to muscle development, the regulation of muscle function, and the enhancement of athletic performance. However, it’s important to note that research specifically addressing the role of AS within the motor system is currently somewhat limited.

Smooth muscle cells and skeletal muscle stem cells^163,164^ are subject to regulation by AS. PTBP1 plays a pivotal role in this process. PTBP1 inhibits multiple smooth muscle-specific exons. During the cell differentiation stage, downregulation of PTBP1 expression leads to increased intron retention, introduction of PTCs, and alternative use of polyadenylation. AS and AS-related RBPs also play a significant role in the differentiation pathway of mesenchymal stem cells (MSCs). During the differentiation process of MSCs into osteoblasts, adipocytes, and chondrocytes, AS plays a key role in regulating MSC proliferation and cell fate determination. This aspect has been detailed in reviews.^165^ HNRNPF/H regulates the AS of transcription factor E protein family member TCF3(E2A), which can bind to the promoter of downstream genes regulating embryonic stem cell (ESC) differentiation. HNRNPF/H regulates the retention of TCF3 exon 18a by binding to TCF3 ISS after binding with PTBP1, which promotes the retention of E12 expression in human ESCs, stabilizing CDH1 and thus maintaining human ESC pluripotency.^166^ At low HNRNPF/H levels, PTBP1 mediates TCF3 pre-mRNA exon 18b retention. At high HNRNPF/H levels, it promotes TCF3 exon 18a retention.^167^ A detailed summary of the regulation of adult stem cell quiescence^168^ has revealed that QKI conditional deletion leads to ITGA7 and NUMB AS, causing inhibition of skeletal muscle stem cell activation and a decrease in asymmetric division. In addition to the above-mentioned regulatory roles of AS-related RBPs on cell differentiation, QKIs are also important cell differentiation regulatory proteins. They affect the differentiation process of neural stem cells,^169^ vessels,^151^ and muscles^170^ a series of mechanisms including regulating AS. This aspect has been detailed in reviews by Neumann et al.^171^ Furthermore, RBM24 has been scientifically validated as a regulator of muscle-specific ASEs. It possesses the ability to counteract the exon inclusion instigated by PTBP1 and HNRNPA1/A2. Notably, any defects in RBM24 can result in developmental anomalies in both the myocardium and skeletal muscle.^172^

Looking ahead, comprehensive research into AS and its associated RBPs within the motor system could offer fresh insights into the physiological mechanisms. This could also pave the way for the development of innovative strategies aimed at enhancing athletic performance.

### Alternative splicing and related RBPs in endocrine system and metabolism regulation

Compared to the extensive research on the role of AS and AS-related RBPs in cell-type specification and differentiation, studies on their contribution to metabolic regulation are relatively limited.

SAM68, initially identified as a target of tyrosine kinase c-SRC,^173^ is a member of the STAR family and plays a role in RNA processing featured with a KH domain that can bind to U(U/A)AA motifs.^174^ Mice with a SAM68 knockout exhibit reduced commitment of adipocyte progenitor cells and diminished accumulation of adipose tissue, including White Adipose Tissue (WAT) and Brown Adipose Tissue (BAT). In addition, these knockout mice also prevent obesity induced by a high-fat diet. Whole-genome exon expression analysis revealed numerous ASEs. Specifically, it produces a truncated form of the mTOR variant that inhibits the expression of functional mTOR and disrupts mTOR signal transduction, leading to defects in adipogenesis. Moreover, SAM68 is also associated with AS of Rps6kb1. SAM68 knockout leads to the production of a new transcript isoform Rps6kb1-002 in preadipocytes, promoted by SRSF1, thereby inhibiting adipogenesis and lipid accumulation.^175^ Chao et al.^176^ and Zhang et al.^177^ integrated existing research results with specific ASEs and different functions of various AS regulatory factors as examples, highlighting the important role of AS mechanisms in adipogenesis and adipocyte biology. Among them, HuR, PSPC1, Sam68, RBM4, Ybx1, Ybx2, IGF2BP2, KSRP, and other RBPs play a key regulatory role. Recently, Peng et al.^178^ used direct RNA sequencing technology to detect bovine adipocytes. From the aspects of transcript/isoform, poly(A) tail length, and modification, they systematically analyzed the changes in the transcriptome during adipogenesis, revealing numerous RNA changes related to AS. The study of AS-related RBPs in lipid metabolism regulation is of great significance. Other RBPs may also be responsible for other important metabolic pathways for further regulation of AS. Accurate molecular understanding of these processes in normal physiology and disease may reveal new targets for the treatment of metabolic disorders.

In the endocrine system and metabolic regulation, AS and AS-related RBPs play a pivotal role. RBPs orchestrate a series of post-transcriptional events, such as AS, stability, localization, and translation. These processes significantly influence RNA processing and metabolism, thereby altering the destiny and functionality of RNA. There exists a need for more research on the functional mechanisms of specific AS variants in different types of adipose tissue in the pathogenesis of obesity, and targeting AS-related RBPs to treat lipid metabolism disorders has prospects.

In addition to their established roles in tissue differentiation and development, the impact of AS-related RBPs in other systems has been less extensively studied. Within the respiratory system, HNRNPA1 and HuB enhance the interaction between the alveolar epithelium and vascular endothelium in mice. This is achieved by modulating ASE, which is crucial for the maturation of lung respiratory function post-birth.^179^ Furthermore, proteins such as FOX2, TIAR, and HUB contribute to fetal alveolar maturation. They regulate the production of the Jma isoform of ErbB4 in alveolar type II epithelial cells.^180^ During the development of tissue and organs, as well as their physiological functions, alterations among AS isoforms are particularly prevalent. To fully comprehend the functional role of developmental AS networks, it’s necessary to identify the structure and function of thousands of physiological AS changes related to development more extensively. This requires the integration of whole-genome methodologies with molecular research across various systems to thoroughly determine the functional impact of physiological AS conversion.

## Pathological functions of AS-related RBPs in tumor and non-tumor diseases

**Fig. 4:**
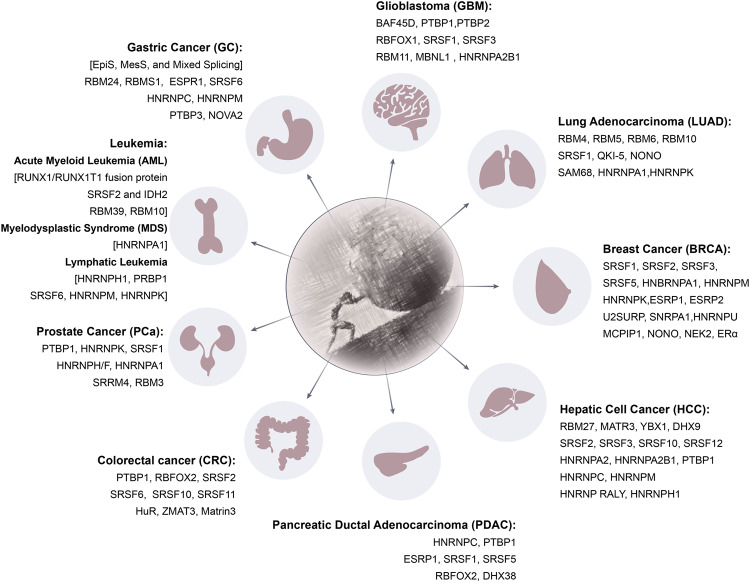
“Sisyphus and the Stone”: AS-related RBPs in oncology. AS is a process that occurs during the post-transcriptional stage of gene expression, enabling a single gene to generate multiple mRNA variants, thereby enhancing protein diversity. AS-related RBPs influence the destiny of mRNA through various functional mechanisms within this process. In numerous types of cancer (this review concentrates on glioblastoma, lung adenocarcinoma, breast cancer, hepatocellular carcinoma, pancreatic ductal adenocarcinoma, colorectal cancer, prostate cancer, leukemia, and gastric cancer), alternative splicing and RNA-binding proteins play pivotal roles. Notably, members of the HNRNP and SRSF families exert significant functions and have been extensively documented in the literature. This figure was drawn by Adobe Illustrator

AS plays a pivotal role in augmenting transcriptome complexity, and its dysregulation is implicated in a myriad of human diseases.^23^ A substantial body of research is centered on abnormal ASEs in the context of cancers. In the realm of non-tumor diseases, neurodegenerative diseases and autoimmune diseases attract relatively more attention. The subsequent sections will delve into AS-related RBPs from the two major perspectives, tumors (Fig. 4) and certain non-tumor diseases.

### AS and related RBPs among the common tumors

HNRNPs and SRSFs serve as the primary regulators of AS site selection and their dysregulation is observed in various cancers, emphasizing the importance of AS.^181–184^ Proteins that form the core of the AS mechanism interact with other RBPs to create complexes, determining tissue and tumor-specific ASEs. Depending on the relative position of the RBP-binding site and the regulated exon, these proteins can either synergize or antagonize the activity of the spliceosome under different circumstances. Abnormal ASEs are widely observed in various biological processes of tumors, including EMT, apoptosis, cell cycle, proliferation, metabolism, stress, immune evasion signaling, and invasion.^185^ Dysfunctional SFs can act as oncogenes or tumor suppressor genes during tumor progression.^186^ Currently, there are relatively few reviews on the promotion/inhibition of tumor occurrence and development by AS-related RBPs. Here, we provide an overview of recent research in the field of tumors, offering a new perspective on tumor diagnosis and treatment.

### Glioblastoma (GBM)

Immunohistochemical staining of GBM tissues has revealed that AS-related RBPs are integral components of tumor tissues.^187^ As research progresses, abnormal expression of AS regulatory factors has been confirmed to play a crucial role.

PTBP1, one of the most important AS-related RBPs, is overexpressed and promotes tumor cell proliferation and angiogenesis in GBM. PTBP1 promotes annexin A7 (ANXA7) exon 6 inclusion, producing ANXA7-I2 isoform, which inhibits the degradation of receptor tyrosine kinase (RTK) epidermal growth factor receptor (EGFR) caused by endocytosis-dependent pathway degradation^188^ and leads to sustained activation of the EGFR signaling pathway.^189,190^ EGFR, PDGFRA, and MET proto-oncogene are observed in GBM cells with ANXA7-I2 highly expressed.^191^ It suggests that PTBP1 promotes the RTK signaling pathway sustained activation by regulating ANXA7 AS, inducing GBM angiogenesis, cell proliferation, and migration. PTBP1 also regulates reticulon 4 (RTN4) exon 4 exclusion, leading to an increase in RTN4-B isoforms, the isoform widely expressed in human tissues, thereby promoting endothelial cell migration and vascular remodeling.^192^ In GBM, RTN4-B isoform expression is upregulated, causing EMT, vascular proliferation, and migration activity.^95^ Recently, a unique feedback mechanism between PTBP1 and BRG1-associated factor 45D (BAF45D) was discovered. The latter is a component of the switch/sucrose non-fermenting (SWI/SNF) complex and is important for the differentiation and maturation of the central nervous system.^193^ Once BAF45D is separated from the SWI/SNF complex, mitosis and differentiation of neural precursor cells are inhibited.^194^ BAF45D pre-mRNA exons 6, 6A, and 7 are potential splice sites targeted by PTBP1. Inhibition of PTBP1 expression can shift BAF45D/6A- to BAF45D/6A+ in GBM cells, a change that has been demonstrated in in vivo experiments to significantly prolong mouse survival.^195^ MAP/microtubule affinity-regulating kinase 4 (MARK4), another target gene regulated by PTBP1, is widely expressed in mammals and relatively higher in brain tissue.^196^ PTBP1 regulates the MARK4 exon 16 exclusion, generating the isoform highly enriched in proliferating undifferentiated cells.^197^ PTBP1 knockout promotes neural differentiation of GBM cells through the UNC5B receptor, thereby inhibiting the proliferation of cancer cells.^198^

Other members of the HNRNP family also play a key role in GBM. HNRNPA2B1, for instance, promotes several exons exclusion, including recepteur d’Origine Nantais (RON) exon 11, insulin receptor (INSR) exon 11, CASP8 and FADD-like apoptosis regulator (CFLAR; C-FLIP) exon 7, caspase 9 (CASP9) exons 3–6 as well as WW domain-containing oxidoreductase (WWOX) exons 6–8.^199^ Inhibition of HNRNPA2B1^200,201^ reduces GBM cell vitality, adhesion, migration, and invasion. It also leads to the inhibition of STAT3 activation and downregulation of overexpressed cell cycle protein D1, proliferating cell nuclear antigen (PCNA), Fos oncogene (C-fos), Myc oncogene (C-MYC; MYC), Pim-1 oncogene (PIM1), BCL2 apoptosis regulator (BCL2), Bcl-XL, vascular endothelial growth factor (VEGF), and matrix metalloproteinase 2 (MMP2).^18,202^ Besides, overexpressed HNRNPH/F regulates abnormal AS of death domain regulatory protein insulinoma-pancreatic euglycemic protein 20 (IG20), RON gene exon 11 and a-RAF oncogene, inhibiting tumor cell apoptosis and promoting proliferation and migration.^203–205^

Members of the SRSF family also play a crucial role in AS in GBM. SRSF1, for instance, induces exons 23 and 24 retentions in MYO1B pre-mRNA, leading to an increase in the full-length isoform, MYO1B-fl, which recruits phosphatidylinositol-4,5-bisphosphate 3-kinase (PI3K) to the cell membrane, thereby leading to overactivation of pyruvate dehydrogenase kinase 1 (PDK1)/AKT and P21(RAC1) activated kinase (PAK)/LIM domain kinase (LIMK) signaling pathways and promoting GBM cell proliferation and migration.^206^ In addition, SRSF1 has been found to regulate the exon 14B exclusion of serine/threonine kinase 2 (MNK2), producing a large amount of the MNK2B isoform, which lacks the MAPK domain and therefore cannot activate p38α-MAPK-induced cell apoptosis, but phosphorylates EIF4E to accelerate tumor occurrence.^207–209^ SRSF3, another member of the SRSF family, regulates exon 7 exclusion in ETS variant 1 (ETV1) and exon 9 exclusion in nudE neurodevelopment protein 1 (NDE1), leading to ETV1 not being degraded by the ubiquitin-proteasome pathway, and an increase in NDE1-SSSC isoform. Abnormal activation of NDE1-SSSC promotes mitotic spindle formation leading to continuous proliferation of tumor cells.^210^ SRSF3 also regulates the ratio of tumor protein P73 (TP73) isoforms (TP73α, β, γ, ε, and ζ). TP73α has a pro-tumor effect, while TP73β and TP73γ are associated with cancer suppression.^211^ Furthermore, SRSF3 regulates the PDGF-PDGFRB pathway and its downstream AKT and ERK abnormal activation by regulating TP73 AS as an intermediate hub, promoting GBM cell migration, proliferation, and angiogenesis.^212^

Other AS-related RBPs have also been found to exert key regulatory effects on ASEs in GBM. Rapid proliferation of GBM often accompanies a significant amount of cell apoptosis.^213^ Apoptotic GBM cells promote the proliferation and drug resistance of surviving tumor cells by secreting apoptotic extracellular vesicles (appoEVs) rich in RBM11, a member of the RBM family, affects abnormal ASEs of MDM4 and Cyclin D1, generating Cyclin D1a and MDM4s isoforms that are conducive to carcinogenesis. Additionally, MBNL1 which is highly expressed in astrocytes, serves as a tissue-specific RNA metabolic regulator.^214^ It participates in transcriptional regulation by stabilizing, splicing, polyadenylating, and localizing target mRNA.^215^ MBNL1 pre-mRNA itself also undergoes AS,^216^ and its dysregulation will lead to many targeted genes undergoing ASEs from adult to fetal.^217^ MBNL1 is inhibited by hypoxia,^218^ promoting the maintenance of glioblastoma stem cells and immune evasion of GBM cells. The autoregulation effect on MBNL1 pre-mRNA exon 5 is also inhibited. The active form of MBNL1 inhibits the self-renewal of GBM stem cells in vitro and inhibits their tumorigenic potential in vivo. Furthermore, inducing GBM ferroptosis is considered a research direction with therapeutic value.^219^ Sun et al.^220^ found that NF-κB activating protein (NKAP) binds with SLC7A11 m6A, recruits AS factors to proline-rich and glutamine-rich (SFPQ) recognition splice sites, performs transcription termination site (TTS) ASEs on SLC7A11 transcripts, inhibiting GBM ferroptosis. Wang et al.^221^ used TCGA and CGGA public databases to screen differentially expressed mRNA ASEs and found that NONO promotes GPX1 intron retention, stabilizes tumor redox balance, and promotes tumor growth and invasion.

AS-related RBPs also exhibit complex interactions in GBM. PTBP1 is highly expressed in undifferentiated neural stem cells, while PTBP2 is a marker of differentiated neurons.^222^ PTBP1, which is highly expressed in undifferentiated neural stem cells, regulates PTBP2 pre-mRNA exon 10 AS, leading to NMD, thereby resulting in the reduction of PTBP2 expression, inhibition of the differentiation and maturation of neural stem cells and NPCs and promoting tumor occurrence.^96^ On the other hand, PTBP2 is a marker of differentiated neurons. In GBM, the expression of SON DNA and RNA-binding protein (SON) increases, promoting efficient AS of PTBP1 pre-mRNA. HNRNPA2B1 forms a complex with SON, promoting SON recruitment to multiple AS sites, and inhibiting the antagonistic effect of RBFOX1 and PTBP1. These complex interactions highlight the intricate nature of AS regulation in GBM.^95^

Recently, Zhao et al.^223^ used the TCGA database based on ASE features to divide GBM into two subtypes. These subtypes exhibit significant differences in immune infiltration, angiogenesis, and treatment response. Taken together, these studies demonstrate the diagnostic and therapeutic value of AS and AS-related RBPs in GBM. The screening of specific RNA ASEs or RBPs to mitigate malignant progression following GBM treatment offers a theoretical foundation. Future research, especially those focusing on GBM molecular subtyping based on ASEs, will significantly contribute to the advancement of diagnosis and treatment strategies. From a therapeutic perspective, efforts could be made to regulate the ASEs associated with VEGF, thereby enhancing the therapeutic efficacy of Bevacizumab,^224^ a medication that specifically targets VEGF.

### Breast cancer (BRCA)

BRCA is one of the most common malignant tumors among women,^225^ with triple-negative breast cancer (TNBC) posing a significant threat to women’s health due to its lack of effective treatment targets and propensity for recurrence and metastasis.^226^ A comprehensive analysis of global ASEs has recently revealed ASEs related to tumor occurrence and the immune microenvironment in TNBC. The association between AS-related RBPs and tumor-related ASEs is elucidated, establishing a prognostic model based on survival-related ASEs and providing potential targets for subsequent basic research and clinical translation.^227^

In 2021, a detailed review revealed the abnormal AS of BRCA1, HER2, Kruppel-like factor 6 (KLF6), ER alpha (ERα), ER Beta(Erβ) in BRCA, and the regulatory mechanisms of RBPs, including SRSF1, SRSF2, SRSF3, SRSF5, HNBRNPA1, HNRNPM, HNRNPK in tumor progression.^228^ Recent research on AS and AS-related RBPs in BRCA are emerging. Under hypoxic conditions, SRSF2 expression is inhibited by miR-222, leading to the accumulation of MBD2 isoform MBD2a, thereby promoting the expression of FZD1 and activating the Wnt/β-catenin signaling pathway. This process enhances the EMT and metastatic ability of BRCA.^229^ Besides, MYC amplification is one of the most frequently mutated oncogenes in BRCA, and AS-related RBPs play a role in its signaling pathway.^230^ SRSF1 directly binds to PTPMT1, SMARCD1, GAB1, and TERF1 pre-mRNA, promoting the inclusion of exons in PTPMT1, FER, and SMARCD and the exclusion of exons in GAB1 and TERF1. SRSF1 directly binds to the PTMT1 exon 3, leading to the production of the long isoform and resulting in carcinogenic effects through the AKT/C-MYC axis.^231^ Deng et al.^232^ studied MYC-regulated ASEs in TNBC and found that MYC enhances U2SURP translation, a poorly defined member of the SRSF family, through eukaryotic translation initiation factor 3 subunit D (eIF3D)-dependent mechanism, leading to an accumulation of U2SURP in TNBC and promoting SAT1 AS by removing intron 3. This increases SAT1 mRNA stability and expression level, enhancing the carcinogenic potential and malignant phenotype of TNBC cells. Interestingly, SRSF1 has anti-cancer effects in BRCA. Yu et al.^233^ reported that circRPAP2 can bind with SRSF1 and competitively bind PTK2 pre-mRNA. This weakens ASE of PTK2 mediated by SRSF1 (the effector of SRSF1 carcinogenic activity), leading to a decrease in PTK2 mRNA and protein expression, and thereby inhibiting cell proliferation, migration, and survival in tumor progression.^234,235^

Recently, a computational framework called pyTEISER has been applied in BRCA to identify RNA regulatory structural elements.^236^ A new AS enhancer site is confirmed to be directly bound by RBP SNRPA1, functioning independent of spliceosome function and regulating the inclusion of S3E exons, which is highly correlated with BRCA metastasis. In addition, HNRNPU, upregulated in BRCA, significantly in TNBC, binds with DEAD-box helicase 5 (DDX5) protein to regulate MCM10 pre-mRNA intron retention between exons 19 and 20, reducing NMD and activating the Wnt/β-catenin signaling pathway by increasing MCM10 mRNA stability and expression.^237^ Other RBPs also play an important role in the development of BRCA. Monocyte chemotactic protein-induced protein 1(MCPIP1), a zinc finger structure domain RBP,^238^ regulates AS in TNBC. Compared with normal tissues and cell lines, MCPIP1 is downregulated in TNBC. MCPIP1 regulates nuclear factor IC (NFIC) AS to promote CTF5 synthesis. The latter inhibits cell cycle protein D1 expression and downregulates its downstream signal transduction targets p-Rb and E2F1, participating in MCPIP1-mediated anti-proliferative effects.^239^ In addition, LIN28 binds with more than 800 RNA in BRCA cells, suggesting its important regulatory role in AS. Cells lacking LIN28 undergo significant ENAH gene isoform conversion, which is closely related to the HER+ breast cancer subtype and regulates tumor progression.^240^ Kim et al.^241^ identified a TNBC-specific RBP–NONO, which regulates STAT3 expression by directly interacting with STAT3 RNA and protein. NONO directly binds to the STAT3 RNA region and recruits STAT3 protein to STAT3 target promoters (such as CCND1 promoter). NONO also regulates the transcriptional activity and stability of STAT3, thereby promoting TNBC cell proliferation and chemotherapy resistance. Furthermore, high expression of NONO is independently associated with poor prognosis in BRCA patients. By binding with the mRNA of cell proliferation-related genes (including S-phase kinase 2 and E2F transcription factor 8), it regulates the expression of these genes at the post-transcriptional level.^242^ NEK2, a non-typical RBP[265], has been found to have a broad impact on AS regulation in TNBC, mainly involved in regulating the inclusion of cassette exons, including MYO18A exon 48, SORBS1 exon 12, and SPAG9 exon 30 in TNBC patients is associated with poor prognosis, which promotes TNBC cell invasive phenotype and promotes EMT.^243^

Patients with BRCA often face a poor prognosis due to drug resistance.^244^ Tamoxifen resistance is common in ER + BRCA patients. ESRPs, including ESRP1 and ESRP2, which are linked to cancer invasion, metastasis, and AS program regulation,^139^ significantly slow down the growth rate of ER + BRCA and alter EMT AS characteristics when ESRP1 is knocked out in vitro, as opposed to ESPR2.^245^ The knockout of ESRP1 in tamoxifen-resistant cells affects lipid metabolism and oxidoreductase processes, leading to a significant decrease in fatty acid synthase (FASN), stearoyl-CoA desaturase 1 (SCD1), and phosphoglycerate dehydrogenase (PHGDH) at both mRNA and protein levels. Interestingly, ERα, present in more than 70% of BRCA,^246^ has recently been identified as an important non-typical RBP involved in ASEs.^247^ ERα promotes cell survival and maintains tamoxifen resistance at the post-transcriptional level. By inducing MCF-7 cells to downregulate ERα with siRNA, it was found that the unconnected form of ERα (apoERα) regulates ASEs to maintain the lumen phenotype of BRCA cells.^248^ ERα controls the ASE of XBP1 mRNA, a key component of the UPR pathway, and regulates the translation of stress response proteins such as eIF4G2 and MCL1 mRNA. Recent reports have highlighted alterations in AS patterns in BRCA chemotherapy resistance.^249^ Lysine acetyltransferase 5 (Tip60), a gene related to cisplatin resistance in various cancers, is involved in BRCA cisplatin resistance through SRPK1 acetylation reduction. SRPK1 is another protein that regulates RBPs post-transcriptional modification.^250^ This led to increased phosphorylation of SRPK1 and SRSFs and induced anti-apoptotic variants of some genes involved in cell apoptosis.

In summary, AS-related RBPs play a pivotal role in regulating various phenomena in BRCA, including proliferation, metastasis, and drug resistance. A study leveraged the TCGA and TCGASpliceSeq databases to conduct a bone metastasis-specific AS analysis on BRCA, resulting in a survival model comprising 15 overall survival-related splicing events (OS-SE).^251^ Subsequent research can further expand the inclusion of breast cancer bone metastasis samples to improve the analysis of ASEs and validate the results through functional experiments.

### Lung adenocarcinoma (LUAD)

Lung cancer, one of the most common and deadly tumors worldwide,^225^ has a subtype known as LUAD that is responsible for nearly half of all lung cancer deaths. The prognosis for patients is generally poor due to the lack of effective treatment strategies. Changes in the expression of RBPs such as QKI, RBM4, RBM5, RBM6, RBM10, and SRSF regulate many of the most common abnormal ASEs in lung cancer, as detailed by Coomer et al.^252^ Therefore, here we primarily summarize the significant research advancements in this field over the past 3–4 years.

Liu et al.^253^ screened public databases for LUAD-related RBPs associated with patient prognosis and found that an AS pattern involving 16 SRSF family members strongly correlates with tumor microenvironment (TME) characteristics such as immune inflammation, immune rejection, and immune apoptosis. This discovery significantly deepens our understanding of AS-related RBPs in the TME. In a bid to explore and develop new treatment methods for LUAD, Wu et al.^254^ integrated multi-omics data to deeply investigate abnormal ASEs in LUAD and identified a more specific immunogenic LUAD subtype. This research has advanced the role of abnormal AS in the tumor immune microenvironment in LUAD, laying a solid foundation for future exploration of new treatment targets.

QKI, one of the most downregulated AS factors in lung cancer, is associated with poor prognosis. In normal cells, QKI selectively inhibits exon 12 inclusion in NUMB mRNA by competing with the core splicing factor SF1.^255^ NONO may play a synergistic role in this process by promoting the skipping of exon 9 in NUMB mRNA, thereby increasing the expression of NUMB isoforms that can inhibit cell proliferation and Notch activity.^252,256^ In addition, QKI-5, a member of the QKI family, controls ASEs of Adducin transcript. Adducin is a membrane skeleton protein family encoded by ADD1, ADD2, and ADD3 genes. QKI-5 partially suppresses cell proliferation and migration by inhibiting exon 14 inclusion in ADD3. The common downregulation of QKI-5 seen in lung cancer results in a loss of its tumor-suppressive effect.^257^ SAM68 also plays a significant role in LUAD ASEs. It is upregulated in LUAD compared to adjacent non-tumor tissues and stimulates HNRNPA1-dependent PKM AS and subsequent formation of PKM2 isoforms, thereby promoting cancer metabolism and tumorigenesis.^258^ RBM10, another AS-related RBP, is often downregulated in LUAD. Frequent mutations in RBM10 result in the loss of its tumor suppression function, promoting tumor progression.^259,260^ RBM10 is involved in regulating the ASE of the mitochondrial apoptosis regulator Bcl-x, reducing the ratio of pro-apoptotic Bcl-xS to anti-apoptotic Bcl-xL. The inactivation of RBM10 reduces cell apoptosis induced by EGFR inhibitors, highlighting the mechanism of AS in tumor cell autophagy and providing a new avenue for potential treatment.^261,262^ ASEs related to RBM10 mutation in LUAD and RBM10 deficiency in LUAD result in significant upregulation of EIF4H-L expression, aiding in the proliferation and survival of LUAD cells.^263^ EIF4H has been proven to encode a translation initiation regulator and is closely related to cancer.^264^ Furthermore, SRSF1 and RBM10 may have similar roles.^261^ SRSF1 is known to regulate Bcl-x AS towards longer isoforms, thereby inhibiting autophagy through its interaction with Beclin1. The reduction in SRSF1 leads to an increase in Bcl-xS production, disrupting such interaction. Moreover, SRSF1 directly interacts with PIK3C3, disrupting the binding between Beclin1 and PIK3C3. The elimination of SRSF1 hinders the progression of gefitinib-resistant cancer cells by triggering autophagy.^261^ Besides, an important interaction pattern has been observed among AS-related RBPs, involving the non-classical activation of nuclear AURKA, which promotes the carcinogenic RNA splicing of tumor suppressor RBM4 under the guidance of m6A reader YTHDC1. The nuclear translocation of AURKA interrupts the binding between SRSF3 and YTHDC1, resulting in the inhibition of RBM4-FL generation induced by the m6A-YTHDC1-SRSF3 complex and thereby eliminating the inhibition of SRSF1-mTORC1 signaling pathway activity led by RBM4-FL. In addition, AURKA recruits HNRNPK to YTHDC1, leading to m6A-YTHDC1-HNRNPK-dependent exon skipping and subsequent production of RBM4-S. This process collectively contributes to tumor progression.^265^

LUAD is characterized by a significant number of abnormal ASEs. Analyzing and screening suitable AS-related RBPs and tumor-related ASEs can help elucidate the complex immune microenvironment of LUAD and the impact of disordered AS. The mechanisms underlying AS in lung cancer are still not fully understood. Summarizing the molecular characteristics of LUAD based on ASEs could pave the way for discovering new treatment strategies targeting LUAD.

### Gastric cancer (GC)

GC, the second leading cause of cancer-related deaths worldwide,^266^ results in poor patient prognosis due to the development of chemotherapy resistance, which leads to tumor recurrence.^267^ An integrated analysis of ASEs in GC reveals genes with prognostic mRNA expression and/or ASEs and validated key ASEs and related RBPs involved in GC progression.^268^

A systematical analysis of 83 cases of GC with normal mucosa matched, has classified GC into three subtypes based on ASEs: Epithelial Splicing (EpiS), Mesenchymal Splicing (MesS), and Mixed Splicing, with the crucial role of RBM24, RBMS1, and ESPR1 in the progression of GC underscored.^269^ This is the first study to propose a stratification scheme for GC patients based on ASEs, which holds significant potential for subsequent precision treatment. Recent research has shown that the binding of SRSF6 with lncRNA colorectal neoplasia differentially expressed (CRNDE) leads to abnormal physiological regulation of the AS of SRSF6, which results in resistance to oxaliplatin and 5-FU treatment both in vivo and in vitro.^270^ NOVA2 is generally upregulated in GC cells and produces new AS transcripts, including causing its downstream target Rap Guanine Nucleotide Exchange Factor 6 (RapGEF6) exon 21a inclusion, playing an important role in tumor angiogenesis.^271^

Metastasis of GC is another significant factor contributing to poor patient prognosis,^272^ with AS playing a crucial role. ESRP1, a key component in tumor EMT, is generally downregulated in GC metastasis events. This leads to an increase in the expression of truncated forms of exon 7 downstream leucine-rich repeat Fli-1-interacting protein 2 (LRRFIP2), further regulating coactivator-associated arginine methyltransferase 1 (CARM1) histone methylation activity and leading to tumor metastasis.^273^ Besides, when HNRNPC binds with LINC00924, the binding of HNRNPC with Mnk2 pre-mRNA exon 14a is enhanced, thereby inhibiting the ASE of Mnk2a, and regulating the p38 MAPK/PPARα signaling pathway, further promoting tumor metastasis.^274^ PTBP3 is also upregulated in GC among patients with lymph node metastasis. PTBP3 binds with the caveolin 1 (CAV1) CU-rich region to regulate ASEs, activating steroid receptor coactivator (Src) and focal adhesion kinase (FAK) proteins to promote GC lymphatic metastasis.^275^ In addition, HNRNPM binds with circURI1 (unconventional prefoldin RPB5 interactor 1) in GC to inhibit tumor metastasis, which may serve as a self-protection mechanism. After the two bind together, they regulate the ASEs of genes involved in cell migration, thereby inhibiting GC metastasis.^276^

In summary, research on AS and GC has revealed a significant correlation between the two. However, our understanding of the role of AS in the development of GC remains limited. Moreover, the potential of employing AS-related RBPs for the diagnosis and treatment is yet to be fully realized. Future research endeavors should aim to further elucidate the relationship between AS and gastric cancer, and uncover the molecular mechanisms of alternative splicing in cellular growth processes.

### Leukemia

In the early stages of myelodysplastic syndromes (MDS) and chronic lymphocytic leukemia (CLL), AS mechanisms often undergo mutations, thus highlighting their importance to cell function.^277–279^ Abnormalities in AS are primarily attributed to genetic changes that affect AS-related RBPs.^280^ Mutations most commonly occur in SF3B1, SRSF2, ZRSR2, and U2AF1. Interestingly, these mutations occur in a mutually exclusive manner, as mutations in more than one factor prove lethal to both tumor cells and normal cells.^281^ Recent studies have discovered widespread abnormal ASE in hematological tumors, both with and without mutations affecting the AS mechanism. This suggests that changes in the expression levels of AS-related RBPs play a significant role in hematological tumors. A recent review summarized the regulatory role of abnormal RBPs in acute lymphoblastic leukemia (ALL) and acute myeloid leukemia (AML) with MLL gene rearrangements. In these cases, RBM39 can interact with SF3B1 and U2AF65, targeting HOXA9 and thus promoting intron retention.^282^ Another review has concluded that SRSF2 is mutated in more than 20% of MDS and 50% of chronic myeloid leukemia, causing expansion of the hematopoietic progenitor cell lineage, increased proliferation, apoptosis, and peripheral hematopoiesis.^283^ However, such comprehensive reviews are limited to other hematological tumors.

#### Acute myeloid leukemia (AML)

AML is the second most common type of leukemia in adults, and despite extensive basic and clinical research, its prognosis remains poor. It is an aggressive hematological tumor in which malignant myeloid precursor cells impair hematopoietic function and induce bone marrow failure.^284^ The t (8;21) chromosomal abnormality is the most common cytogenetic abnormality in AML patients. Patients with this such abnormality generally have a good prognosis, but still, 30–40% of patients relapse.^285^ The production of RUNX1/RUNX1T1 fusion oncogene plays a recognized role in regulating transcription and promoting cancer,^286,287^ such as directly controlling AS start site selection in target genes and directly or indirectly influencing the expression of genes encoding SFs. This results in numerous cancer-related ASEs that regulate nucleotide metabolism, cell adhesion, cell differentiation, and other leukemia-related processes.^288^ In AML genes with loss-of-function (LOF) mutations, AS significantly reduces the expression of many AML-related proteins, which is unrelated to somatic mutations recognized as driving AML occurrence.^289^ A transcriptome analysis of 982 AML patients revealed that IDH2 and SRSF2 mutations frequently overlap, promoting tumor occurrence through coordinated effects on the epigenome and RNA splicing. Mutations in IDH2 or SRSF2 bring about different splicing changes. IDH2 mutations change the splicing effects of mutated SRSF2 and lead to more profound ASEs than single mutations alone, as well as lethal myelodysplasia with proliferative characteristics in vivo and enhanced tumor self-renewal. IDH2 and SRSF2 double mutant cells have increased stalling of RNA polymerase II (RNAPII) due to abnormal AS and reduced expression of integrator complex 3 member (INTS3). Abnormal ASE of INTS3 promotes leukemia occurrence together with IDH2 mutation.^279^ In addition, SRSF1, another member of the SRSF family, relies on PRMT5 methylation to ensure its proper positioning and function. The deficiency in PRMT5 leads to changes in ASE among several crucial genes, the normal function of which is vital for AML cell survival.^290^ A comprehensive CRISPR/Cas9 domain screen targeting 490 classical RBPs uncovered the RBP dependence in human cancers.^291^ Importantly, RBM39 can inhibit cassette exon inclusion and promote intron retention in mRNAs encoding HOXA9 and other RBPs preferred by AML. The impact of RBM39 deficiency on AS further leads to lethal spliceosome mutant AML. Furthermore, abnormal ASEs in AML are associated with cohesin, a key element in chromatin organization and gene expression, previously thought to interact with chromatin,^292^ and AS-related RBPs interacting with cohesin mutants are frequently found in AML. Cohesin mutations are highly associated with different AS patterns and interact directly with BRD4 to produce an AS pattern different from any single factor acting alone.^293^ Given the important role of AS in AML, associating the global effects of AS on AML with prognosis is quite significant. Through analyzing AML patients in the National Taiwan University Hospital (NTUH) cohort and the TCGA database, ASEs of SYTL4, MYO9B, GFI1, and NPIPB4 are confirmed with independent prognostic significance,^294^ which greatly promotes the exploration of global ASEs in tumors and provides new ideas for risk stratification of patients. Wang et al.^295^ utilized CRISPR-Cas9 screening to discover that the combined application of BCL2 inhibitors and RBM10 inhibitors can result in the inactivation of the apoptosis inhibitor XIAP. Furthermore, the inhibition of CLKs and dual-specificity tyrosine-regulated kinases (DYRKs) can lead to abnormal ASEs in AS-related RBPs and apoptosis-related factors. This synergizes with venetoclax and helps overcome AML resistance to BCL2 inhibitors. This significant discovery underscores the considerable therapeutic potential of regulating abnormal ASEs in tumors.

#### Myelodysplastic syndrome (MDS)

Patients with MDS exhibit abnormal activation of toll-like receptors (TLR), which leads to a decrease in the self-renewal of hematopoietic stem cells, biased differentiation of bone marrow, and a reduction in neutrophils.^296^ TLR signaling plays a crucial role in regulating bone marrow hematopoiesis and innate immune responses. However, prolonged stimulation of TLR can result in hematopoietic stem and progenitor cell (HSPC) dysfunction.^297^ TRAF6, a TLR effector molecule with ubiquitin ligase activity, is significantly upregulated in MDS, ubiquitinating HNRNPA1 and thereby regulating the exon 2 exclusion of Arhgap1, which has been shown to regulate the self-renewal and differentiation of long-term hematopoietic stem cells.^298^ Furthermore, the activation of the Rho family GTP-binding protein, Cdc42 leads to hematopoietic defects in HSPCs expressing TRAF6.^299^

#### Lymphatic leukemia

In the progression of T lymphocytic leukemia (T-ALL), SRSF family member SRSF6 is regulated by ubiquitin-specific peptidase 7 (USP7) through deubiquitination. Inhibiting USP7 can change the exon skipping pattern and inhibit T-ALL growth. The splicing inhibitor H3B-8800 affects the splicing and activity of proteasome transcripts and synergizes with proteasome inhibitors to inhibit T-ALL growth.^300^ As previously mentioned, HNRNPH1 and PTBP1 jointly mediate the ASEs of transcription factor TCF3, which are vital for the fate of human embryonic stem cells.^166,167^ The team subsequently found that changes mapping to TCF3-Exon 18b were significantly enriched in MYC-driven B-cell lymphoma^301^ and revealed that G1663C and G1681A mutations could cause HNRNPH1 to lose its binding site, leading to a significant change in the ratio of TCF E12 and E47 isoforms and promoting tumor progression.^302^ Recently, numerous studies have focused on the regulation of AS and AS-related RBPs in tumor recurrence following CART-19 treatment in B lymphocytic leukemia (B-ALL) patients. After B lymphocytic leukemia targets CD19 treatment, the effect of CART-19 treatment often weakens,^303^ which is more common in adults.^304^ Abnormal ASEs of CD19 are one of the causes of CART-19 resistance.^305–307^ CD19 exon 2 skipping leads to truncated CD19 isoforms no longer located on the cell surface,^308^ while intron 2 retention^309^ and exons 5 and 6 skipping^308^ introduce PTCs that destroy CD19. Cortés-López et al.^310^ provided potential treatment measures for B-ALL patients resistant to CART-19. Through the integration of high-throughput mutagenesis and mathematical modeling, they identified more than 200 point mutations affecting CD19 ASEs and discovered that PTBP1, SRSF3, HNRNPM, HNBRNPK, and other RBPs have binding sites with CD19 exons 1-3. Loss-of-function RBPs can produce non-functional CD19 isoforms. Notably, the team found that knocking out the PTBP1 increases the CD19 intron 2 retention, thereby reducing the presentation of CD19 epitopes. Combined with previous reports that imatinib treatment can reduce PTBP1 expression,^311^ this suggests an important regulatory role for PTBP1 in the drug response in B-ALL.

Wang et al.^291^ employed CRISPR/Cas9 to screen several RBPs associated with AS that are closely linked to the progression of AML. Besides RBM39, which the team thoroughly investigated, it is crucial to conduct comprehensive research on other proteins in the future to clarify their molecular mechanisms in leukemia. This could potentially facilitate the advancement of clinical treatment strategies for AML and enhance survival rates for AML patients. MDS, a disease characterized by a poor prognosis and limited treatment options, could stand to benefit from targeting ASEs and modulating its abnormally activated downstream pathways, such as the RAS/MAPK pathway, using Proteolysis Targeting Chimeras (PROTACs). This approach may open new avenues for cancer treatment and prevention.

### Prostate cancer (PCa)

PCa is one of the leading causes of cancer-related deaths among men.^312^ While there have been comprehensive reviews on dysregulated ASEs and AS-related RBPs in prostate cancer,^313–315^ this section will focus on significant findings about PCa over the past 3 years.

The diversity of oncogenes or tumor suppressor factors recurrently affects the transcription and translation in tumor cells.^316^ FOXA recruits SFs to calibrate ASEs in PCa by analyzing transcriptomics, protein-mRNA interactions, epigenomics, and chromosome conformation. For example, FOXA binds to PTBP1, U2AF2, and HNRNPC at 3’ ss and HNRNPK at the upstream intron–exon boundary and downstream intron, thereby affecting patient survival.^317^ FOXA1 also regulates SRSF1 and causes downstream oncogene FLNA exon 30 inclusion to promote tumor growth.^318^ Besides, MYC is widely present in PCa and has a regulatory effect on ASEs.^319^ Numerous AS regulatory proteins are responsive to MYC expression levels, particularly in terms of cassette exon selection, suggesting that MYC signaling plays a crucial role in the regulation of AS, coupling NMD, and is a key component of growth control programs.^320^ The ultra-conservative NMD determinant exon of SRSF3 is notably sensitive to MYC signaling. MYC has been found to regulate the ASE of HRAS exon 5, a member of the Ras Oncogene family. Additionally, HNRNPH/F has been discovered to activate the AS of HRAS exon 5, thereby promoting the progression of the cell cycle and cell proliferation in PCa cells.^321^ PRMTs also contribute significant modifications to AS-related RBPs in PCa. PRMT4, PRMT5, and PRMT7 collectively regulate the arginine methylation of HNRNPA1. This protein is overexpressed in various cancers, including PCa, and is strongly associated with changes in cancer-related ASEs.^322^ Serine/arginine repetitive matrix protein 4 (SRRM4) has been found to regulate the RE1-silencing transcription factor (REST) in PCa. Specifically, SRM4 incorporates exon N3c into REST RNA, leading to the expression of a truncated isoform, REST4, which lacks the C-terminal transcriptional repression domain. As a result, neuronal genes are expressed, leading to a loss of REST inhibitory activity.^323^ Importantly, bone metastasis is a common event among PCa patients.^324^ Recent findings suggest that the cold-stress response protein, RBM3, which is highly expressed in prostate cancer cells, can interfere with the ASE of CD44 to weaken Pca cell stemness.^325^ Under normal conditions, RBM3 upregulates m6A modification in CTNNB1 3’UTR, reducing the stability of catenin beta 1 (CTNNB1) mRNA, leading to a decrease in β-catenin protein levels and downregulation of Wnt signal transduction. Consequently, the role of osteoblasts in remodeling PCa cell stemness is significantly weakened. The downregulation of RBM3 in PCa cells weakens this inhibitory effect.^326^ In addition to being regulated by RBM3, ASE of CD44 has also been reported to be regulated by PCBP1^327^ and TGF-β1.^328^ An increase in the expression of CD44 promotes EMT and upregulation of stem cell markers in PCa cells, thereby enhancing their invasive and tumorigenic abilities. The discovery of new biomarkers for PCa is currently a focal point.^329^ Recent findings suggest that the AS of the heterotrimeric transcription factor (TF) NF-Y could serve as a biomarker for further refinement of molecular subtypes among PCa patients.

The phenomenon of drug resistance and subsequent tumor progression in PCa patients has been confirmed to be associated with ASEs.^330^ Androgen deprivation therapy is currently the primary treatment for advanced PCa.^331^ During this therapy, AR expression increases, leading to the AS of AR and the production of AR-V7. The latter is resistant to interference by antiandrogen therapy. Targeting AR-V7 in combination with other treatments may have a significant therapeutic effect. This approach has been reported for its therapeutic effect.^332,333^ It may be a promising endeavor to treat prostate cancer patients by targeting AS-related RBPs to modulate the ASEs and thereby treat the patients.

### Colorectal cancer (CRC)

CRC is the third most common type of cancer and a significant contributor to cancer-related deaths worldwide.^334^ Given the prevalence of abnormal ASEs in tumors, research in the CRC field is particularly noteworthy.

CRC generally exists in a hypoxic environment.^335^ In this setting, PTBP1 binds with the hypoxia-induced lncRNA LUCAT1, leading to a series of pro-cancer ASEs related to downstream DNA damage genes. Concurrently, CRC cells develop resistance to DNA damage drugs, leading to metastasis,^336^ where PTBP1 also plays a role in regulating microexon abnormal AS in CRC. Specifically, PTBP1 and RBFOX2 can bind with pre-mRNA-containing microexons and regulate their AS. This process promotes tumor metastasis in CRC cells.^337^ SRSF2 is found to be highly expressed in CRC compared to normal tissues, and it significantly accelerates the proliferation of CRC cells both in vitro and in vivo. SRSF2 activates the alternative exon 24 inclusion of SLMAP by binding with constitutive exon 25. In addition, SRSF2 promotes the alternative exon 5 exclusion of CETN3 by binding to adjacent exon 6. This results in the production of SLMAP-L and CETN3-S AS variants, promoting CRC cell cycle progression.^338^ SRSF6 is also upregulated in CRC and associated with poor prognosis, promoting proliferation and metastasis. As a major regulator of AS in tight junction protein 1 (ZO-1), SRSF6 is translocated to the cell nucleus by TNPO3, causing ZO-1 exon 23 AS to exert its oncogene function. This can promote tumor proliferation, metastasis, and invasion.^339^ ZO-1 is also affected by Glioma tumor suppressor candidate region gene 1 (GLTSCR1), leading to a decrease in transcription elongation rate, and thus providing a time window for HuR to bind to specific motifs in ZO-1 intron 22 and spliceosome recognition of 3’ and 5’ splice sites in exon 23 to promote exon 23 inclusion.^340^ In addition, SRSF10 specifically binds and activates Bcl-2-associated transcription factor 1 (BCLAF1) exon 5a, which has been found to play a role in apoptosis signaling.^341^ SRSF10 induces the production of full-length BCLAF1 isoform (L-isoform) in CRC, thereby maintaining the oncogenic phenotype of CRC cells.^342^ SRSF11 is also upregulated in CRC and is phosphorylated by oncogenic kinase PAK5 to avoid ubiquitination degradation. Overexpressed SRSF11 directly binds with HSPA12A exon 2 to specifically regulate ASEs, increasing N-cadherin expression to exert a pro-cancer effect.^343^ The inhibitor SM08502, which targets SRSF kinase CLKs, is currently undergoing clinical trials (NCT03355066). In xenograft mouse models, oral administration of SM08502 can significantly inhibit the growth of gastrointestinal tumors, and reduce SRSF phosphorylation and Wnt pathway gene expression. The results of this study strongly suggest that intervening in abnormal ASEs in CRC has therapeutic potential.^344^

Recent studies have utilized AR-CLIP (photoactivatable ribonucleoside-enhanced cross-linking and immunoprecipitation) to discover that p53 can regulate ZMAT3 to control the ASE in CRC cells. ZMAT3 is a highly conserved RBP with tumor suppressor function.^345^ In CRC, p53, which has a high mutation frequency, has also been reported to regulate ZMAT3 to exert tumor suppressor function.^346,347^ Silencing ZMAT3 leads to the variant exon inclusion of CD44, increased expression of oncogenic long CD44 isoforms (CD44v), and silencing p53 results in the same outcome.^348^ In addition, an undefined RBP, Matrin3, was recently found to be involved in CDC14B ASEs. The latter is a key regulatory factor for mitotic spindle assembly, thereby exerting a function promoting tumor growth.^349^

### Pancreatic ductal adenocarcinoma (PDAC)

The prognosis of PDAC is extremely poor, largely due to the occurrence of metastasis at the time of diagnosis.

ESRP1, the first reported AS-related RBP in PDAC, promotes early metastasis. In PDAC, ESPR1 binds with ISE/ISS in the intron between FGFR2IIIb and IIIc exons, inhibiting PDAC metastasis. However, ESRP1 is significantly downregulated in PDAC, losing its tumor suppressor function.^350^ Besides, more than 90% of PDACs have KRAS mutations, primarily manifested as KRASG12D mutations, causing persistent activation of KRAS-related pathways.^351^ Pancreatitis will accelerate the progression of KRAS mutant PDAC.^352^ Wan et al.^353^ found that SRSF1 downregulation is a negative feedback response of cells to KRASG12D mutation. It inhibits MAPK signal activity and helps maintain the homeostasis of pancreatic cells. In addition, MYC in PDAC activates SRSF1^354^ to complete cytoplasmic-nuclear shuttling, regulating IL1R1 pre-mRNA 5’ UTR ASE in the nucleus to generate more stable mRNA isoforms and cause IL1R1 protein accumulation, which promotes the binding of IL1α/β secreted by epithelial cells and tumor matrix cells, feedback activates IL1 signal transduction^355^ and leads to activation of the MAPK pathway. The upregulation of SRSF1 and KRASG12D has a synergistic effect in tumorigenesis, so it is worth further study. In addition to KRAS mutations, SMAD4, CDKN2A, and TP53 are all drivers of PDAC, but no correlation has been found between them and PDAC progression to metastasis.^356^ Jbara et al.^357^ analyzed the AS characteristics of PDAC patients and pointed out that RBFOX2 may be of great significance in PDAC metastasis. Overexpression of RBFOX2 in patient-derived xenograft (PDX) metastatic PDAC cell lines can greatly reduce their metastatic potential, while removal of RBFOX2 in primary pancreatic tumor cell lines will increase the metastatic potential. RNA sequencing and splicing analysis of RBFOX2 target genes reveal that genes in the RHO GTPase pathway are enriched, indicating that RBFOX2’s splicing activity plays a role in cytoskeletal organization and lesion adhesion formation. In addition, HNRNPC a member of the HNRNP family, was found to be overexpressed in PDAC, promoting early tumor metastasis by antagonizing anti-metastatic alternative splicing isoform (TAF8L) and increasing pro-metastatic AS isoform (TAF8S).^358^

Another significant factor contributing to the poor prognosis of PDAC is resistance to chemotherapy regimens such as gemcitabine.^359^ Specifically, PTBP1 is significantly upregulated in drug-resistant PDAC cells and regulates PKM pre-mRNA AS, producing a large amount of PKM2 isoforms and causing gemcitabine resistance.^360^ CLK1, an upstream protein of SRSF family members, is significantly upregulated in PDAC. Its overexpression activates the phosphorylation of SRSF5 at serine 250, which promotes the proliferation, migration, and invasion of PDAC cells. The CLK1-SRSF5 axis formed thereby affects the ASEs of METTL14 and Cyclin L2, regulating PDAC cell m6A modification, cell migration, and invasion. In addition, DHX38 regulates intron 4 retention in receptors expressed in lymphoid tissue (Relt)-like 2 (RELL2) in gemcitabine-resistant PDAC cells. This has been shown to have an antitumor metastasis effect in BRCA.^361^ Overexpression of DHX38 can promote the normal splicing of RELL2 pre-mRNA and the synthesis of RELL2 protein, thereby inhibiting tumor progression.^362^ However, this study did not explore the specific mechanism, indicating a need for further research to explore the therapeutic potential of this target for PDAC. A recent study conducted a whole-genome sQTL analysis on 176 PDAC samples in TCGA, systematically identifying genetic variations controlling transcript isoforms - a total of 16175 sQTLs. By integrating a large population composed of 2782 PDAC patients and 7983 healthy controls and conducting a series of functional experiments, it was determined that an sQTL variant rs1785932 is significantly associated with a reduced risk of PDAC. It promotes the AS of ELP2 exon 6 to affect the expression levels between different isoforms of ELP2, thereby having different effects on phosphorylated STAT3 (pSTAT3) signal transduction and PDAC cell growth.^363^

### Hepatic cell cancer (HCC)

HCC is a major threat to human life and health, with most patients succumbing to tumor metastasis.^364,365^ Numerous studies have found that abnormal AS-related RBPs play a pro-cancer or anti-cancer role in HCC. However, the specific mechanism has not been elucidated.^366^ Recent research suggests that AS plays a pivotal role in tumor growth and metastasis.^367^ Furthermore, HCC often exhibits high tumor heterogeneity, which is associated with protein diversity caused by ASEs.^368^ Consequently, there is a focus on abnormal ASE and AS-related RBPs in HCC. While many AS-related RBPs have been found to play a key role, their regulatory role has not been fully elucidated in HCC. Lee et al.^369^ have reviewed RBPs regulating AS in HCC, so this paragraph focuses on summarizing the research progress of AS-related RBPs in HCC over the past 3–4 years.

A study employing single-molecule real-time long-read RNA sequencing technology to investigate global ASEs in HCC revealed the ASEs in HCC, including AS-related RBPs such as SRSF3, RBM27, MATR3, and YBX1. This study significantly advanced the understanding of ASEs in the field of HCC and holds great significance for identifying new treatment targets.^370^ The inactivation of SRSF2 in hepatocytes leads to acute liver failure and early death in mice.^371^ HBV infection can result in a decrease in SRSF2 expression, which promotes exon 3 inclusion in the proliferating cell nuclear antigen clamp-associated factor (PCLAF), leading to resistance to sorafenib, a common phenomenon among HCC patients.^372^ SRSF10, which is upregulated in HCC, regulates the exon 10 inclusion in SRSF12, thereby promoting the latter to regulate BLOC1S5-TXNDC5(B-T) expression, accelerating HCC tumor occurrence.^373^ SRSF2 is dephosphorylated by high levels of PPM1G (Protein phosphatase, Mg2 + /Mn2+ dependent 1 G) in liver cells, leading to a loss of SRSF3 tumor suppression. This induces abnormal AS of EMT-related genes and activates Wnt signal transduction and MYC activity.^374^ The dephosphorylation of SRSF3 induced by PPM1G further regulates the cell cycle and transcription regulation-related gene ASEs to promote HCC progression.^375^ Besides, SRSF3 directly binds with the coiled-coil domain-containing 50 (CCDC50) pre-mRNA, inducing its ASE and maintaining its stability in the cytoplasm. This further enhances HCC carcinogenesis through the Ras/Foxo4 signaling pathway.^376^ Moreover, SRSF10 promotes exon 6 exclusion in cell division cycle 25A (CDC25A) pre-mRNA, forming a stable CDC25A(△E6) isoform and producing a stronger cell cycle effect on HCC occurrence.^377^

Other RBPs also play regulatory roles in HCC. Shilo et al.^378^ found that HNRNPA2 regulates ASEs of A-Raf, increases the production of A-Raf in HCC cells, activates the Raf-MEK-ERK pathway, and promotes tumor cell proliferation. Zhou et al.^379^ have discovered that HNRNPAB is overexpressed in HCC and enhances HCC invasion and metastasis by regulating EMT-related factor SNAIL. Besides, HNRNPA2B1 in HCC is regulated by miRNA, which inhibits NF-κB pathway activation by regulating HNRNPA2B1 ubiquitination, thereby inhibiting tumor metastasis.^380^ Shen et al. ^381^ obtained the expression level of PTBP1 in HCC tissues using the TCGA database and cell lines. They found that PTBP1 activates Axl exon 10 exclusion to produce the Axl-S isoform. PTBP1 and Axl-S can stimulate cell migration and invasion in vitro, and promote the tumorigenicity and metastasis ability of liver cancer cells in vivo. In addition, PTBP1 is recruited by the highly expressed RNA helicase MTR4 in HCC, promoting cancer metabolic reprogramming in HCC. This includes pro-cancer abnormal ASEs of key glycolysis genes such as GLUT1 and PKM2.^382^ Liu et al.^383^ found that HNRNPC is significantly associated with various malignant characteristics of HCC, including tumor size, vascular invasion, tumor differentiation, and TNM staging. Knocking down HNRNPC can inhibit HIF1α mRNA stability and downregulate its expression level, thereby reducing HCC metastasis and invasion ability. HNRNPM in HCC is regulated by SOX2 and OCT4, leading to abnormal ASE of downstream Methyl-CpG binding protein 2 (MBD2). This produces a large amount of MBD2a (long isoform), which promotes FZD3 activation β-catenin by competitively binding to its CpG island, enhancing HCC cell stemness and promoting tumor progression.^384^ HNRNP RALY (also called heterogeneous nuclear ribonucleoprotein C-like 2) has been reported to be involved in regulating a large number of ASEs in HCC.^385^ It is found in the cholesterol synthesis pathway that RALY cooperates with SF3B3 to play a pro-tumor role in HCC, regulating Metastasis Associated 1 (MTA1) AS mode and leading to a decrease in MTA1-S isoform expression level. This reduces its inhibitory effect on cholesterol metabolism and cell proliferation.^386^

In the progression of HCC, intricate interactions transpire among AS-related RBPs. HCC patients undergoing radiotherapy exhibit aberrant PRMT5 ASEs, typified by exon 3 and a portion of exon 4 skipping, resulting in the truncated PRMT5-ISO5.^387^ SRSF3 and HNRNPH1 foster the selection of an alternative 3’ss on exon 4 over a 3’ ss on intron 2 by competitively binding with PRMT5 pre-mRNA, thereby antagonistically regulating PRMT5 ASE. Radiotherapy diminishes SRSF3 expression, leading to an elevation in PRMT5-ISO5 levels, which in turn enhances cell radiosensitivity and induces regression of xenograft tumors.^388^ NONO, DExH-box helicase 9 (DHX9), and splicing factor proline and glutamine-rich (SFPQ) interact to promote BIN1 exon 12a inclusion. These three proteins are frequently co-expressed in HCC, fostering HCC proliferation, migration, and tumor formation.^389^

A thorough investigation of AS-related RBPs in HCC holds immense significance for identifying early detection tumor markers and novel intervention targets. Future research could broaden its scope to the precancerous stage of HCC, potentially decelerating or obstructing disease progression before patients develop liver fibrosis or cirrhosis.

Increasing evidence indicates that AS significantly impacts the occurrence and development of various cancers. A survey of datasets from over 8000 patients with different types of cancer shows that the transcriptome diversity of tumors is more extensive compared to corresponding normal tissues.^390^ Research into AS and AS-related RBPs has also extended to other types of tumors. In patients with osteosarcoma, an observed increase in SRSF3 has been linked to a corresponding rise in the cell cycle regulators FoxM1, PLK1, and CDC25B. This upregulation enhances cell proliferation and transformation. Furthermore, SRSF3 has been found to facilitate the alternative splicing of isoforms 1 and 2 of the Interleukin Enhancer Binding Factor 3 (ILF3), which results in a loss of cell cycle regulation functions.^391,392^ In the context of neuroblastoma, the increased expression of HNRNPA1 and PTBP1 promotes the inclusion of PKM exon 10 in an ASE, thereby contributing to tumor progression.^393^

The widespread dysregulation of AS contributes to the complexity of cancers, and AS often produces carcinogenic mutations that promote tumor occurrence and drug resistance.^394,395^ However, the specific mechanisms remain largely elusive, necessitating future research to unravel the existing AS regulation, particularly investigations into oncogenic or tumor-suppressive ASEs and AS-related RBPs. On another note, AS abnormalities could potentially be a vulnerability in tumors that can be targeted for treatment. For instance, AS defects can generate tumor-specific novel junctions, encoding mutation-specific peptides or novel epitopes that could be harnessed for developing anti-cancer immunotherapies.^396^ Currently, research on AS and AS-related RBPs in the realm of tumors is primarily concentrated on the immune microenvironment of individual tumors. There is a need for a comprehensive analysis of the cumulative effects of ASEs leading to global TME infiltration characteristics across all types of cancer. Moreover, the complex relationship between AS and TME warrants further verification through experimental research. The processing mode of AS in pre-mRNA within TME and the molecular mechanism of how AS impacts TME are yet to be fully understood. Additionally, regulation upstream of AS-related RBPs also holds significance for tumor treatment. SRSF1, acting as a direct target of PRMT5, plays a crucial role in promoting cancer in AML. PRMT5 has been identified as an invasive tumor protein that promotes cell proliferation in various human tumors.^397–399^ In human AML and glioma cells, similarities in PRMT5 deletion-induced ASEs suggest common mechanisms underlying PRMT5 functions.^400^

### Non-neoplastic diseases

In addition to tumors, numerous ASE regulated by AS-related RBPs in non-neoplastic diseases have received widespread attention. Recent studies have provided a comprehensive overview of the role of RBPs in various kidney diseases. These include acute kidney injury (AKI), chronic kidney disease, renal fibrosis, polycystic kidney disease (PKD), diabetic nephropathy, and glomerulonephritis.^401^ Here we mainly summarize the important research progress in recent years. We hope to promote the research progress in related fields and provide ideas for disease diagnosis and treatment.

### Neurological diseases

Low et al.^402^ have conducted a detailed review of the regulatory role of HNRNP family members in neurodegenerative diseases, involving spinal muscular atrophy (SMA), Alzheimer’s disease (AD), amyotrophic lateral sclerosis (ALS), frontotemporal dementia (FTD), multiple sclerosis (MS), congenital muscular atrophy syndrome (CMS) and fragile X-related tremor/ataxia syndrome (FXTAS). They propose that the role of HNRNPs in neurological diseases is largely a neglected field, and more research may be a promising platform for developing new therapeutic targets.

#### Amyotrophic lateral sclerosis (ALS)

In the early stages of ALS, human induced pluripotent stem cell-derived motor neurons (MNs) are found that NOVA1 expression was significantly upregulated but functionally deficient, indicating that the RBP splicing network is disrupted in a complex manner early in the onset of ALS.^403^ As ALS progresses, NOVA1 levels gradually decrease, and RBP TAR DNA binding protein 43 (TDP43) gradually accumulates and functionally enhances.^404^ The loss of TDP43 in the nucleus and cytoplasmic accumulation is a characteristic of late-stage ALS. Excessive TDP43 increases its binding to the 3’ UTR of its mRNA, and splice normally silent 3’ UTR introns, producing another polyadenylation site downstream of the 3’ splice site. The proximity between the spliceosome assembled at this splice site and the polyadenylation complex causes destructive interactions, the splicing process is therefore terminated, and partially processed pre-mRNA is degraded, thereby regulating TDP43 expression. Under pathological conditions, TDP43 accumulates in neurons and consumes TDP43 protein expression through the above loop, mRNA levels significantly increase, leading to larger aggregates carrying more newly synthesized TDP43, leading to erroneous AS of genes involved in neuromuscular junctions,^405^ where MacNair et al.^406^ used translating ribosome affinity purification (TRAP) technology to find that MTHFSD and DDX58 regulated by TDP43 affect ALS-related neuroinflammation and stress granule pathways. In addition, HNRNPA2/B1 can regulate abnormal ASE of the ALS-related D-amino acid oxidase (DAO) gene, producing a large amount of DAO with serine metabolism function, while in ALS patients DAO pre-mRNA undergoes exon skipping to promote disease occurrence.^407^

#### Autism spectrum disorder (ASD)

ASD has shown comprehensive dysregulation of splicing networks. ASD patients have mutations in RBFOX, these mutations change the ASEs related to ASD, such as SH3 and multiple almond protein repeat domain protein 3(SHANK3), voltage-dependent L-type calcium channel subunit α1C(CACNA1C) and tuberous sclerosis 2 protein (TSC2, also known as tuberin). In addition, it is described that splicing networks of functionally related genes can affect “micro-exons” (3–15 nucleotides). In ASD patients, neuronal microexon inclusion is misregulated by upregulation of SRRM4. Therefore, compared with normal neurogenesis schemes, protein interaction networks during brain development in autism patients may be reshaped.

#### Alzheimer’s disease (AD)

AD is a chronic, late-onset neurodegenerative disorder, marked by the accumulation of amyloid plaques and neurofibrillary tangles, leading to memory impairment and cognitive decline.^408^ Tau, a microtubule-associated protein, plays a crucial role in axonal transport and neuronal growth. AS of exon 10 results in two isoforms of Tau with either three (3R) or four (4R) microtubule-binding repeat sequences. An imbalance in the 3R/4R ratio is linked to neurodegeneration as it affects the intracellular transport of the amyloid precursor protein (APP), which is associated with AD.^409^ In addition, SRSF1 and PTBP1 have been identified as splicing enhancers. They increase the level of mature CD33 mRNA, an exon 2 inclusion quantity that inhibits microglial phagocytosis of neurodegenerative plaques. This mechanism contributes to the late progression of AD.^410^ Furthermore, the long isoform of HNRNP D-like (L-DL), a member of the HNRNP family, can mediate the interaction between SF3B3 and U2AF65. Overexpression of L-DL can enhance the cognitive function of AD mice by regulating the AS and expression of the synaptic gene CAMKV.^411^ Recently, Tip60 histone acetyltransferase has been implicated in AD. It causes reduced histone acetylation in AD, leading to changes in chromatin packaging and transcriptional dysregulation in neurons. This has been found to induce abnormal ASE of AD-related mRNA and is considered a new pathological feature of AD.^412^

#### Huntington’s disease (HD)

HD is a devastating neurological disorder, marked by pronounced motor symptoms and significant striatal atrophy.^413^ research has discovered that the binding motifs of TIA1, U2AF2, HNRNPC, and PTBP are abundant in the upstream intronic sequences of HD-related pathogenic genes. Conversely, the binding motifs of RBFOX and ELAVL are prevalent in the downstream intronic sequences of these genes. This reveals the presence of pathogenic global aberrant ASEs in the striatum of HD.^414^ Mullari et al.^52^ analyzed brain tissue from the HD R6/2 mouse model and found differential binding of RBM5 with RNA. They found significant overlap between RBM5 and HD-related proteins, revealing its previously unknown role in neurodegeneration and HD progression.

### Autoimmune disease

AS plays a key role in the occurrence and development of various diseases, but it has not been clearly explained in autoimmune diseases, and research on related RBPs that cause abnormal ASEs is limited. SRSF1 dysfunction leads to systemic lupus erythematosus (SLE) and psoriasis. Li et al. have conducted a detailed review, SRSF1 expression reduction leads to its inability to bind CD3ζ 3’-UTR, reducing the expression of wild-type CD3ζ, and the latter’s expression deficiency is a characteristic of SLE. SRSF1 downregulation also leads to a decrease in RasGRP1 and IL2 expression in T cells of SLE patients. DDX5 downregulation leads to a decrease in SRSF1 expression, thereby leading to the sacrifice of IL-36R sIL-36R, and sIL-36R promotes the inflammatory response of psoriasis and atopic dermatitis.^415^ Recently, a study analyzed the composition of AS in peripheral blood and synovial fluid leukocytes in patients with rheumatoid arthritis (RA), revealing that eight elements SNRNP70, SNRNP200, U2AF2, RNU4ATAC, RBM3, RBM17, KHDRBS1, and SRSF10 are regulated by anti-citrullinated protein antibodies (ACPA) during the treatment process of RA patients and are reversed by anti-TNF therapy.^416^

The depiction of ASE in autoimmune diseases requires more in-depth research. Ren et al.^417^ have conducted a detailed review of abnormal ASEs in SLE, RA, and related therapeutic targets, and combined with previous views to summarize abnormal ASEs in autoimmune diseases. Gene mutations are the most common cause of AS.^418^ Second, AS-related RBPs regulated by epigenetic regulation will produce more different functions thereby affecting ASEs.^419^ Third, abnormal ASE of cell membrane antigens and nuclear antigens will lead to the production of new antigen epitopes, enhance the immunogenicity of existing antigens, or lead to the production of new autoantigens. The relationship between inflammation and autoimmune status in autoimmune diseases and how to restore their balance is key to treatment, which together urges us to further explore AS changes as targets as new tools for treating these autoimmune diseases.

### Endocrine system diseases and disorders

Diabetes and its complications are affecting the health of hundreds of millions of people worldwide, and the number of patients is increasing.^420,421^ With the widespread regulatory role of RBPs in physiological and pathological processes in AS, people have begun to pay attention to the role of RBPs leading to insulin resistance and diabetes. This part has been well-reviewed.^422–425^ Recently, Marcheva et al.^426^ reported that AS participates in the regulation of circadian rhythm in pancreatic β cells. thyroid hormone receptor-associated protein 3 (THRAP3) regulates circadian rhythm-dependent ASE by binding to exons flanking the coding sequence that is more often skipped in clock mutant β cells. These exons include transcripts encoding calcium/calmodulin-dependent serine protein kinase (Cask) and MAP kinase activating death domain (Madd), regulating sleep/wake cycle-dependent β cell function. Polycystic ovary syndrome (PCOS) is another common endocrine system disease, specifically manifested as cysts induced by endocrine disorders, irregular menstrual cycles, and even infertility.^427^ Studies have shown that high androgen levels and abnormal follicle formation in PCOS patients are significantly affected by two AS variants of the androgen receptor.^428^ Insulin-like growth factor-2 mRNA-binding protein 2 (IGF2BP2) can regulate a variety of variable splicing events in KGN cells.

### Digestive diseases

#### Non-alcoholic fatty liver disease (NAFLD)

NAFLD is one of the most common chronic liver diseases.^429^ Many studies have pointed out that AS and AS-related RBPs play an important role in the development of NAFLD. Del Río-Moreno et al.^430^ collected liver biopsy samples from 41 non-alcoholic obese patients undergoing weight loss surgery and found that the liver of obese and steatosis patients showed severe dysregulation of AS mechanism components. In vitro validation showed that silencing PTBP1, RBM45, and SND1 can reduce fat accumulation. Besides, SRSF3 is reported to be decreased in human liver samples with NAFLD, non-alcoholic steatohepatitis (NASH), or cirrhosis, leading to hepatic steatosis, fibrosis, and inflammation.^431^ In addition, HuR plays a key regulatory role in NAFLD by binding to Apob pre-mRNA intron 24, Uqcrb-3’UTR, and Ndufb6 mRNA 5’UTR, thereby regulating AS of Apob mRNA and UQCRB and NDUFB6 translation. Hepatocyte-specific HuR knockout will reduce the expression of APOB, UQCRB, and NDUFB6 in mice, thereby reducing liver lipid transport and ATP synthesis, exacerbating high-fat diet (HFD)-induced NAFLD.^432^ A recent study revealed^433^ that death-associated protein kinase-related apoptosis-inducing kinase 2 (DRAK2) directly binds to SRSF6, inhibits the phosphorylation of SRSF6 by SRSF kinase SRPK1, leading to abnormal AS isoforms of mitochondrial function-related genes (Polg2, Nudt13, Guf1, Rnasel and Nme4), promoting the progression of hepatic steatosis to non-alcoholic hepatitis. ESRP2 plays an important role in the progression of NAFLD to cholangiocarcinoma. As an important AS-related RBP, it can directly activate tumor suppressor factors to limit YAP/TAZ activation. In chronic inflammation progression, ESRP2 expression is inhibited, and inactive NF2 causes downstream YAP/TAZ activity to increase and promotes cholangiocarcinoma in chronic liver injury.^434^

#### Hepatitis B

The 3.5 kb pre-genome RNA (pgRNA) of the hepatitis B virus (HBV) can undergo AS. It encodes cap protein and polymerase protein and constitutes the template for viral genome replication. The ability of transcripts to undergo AS has been well studied in vitro and in patients with chronic hepatitis B infection (CHB).^435^ Recently, Duriez et al.^436^ found that 15% of proteins interacting with HBV pre-genome RNA are directly related to AS mechanisms. In chronic HBV carriers, HBV splicing-generated protein (HBSP) downregulates C-C motif chemokine ligand 2 (CCL2) expression in hepatocytes leading to immune evasion by HBV. Therefore, CCL2-targeted immunotherapy combined with nucleoside analogs should help effectively cure HBV by disrupting immune tolerance established during viral infection.

### Cardiovascular disease

The cardiovascular system is composed of various types of cells, regulating their phenotype to cope with acute or chronic injuries. Transcriptional and post-transcriptional mechanisms play a key role in regulating the remodeling and regeneration response of damaged cardiovascular tissues. At the same time, insufficient regulation of cell phenotype is closely related to the persistence and deterioration of cardiovascular diseases. Recently, RBPs such as QKIs, HuR, Muscleblind, and SRSF1 have become key regulatory factors for these functional adaptations in the cardiovascular system. They guide a lot of post-transcriptional events and have a significant impact on the fate of RNA (including alternative splicing, stability, location, and translation). This part has been quite extensively explained.^437^ Recent studies have found that RBM24 is a key AS regulatory molecule in dilated cardiomyopathy (DCM), its defects will lead to abnormal ASE of sarcomeric Z-disc complex, leading to early DCM, heart failure, and death in mice.^438^ RBP with multiple splicing (Rbpms), an uncharacterized RNA-binding factor is missing in Noncompaction cardiomyopathy, leading to defects in RNAs involved in the cytoskeleton signaling pathway, where the short isoform of heart-rich LIM domain protein Pdlim5 accumulates in large amounts, disrupting normal division of cardiomyocytes.^439^

## Conclusion and perspectives

**Fig. 5:**
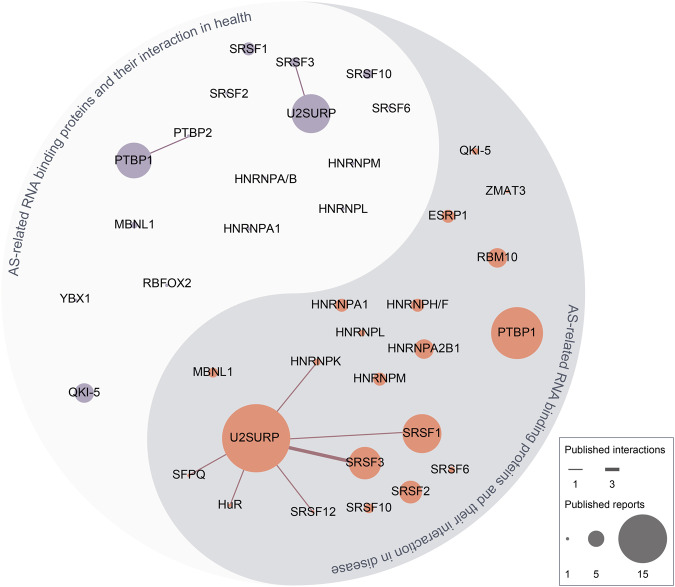
AS-related RBPs exhibit complex interrelationships in human health and disease based on the current research. The variable shear-related RNA-binding proteins addressed in this review, based on reported research, present complicated interactions throughout human health and disease. In particular, SRSF family members and HNRNP family members have received considerable attention. Remarkably, U2SURP has been reported to be involved in the variable shear regulatory network and play an important role by numerous studies in recent years. This figure was drawn by Adobe Illustrator

Recent advancements in sequencing technology have led to a new understanding of the role of AS-related RBPs, previously overlooked structures, in different tissues and cancers. These key molecules contribute to the formation of tissue or cell-type-specific AS products and protein isoforms that can either promote or inhibit cancer in tumor cells. AS-related RBPs exhibit complex interrelationships during the process and require further study to elucidate (Fig. 5).

This review encompasses a broad range of ASEs and AS-related RBPs, each playing distinct roles in various tissue development processes and disease states. It underscores the significant role of AS and AS-related RBPs in cellular plasticity and heterogeneity.^440,441^ Cellular plasticity is defined as the capacity of cells to modify their phenotype in response to environmental changes.^442^ In this context, AS is instrumental by generating diverse mRNA transcripts from a single gene, enabling cells to produce a variety of protein isoforms. This not only enhances the functional diversity of cells but also their adaptability to environmental changes. Regarding cellular heterogeneity, AS and RBPs make a substantial contribution. Varied splicing patterns can result in the production of distinct protein isoforms within different cells, even those within identical tissue types. This can give rise to cellular heterogeneity, a phenomenon frequently observed in a variety of biological processes, including development, tissue homeostasis, and disease progression. Gaining a deeper understanding of the intricate roles of AS in these processes can offer valuable insights into cellular function and disease progression, and potentially pave the way for the development of novel therapeutic strategies.

Recent years have seen a surge of novel findings in the field of AS-related RBPs. Among these, the significant role of poison exons in AS and AS-related RBPs has been highlighted. A poison exon is a unique type of exon typically included in the gene’s spliceosome, but it triggers NMD during translation, thereby inhibiting protein production. It can selectively splice pre-mRNA to yield different mRNAs and protein isoforms. This mechanism significantly broadens the proteome’s diversity, thereby enhancing the complexity of cellular functions, including cell differentiation and tumor development.^443^ The inclusion of poison exons is regulated by RBPs, and some of these proteins’ genes contain poison exons themselves, considered a self-regulation mechanism.^444^ Moreover, several recent studies have demonstrated that RBPs play a pivotal regulatory role in mRNA methylation modifications, including N6-methyladenosine (m^6^A) methylation, m^6^A demethylation, and N^7^-methylguanosine (m^7^G),^445^ further influencing tumor growth, tumor metabolism, and drug response. Concurrently, our understanding of the abnormal changes in the spliceosome’s composition for the role of AS is deepening. Disease-causing mutations in the driver genes that cause abnormal splicing of the entire transcriptome have been identified in various types of cancers. Most of these studies focus on protein-coding splicing factors, among which SF3B1 is the most extensively studied component.^446^ A recent study found that various pathogenic mutations in the SF3B1 gene can alter AS by disrupting the interaction with SUGP1. In the mutant spliceosome, the level of SUGP1 is reduced. Surprisingly, the knockdown of SUGP1 completely replicates the splicing errors, and the overexpression of SUGP1 drives the protein into the mutant spliceosome and partially restores the splicing.^447^ snRNA mutations associated with cancer have hardly been studied. The latest research reports a highly recurrent A > C somatic mutation on the third base of U1 snRNA in several tumors, changing the preferred A–U base pairing between U1 snRNA and the 5’ splice site to C-G base pairing, thereby creating new splice junctions and changing the splicing patterns of multiple genes.^448^ These latest research results are of great significance for a deeper understanding of the changes in AS-related RBPs and AS in physiological and pathological conditions. Targeting abnormal spliceosome composition and the self-regulatory mechanism of AS-related RBPs, or directly correcting the global abnormal ASEs caused by this, all have high research value. Recent studies have reported that spliceosome-targeted therapy can interfere with the function of the spliceosome in tumors, causing a large number of incorrectly spliced mRNAs to accumulate in the cytoplasm. Many of these mRNAs form double-stranded structures and are recognized by AS-related RBPs, thereby triggering antiviral signals and exogenous apoptosis.^449^ This is a new treatment method, targeting the common but heterogeneous abnormal AS characteristics among tumors, and changing the immune treatment response of tumor cells by recognizing abnormal AS patterns, is a potential universal intervention strategy.

In recent years, mRNA vaccines have emerged as a powerful tool in disease treatment, offering unique advantages such as safety, effectiveness, and ease of industrial production. Furthermore, AS has been recognized as a crucial source of new antigens for immunotherapy. This recognition opens promising avenues for research into targeted AS mechanisms in combination with immunotherapy. CAR-T-cell therapy, an innovative platform, holds potential for application in solid tumors with high local immune suppression. However, further research is required to identify antibodies that specifically recognize these targets and to develop and test CAR-T-cell reagents for treatment. Patient-derived organoids have gained considerable attention in recent years for their ability to capture the phenotypic and molecular heterogeneity of cancer and the process of tissue development. The exploration of ASE in organoid models is of significant value for understanding normal tissue development and tumor progression.

For future research, we propose an in-depth investigation into the following areas:

1. The integration of single-cell RNA-seq (scRNA-seq) and third-generation sequencing offers significant value in ASEs. ScRNA-seq provides the capability to capture the unique gene expression profile of each cell. Subsequently, third-generation sequencing could be employed to achieve longer, more precise read lengths, and to gather information about DNA modifications. The final step involves analyzing ASEs, which allows us to understand how a single gene can give rise to proteins with diverse functions. The combination of such information equips researchers with a comprehensive and profound understanding of the intricate gene network and regulatory mechanisms within an organism.^450,451^
2. Techniques such as Selective 2’-Hydroxyl Acylation Analyzed by Primer Extension, Mutational Profiling (SHAPE-MaP), and Parallel Analysis of RNA Structure (PARS) could be harnessed to uncover RNA structure-dependent interactions of RBP binding across the entire genome in living cells.^452–454^ This approach subsequently enables the exploration of the roles of RBP members with unclear AS regulatory mechanisms and the assessment of their impact on downstream abnormal gene expression.^455^
3. The application of protein transient degradation technology, including the newly discovered mandolin-proteasome,^456^ provides insights into the functional mechanisms of AS-related RBPs in ASEs. Moreover, the integration of this technique with transient transcriptome sequencing could potentially enhance our understanding of the mechanisms by which AS-related RBPs regulate transcription and prove beneficial in the development of new drugs.
4. At present, there are a limited number of drugs and clinical trials that target AS-related RBPs. Organoid technology, which can more accurately and rapidly simulate the in vivo environment, offers increased opportunities for disease research and drug target screening at both the animal and cellular levels.^457^
5. The advent and application of mRNA vaccines have marked a significant milestone in medical science. These vaccines carry mRNA molecules that, upon entering human cells, can modulate protein expression, which has proven effective in combating COVID-19, demonstrating notable antiviral properties.^458^ In the realm of AS regulation, there is potential for novel mRNA vaccines to be developed. These could control the expression of RBPs related to AS and rectify abnormal ASEs observed in various diseases. Such advancements could pave the way for innovative therapeutic strategies.^459^

Current research underscores the significance of AS as a crucial transcriptional regulatory mechanism. This process enables a single gene to generate a variety of mRNA isoforms, which in turn translate into proteins with diverse structures and functions. However, AS is not a static phenomenon. It varies across different tissues, stages of organ development and differentiation, and under various physiological or pathological conditions. A series of RBPs, specific to time and space, regulate ASEs to produce distinct products. This mechanism illuminates the intricate interplay between the micro and macro levels, matter and information, determinism, and diversity in life. Genes and proteins at the microscopic level influence the form and function of organisms at the macroscopic level through complex regulatory networks. Genes, as material entities, guide protein synthesis by encoding information. Deterministic gene sequences give rise to a diversity of proteins through RNA-binding protein-mediated alternative splicing. The complexity and diversity inherent in this process equip life with the ability to adapt to environmental changes and endow it with boundless possibilities. Delving deeper into this research will enhance our understanding of human health and disease.

## References

[1] Ule J, Blencowe BJ (2019). Alternative splicing regulatory networks: functions, mechanisms, and evolution. Mol. Cell.

[2] Khan MR, Wellinger RJ, Laurent B (2021). Exploring the alternative splicing of long noncoding RNAs. Trends Genet..

[3] Rogers SO (2019). Integrated evolution of ribosomal RNAs, introns, and intron nurseries. Genetica.

[4] Marasco LE, Kornblihtt AR (2023). The physiology of alternative splicing. Nat. Rev. Mol. Cell Biol..

[5] Huang ABZ, Delaidelli A, Sorensen PH (2020). RNA modifications in brain tumorigenesis. Acta Neuropathol. Com..

[6] Hoyos LE, Abdel-Wahab O (2018). Cancer-specific splicing changes and the potential for splicing-derived neoantigens. Cancer Cell.

[7] Wang S, Sun Z, Lei Z, Zhang HT (2022). RNA-binding proteins and cancer metastasis. Semin. Cancer Biol..

[8] Choi S, Cho N, Kim KK (2023). The implications of alternative pre-mRNA splicing in cell signal transduction. Exp. Mol. Med..

[9] Qin H (2020). RNA-binding proteins in tumor progression. J. Hematol. Oncol..

[10] Swanson M (2019). Dynamic mutations and RNA mis-splicing in disease. Eur. J. Hum. Genet..

[11] Scotti MM, Swanson MS (2016). RNA mis-splicing in disease. Nat. Rev. Genet..

[12] Saldi T, Riemondy K, Erickson B, Bentley DL (2021). Alternative RNA structures formed during transcription depend on elongation rate and modify RNA processing. Mol. Cell.

[13] Wilkinson ME, Charenton C, Nagai K (2020). RNA splicing by the spliceosome. Annu. Rev. Biochem..

[14] Van Nostrand EL (2020). A large-scale binding and functional map of human RNA-binding proteins. Nature.

[15] Van Nostrand EL (2016). Robust transcriptome-wide discovery of RNA-binding protein binding sites with enhanced CLIP (eCLIP). Nat. Methods.

[16] Jankowsky E, Harris ME (2015). Specificity and nonspecificity in RNA-protein interactions. Nat. Rev. Mol. Cell Biol..

[17] Erkelenz S (2013). Position-dependent splicing activation and repression by SR and hnRNP proteins rely on common mechanisms. Rna.

[18] Pereira B, Billaud M, Almeida R (2017). RNA-binding proteins in cancer: old players and new actors. Trends Cancer.

[19] Fica SM (2013). RNA catalyses nuclear pre-mRNA splicing. Nature.

[20] Wahl MC, Will CL, Luhrmann R (2009). The spliceosome: design principles of a dynamic RNP machine. Cell.

[21] Ying Y (2017). Splicing activation by Rbfox requires self-aggregation through its tyrosine-rich domain. Cell.

[22] Padgett RA (2012). New connections between splicing and human disease. Trends Genet..

[23] Wright CJ, Smith CWJ, Jiggins CD (2022). Alternative splicing as a source of phenotypic diversity. Nat. Rev. Genet..

[24] Wan R, Bai R, Zhan X, Shi Y (2020). How is precursor messenger RNA spliced by the spliceosome?. Annu. Rev. Biochem..

[25] Tian B, Manley JL (2017). Alternative polyadenylation of mRNA precursors. Nat. Rev. Mol. Cell Biol..

[26] Pal S, Gupta R, Davuluri RV (2012). Alternative transcription and alternative splicing in cancer. Pharmacol. Ther..

[27] Urbanski LM, Leclair N, Anczukow O (2018). Alternative-splicing defects in cancer: Splicing regulators and their downstream targets, guiding the way to novel cancer therapeutics. Wires Rna.

[28] Liu MM, Zack DJ (2013). Alternative splicing and retinal degeneration. Clin. Genet..

[29] Matera AG, Wang ZF (2014). A day in the life of the spliceosome (vol 15, pg 108, 2014). Nat. Rev. Mol. Cell Biol..

[30] Zhou HL, Luo G, Wise JA, Lou H (2014). Regulation of alternative splicing by local histone modifications: potential roles for RNA-guided mechanisms. Nucleic Acids Res..

[31] Do HTT, Shanak S, Barghash A, Helms V (2023). Differential exon usage of developmental genes is associated with deregulated epigenetic marks. Sci. Rep..

[32] El-Samad H (2021). Biological feedback control—respect the loops. Cell Syst..

[33] Pullmann R (2007). Analysis of turnover and translation regulatory RNA-binding protein expression through binding to cognate mRNAs. Mol. Cell Biol..

[34] Ding F (2022). Dynamics and functional roles of splicing factor autoregulation. Cell Rep..

[35] Zhou KI (2019). Regulation of co-transcriptional pre-mRNA splicing by m(6)A through the low-complexity protein hnRNPG. Mol. Cell.

[36] Zhang X (2017). An atomic structure of the human spliceosome. Cell.

[37] Bertram K (2017). Cryo-EM structure of a pre-catalytic human spliceosome primed for activation. Cell.

[38] Venables JP (2013). RBFOX2 is an important regulator of mesenchymal tissue-specific splicing in both normal and cancer tissues. Mol. Cell Biol..

[39] Brosseau JP (2014). Tumor microenvironment-associated modifications of alternative splicing. Rna.

[40] Lambert N (2014). RNA Bind-n-Seq: quantitative assessment of the sequence and structural binding specificity of RNA binding proteins. Mol. Cell.

[41] Jangi M, Boutz PL, Paul P, Sharp PA (2014). Rbfox2 controls autoregulation in RNA-binding protein networks. Genes Dev..

[42] Huang SC (2012). RBFOX2 promotes protein 4.1R exon 16 selection via U1 snRNP recruitment. Mol. Cell Biol..

[43] Gu M (2023). Suppression of RBFox2 by multiple MiRNAs in pressure overload-induced heart failure. Int. J. Mol. Sci..

[44] Damianov A (2016). Rbfox proteins regulate splicing as part of a large multiprotein complex LASR. Cell.

[45] Zhou D, Couture S, Scott MS, Abou Elela S (2021). RBFOX2 alters splicing outcome in distinct binding modes with multiple protein partners. Nucleic Acids Res..

[46] Castella S (2015). Ilf3 and NF90 functions in RNA biology. Wires Rna.

[47] Haque N (2018). ZFR coordinates crosstalk between RNA decay and transcription in innate immunity. Nat. Commun..

[48] Haque N, Will A, Cook AG, Hogg JR (2023). A network of DZF proteins controls alternative splicing regulation and fidelity. Nucleic Acids Res..

[49] Guo J, Jia J, Jia R (2015). PTBP1 and PTBP2 impaired autoregulation of SRSF3 in cancer cells. Sci. Rep..

[50] Hu X (2020). The RNA-binding protein AKAP8 suppresses tumor metastasis by antagonizing EMT-associated alternative splicing. Nat. Commun..

[51] Ye R (2023). Capture RIC-seq reveals positional rules of PTBP1-associated RNA loops in splicing regulation. Mol. Cell.

[52] Mullari M (2023). Characterising the RNA-binding protein atlas of the mammalian brain uncovers RBM5 misregulation in mouse models of Huntington’s disease. Nat. Commun..

[53] Lee FCY, Ule J (2018). Advances in CLIP technologies for studies of protein-RNA interactions. Mol. Cell.

[54] Kristofich J, Nicchitta CV (2023). Signal-noise metrics for RNA binding protein identification reveal broad spectrum protein-RNA interaction frequencies and dynamics. Nat. Commun..

[55] Hentze MW, Castello A, Schwarzl T, Preiss T (2018). A brave new world of RNA-binding proteins. Nat. Rev. Mol. Cell Biol..

[56] Vaquero-Garcia J (2023). RNA splicing analysis using heterogeneous and large RNA-seq datasets. Nat. Commun..

[57] Gonatopoulos-Pournatzis T (2018). Genome-wide CRISPR-Cas9 interrogation of splicing networks reveals a mechanism for recognition of autism-misregulated neuronal microexons. Mol. Cell.

[58] Wagner N (2023). Aberrant splicing prediction across human tissues. Nat. Genet..

[59] Ramanathan M, Porter DF, Khavari PA (2019). Methods to study RNA-protein interactions. Nat. Methods.

[60] Li YF (2021). CBRPP: a new RNA-centric method to study RNA-protein interactions. Rna Biol..

[61] Sciarrillo R (2020). The role of alternative splicing in cancer: from oncogenesis to drug resistance. Drug Resist. Update.

[62] Hu Y, Wang K, Li MY (2020). Detecting differential alternative splicing events in scRNA-seq with or without unique molecular identifiers. PLoS Comput. Biol..

[63] Wan Y, Larson DR (2018). Splicing heterogeneity: separating signal from noise. Genome Biol..

[64] Bao SY, Moakley DF, Zhang CL (2019). The splicing code goes deep. Cell.

[65] Jaganathan K (2019). Predicting splicing from primary sequence with deep learning. Cell.

[66] Baeza-Centurion P (2019). Combinatorial genetics reveals a scaling law for the effects of mutations on splicing. Cell.

[67] Chen H (2019). DIFFUSE: predicting isoform functions from sequences and expression profiles via deep learning. Bioinformatics.

[68] Nag S, Goswami B, Das Mandal S, Ray PS (2022). Cooperation and competition by RNA-binding proteins in cancer. Semin Cancer Biol..

[69] Qi T (2022). Genetic control of RNA splicing and its distinct role in complex trait variation. Nat. Genet..

[70] Fu XD, Ares M (2014). Context-dependent control of alternative splicing by RNA-binding proteins. Nat. Rev. Genet..

[71] Liu Z (2023). Manual correction of genome annotation improved alternative splicing identification of *Artemisia annua*. Planta.

[72] Kalsotra A (2008). A postnatal switch of CELF and MBNL proteins reprograms alternative splicing in the developing heart. Proc. Natl. Acad. Sci. USA.

[73] Yang JB (2018). CLOCK interacts with RANBP9 and is involved in alternative splicing in spermatogenesis. Gene.

[74] Cheng CY, Wong EW, Yan HH, Mruk DD (2010). Regulation of spermatogenesis in the microenvironment of the seminiferous epithelium: new insights and advances. Mol. Cell Endocrinol..

[75] Schmid R (2013). The splicing landscape is globally reprogrammed during male meiosis. Nucleic Acids Res..

[76] Chen Y (2018). Single-cell RNA-seq uncovers dynamic processes and critical regulators in mouse spermatogenesis. Cell Res..

[77] Hannigan MM, Zagore LL, Licatalosi DD (2017). Ptbp2 controls an alternative splicing network required for cell communication during spermatogenesis. Cell Rep..

[78] Feng SL (2022). hnRNPH1 recruits PTBP2 and SRSF3 to modulate alternative splicing in germ cells. Nat. Commun..

[79] Qin J (2023). Bud31-mediated alternative splicing is required for spermatogonial stem cell self-renewal and differentiation. Cell Death Differ..

[80] Liu W (2022). SRSF10 is essential for progenitor spermatogonia expansion by regulating alternative splicing. eLife.

[81] Legrand JMD (2019). DDX5 plays essential transcriptional and post-transcriptional roles in the maintenance and function of spermatogonia. Nat. Commun..

[82] Sada A, Suzuki A, Suzuki H, Saga Y (2009). The RNA-binding protein NANOS2 is required to maintain murine spermatogonial stem cells. Science.

[83] Soni K (2023). Structural basis for specific RNA recognition by the alternative splicing factor RBM5. Nat. Commun..

[84] Song H, Wang L, Chen D, Li F (2020). The function of pre-mRNA alternative splicing in mammal spermatogenesis. Int. J. Biol. Sci..

[85] Gao Y (2018). Identification and characterization of circular RNAs in Qinchuan cattle testis. R. Soc. Open Sci..

[86] Zhou F, Chen W, Jiang Y, He Z (2019). Regulation of long non-coding RNAs and circular RNAs in spermatogonial stem cells. Reproduction.

[87] Schieweck R, Ninkovic J, Kiebler MA (2021). Rna-binding proteins balance brain function in health and disease. Physiol. Rev..

[88] Nikom D, Zheng S (2023). Alternative splicing in neurodegenerative disease and the promise of RNA therapies. Nat. Rev. Neurosci..

[89] Salehi S (2023). Cytosolic Ptbp2 modulates axon growth in motoneurons through axonal localization and translation of Hnrnpr. Nat. Commun..

[90] Licatalosi DD (2012). Ptbp2 represses adult-specific splicing to regulate the generation of neuronal precursors in the embryonic brain. Genes Dev..

[91] Zhang X (2016). Cell-type-specific alternative splicing governs cell fate in the developing cerebral cortex. Cell.

[92] Hayakawa-Yano Y, Yano M (2019). An RNA switch of a large exon of ninein is regulated by the neural stem cell specific-RNA binding protein, Qki5. Int. J. Mol. Sci..

[93] Quesnel-Vallieres M, Irimia M, Cordes SP, Blencowe BJ (2015). Essential roles for the splicing regulator nSR100/SRRM4 during nervous system development. Gene Dev..

[94] Zheng S (2020). Alternative splicing programming of axon formation. Wiley Interdiscip. Rev. RNA.

[95] Kim JH (2021). SON drives oncogenic RNA splicing in glioblastoma by regulating PTBP1/PTBP2 switching and RBFOX2 activity. Nat. Commun..

[96] Vuong JK (2016). PTBP1 and PTBP2 serve both specific and redundant functions in neuronal pre-mRNA splicing. Cell Rep..

[97] Chan JN (2022). RNA-binding protein signaling in adult neurogenesis. Front. Cell Dev. Biol..

[98] Qian H (2020). Reversing a model of Parkinson’s disease with in situ converted nigral neurons. Nature.

[99] Chen W (2022). Repressing PTBP1 fails to convert reactive astrocytes to dopaminergic neurons in a 6-hydroxydopamine mouse model of Parkinson’s disease. eLife.

[100] Hoang T (2022). Genetic loss of function of Ptbp1 does not induce glia-to-neuron conversion in retina. Cell Rep..

[101] Villate O (2014). Nova1 is a master regulator of alternative splicing in pancreatic beta cells. Nucleic Acids Res..

[102] Lin JC (2013). RBM4 promotes pancreas cell differentiation and insulin expression. Mol. Cell Biol..

[103] Du X (2023). Downregulated liver-elevated long intergenic noncoding RNA (LINC02428) is a tumor suppressor that blocks KDM5B/IGF2BP1 positive feedback loop in hepatocellular carcinoma. Cell Death Dis..

[104] Suzuki T (2020). Regulation of fetal genes by transitions among RNA-binding proteins during liver development. Int. J. Mol. Sci..

[105] Mobin MB (2016). The RNA-binding protein vigilin regulates VLDL secretion through modulation of Apob mRNA translation. Nat. Commun..

[106] Paterson HAB (2022). Liver RBFOX2 regulates cholesterol homeostasis via Scarb1 alternative splicing in mice. Nat. Metab..

[107] Vatandaslar H (2023). In vivo PAR-CLIP (viP-CLIP) of liver TIAL1 unveils targets regulating cholesterol synthesis and secretion. Nat. Commun..

[108] Osma-Garcia IC (2022). The splicing regulators TIA1 and TIAL1 are required for the expression of the DNA damage repair machinery during B cell lymphopoiesis. Cell Rep..

[109] Arif W (2023). Splicing factor SRSF1 deficiency in the liver triggers NASH-like pathology and cell death. Nat. Commun..

[110] Sen S, Jumaa H, Webster NJG (2013). Splicing factor SRSF3 is crucial for hepatocyte differentiation and metabolic function. Nat. Commun..

[111] Pihlajamaki J (2011). Expression of the splicing factor gene SFRS10 is reduced in human obesity and contributes to enhanced lipogenesis. Cell Metab..

[112] Bhate A (2015). ESRP2 controls an adult splicing programme in hepatocytes to support postnatal liver maturation. Nat. Commun..

[113] Bangru S (2018). Alternative splicing rewires Hippo signaling pathway in hepatocytes to promote liver regeneration. Nat. Struct. Mol. Biol..

[114] Hyun J (2020). Epithelial splicing regulatory protein 2-mediated alternative splicing reprograms hepatocytes in severe alcoholic hepatitis. J. Clin. Investig..

[115] Patel C (2015). Fructose-induced increases in expression of intestinal fructolytic and gluconeogenic genes are regulated by GLUT5 and KHK. Am. J. Physiol.-Reg. Integr. Comp. Physiol..

[116] Possik E (2021). New mammalian glycerol-3-phosphate phosphatase: role in beta-cell, liver and adipocyte metabolism. Front. Endocrinol..

[117] Mirtschink P, Jang C, Arany Z, Krek W (2018). Fructose metabolism, cardiometabolic risk, and the epidemic of coronary artery disease. Eur. Heart J..

[118] Li XJ (2016). A splicing switch from ketohexokinase-C to ketohexokinase-A drives hepatocellular carcinoma formation. Nat. Cell Biol..

[119] Pecori, R., Di Giorgio, S., Paulo Lorenzo, J. & Nina Papavasiliou, F. Functions and consequences of AID/APOBEC-mediated DNA and RNA deamination. *Nat. Rev. Genet.* **23**, 505–518 (2022). 10.1038/s41576-022-00459-8 PMC8900473 35256818

[120] Nikolaou KC (2019). The RNA-binding protein A1CF regulates hepatic fructose and glycerol metabolism via alternative RNA splicing. Cell Rep..

[121] Chembazhi UV (2023). PTBP1 controls intestinal epithelial regeneration through post-transcriptional regulation of gene expression. Nucleic Acids Res..

[122] Jin ZG, Liang F, Yang J, Mei WY (2017). hnRNP I regulates neonatal immune adaptation and prevents colitis and colorectal cancer. PLoS Genet..

[123] Ergun A (2013). Differential splicing across immune system lineages. Proc. Natl. Acad. Sci. USA.

[124] Alexander DR (2000). The CD45 tyrosine phosphatase: a positive and negative regulator of immune cell function. Semin. Immunol..

[125] Yabas M, Elliott H, Hoyne GF (2015). The role of alternative splicing in the control of immune homeostasis and cellular differentiation. Int. J. Mol. Sci..

[126] Chang X (2016). RNA-binding protein hnRNPLL as a critical regulator of lymphocyte homeostasis and differentiation. Wiley Interdiscip. Rev. Rna..

[127] Liao KC, Garcia-Blanco MA (2021). Role of alternative splicing in regulating host response to viral infection. Cells.

[128] Liu J, Cao X (2023). RBP-RNA interactions in the control of autoimmunity and autoinflammation. Cell Res..

[129] Geng G (2021). PTBP1 is necessary for dendritic cells to regulate T-cell homeostasis and antitumour immunity. Immunology.

[130] Lau CI (2021). The pioneer transcription factors Foxa1 and Foxa2 regulate alternative RNA splicing during thymocyte positive selection. Development.

[131] Cassidy MF, Herbert ZT, Moulton VR (2022). Splicing factor SRSF1 controls distinct molecular programs in regulatory and effector T cells implicated in systemic autoimmune disease. Mol. Immunol..

[132] Victora GD, Nussenzweig MC (2022). Germinal centers. Annu. Rev. Immunol..

[133] Monzon-Casanova E (2020). Polypyrimidine tract-binding proteins are essential for B cell development. eLife.

[134] Monzon-Casanova E (2018). The RNA-binding protein PTBP1 is necessary for B cell selection in germinal centers. Nat. Immunol..

[135] Osma-Garcia IC (2021). The RNA-binding protein HuR is required for maintenance of the germinal centre response. Nat. Commun..

[136] Diaz-Munoz MD (2015). The RNA-binding protein HuR is essential for the B cell antibody response. Nat. Immunol..

[137] Osma-Garcia IC (2023). The RNA binding proteins TIA1 and TIAL1 promote Mcl1 mRNA translation to protect germinal center responses from apoptosis. Cell Mol. Immunol..

[138] Sidali A (2021). AU-rich element RNA binding proteins: at the crossroads of post-transcriptional regulation and genome integrity. Int. J. Mol. Sci..

[139] Vikstrom I (2010). Mcl-1 is essential for germinal center formation and B cell memory. Science.

[140] Wagner AR (2022). SRSF6 balances mitochondrial-driven innate immune outcomes through alternative splicing of BAX. eLife.

[141] de Bruin RG (2016). Quaking promotes monocyte differentiation into pro-atherogenic macrophages by controlling pre-mRNA splicing and gene expression. Nat. Commun..

[142] Chen X (2021). The emerging roles of the RNA binding protein QKI in cardiovascular development and function. Front. Cell Dev. Biol..

[143] Giampietro C (2015). The alternative splicing factor Nova2 regulates vascular development and lumen formation. Nat. Commun..

[144] Angiolini F (2019). A novel L1CAM isoform with angiogenic activity generated by NOVA2-mediated alternative splicing. eLife.

[145] Belloni E (2019). Gene expression profiles controlled by the alternative splicing factor Nova2 in endothelial cells. Cells.

[146] Baek S (2019). The alternative splicing regulator Nova2 constrains vascular Erk signaling to limit specification of the lymphatic lineage. Dev. Cell.

[147] Chen HZ, Tsai SY, Leone G (2009). Emerging roles of E2Fs in cancer: an exit from cell cycle control. Nat. Rev. Cancer.

[148] Pradella D (2021). A ligand-insensitive UNC5B splicing isoform regulates angiogenesis by promoting apoptosis. Nat. Commun..

[149] Luo Y (2022). KRAS mutant-driven SUMOylation controls extracellular vesicle transmission to trigger lymphangiogenesis in pancreatic cancer. J. Clin. Investig..

[150] Xia XD (2017). Myocardin: a novel player in atherosclerosis. Atherosclerosis.

[151] van der Veer EP (2013). Quaking, an RNA-binding protein, is a critical regulator of vascular smooth muscle cell phenotype. Circ. Res..

[152] McCann KL, Baserga SJ (2013). Genetics. Mysterious ribosomopathies. Science.

[153] Hou VC (2002). Decrease in hnRNP A/B expression during erythropoiesis mediates a pre-mRNA splicing switch. EMBO J..

[154] Huang SC (2017). Protein 4.1R exon 16 3’ splice site activation requires coordination among TIA1, Pcbp1, and RBM39 during terminal erythropoiesis. Mol. Cell Biol..

[155] Alvarez-Dominguez JR, Zhang X, Hu W (2017). Widespread and dynamic translational control of red blood cell development. Blood.

[156] van den Hoogenhof MM, Pinto YM, Creemers EE (2016). RNA splicing: regulation and dysregulation in the heart. Circ. Res..

[157] Fochi S (2020). The emerging role of the RBM20 and PTBP1 ribonucleoproteins in heart development and cardiovascular diseases. Genes.

[158] Liu H (2023). Endothelial deletion of PTBP1 disrupts ventricular chamber development. Nat. Commun..

[159] Lu SH (2022). Alternative splicing mediated by RNA-binding protein RBM24 facilitates cardiac myofibrillogenesis in a differentiation stage-specific manner. Circ. Res..

[160] Akerberg AA (2022). RBPMS2 is a myocardial-enriched splicing regulator required for cardiac function. Circ. Res..

[161] Chen X (2021). QKI is a critical pre-mRNA alternative splicing regulator of cardiac myofibrillogenesis and contractile function. Nat. Commun..

[162] Zhao Y (2022). Cardiomyocyte-specific long noncoding RNA regulates alternative splicing of the triadin gene in the heart. Circulation.

[163] Farina NH (2012). A role for RNA post-transcriptional regulation in satellite cell activation. Skelet. Muscle.

[164] Bowman TV (2006). Differential mRNA processing in hematopoietic stem cells. Stem Cells.

[165] Park JW, Fu S, Huang B, Xu RH (2020). Alternative splicing in mesenchymal stem cell differentiation. Stem Cells.

[166] Yamazaki T (2018). TCF3 alternative splicing controlled by hnRNP H/F regulates E-cadherin expression and hESC pluripotency. Genes Dev..

[167] Yamazaki T, Liu L, Manley JL (2019). TCF3 mutually exclusive alternative splicing is controlled by long-range cooperative actions between hnRNPH1 and PTBP1. Rna.

[168] de Morree A, Rando TA (2023). Regulation of adult stem cell quiescence and its functions in the maintenance of tissue integrity. Nat. Rev. Mol. Cell Biol..

[169] Hayakawa-Yano Y (2017). An RNA-binding protein, Qki5, regulates embryonic neural stem cells through pre-mRNA processing in cell adhesion signaling. Gene Dev..

[170] Chen XY (2021). QKI is a critical pre-mRNA alternative splicing regulator of cardiac myofibrillogenesis and contractile function. Nat. Commun..

[171] Neumann DP, Goodall GJ, Gregory PA (2022). The Quaking RNA-binding proteins as regulators of cell differentiation. Wiley Interdiscip. Rev. RNA.

[172] Yang J (2014). RBM24 is a major regulator of muscle-specific alternative splicing. Dev. Cell.

[173] Fumagalli S, Totty NF, Hsuan JJ, Courtneidge SA (1994). A target for Src in mitosis. Nature.

[174] Rekad Z (2023). Coalescent RNA-localizing and transcriptional activities of SAM68 modulate adhesion and subendothelial basement membrane assembly. eLife.

[175] Song J, Richard S (2015). Sam68 regulates S6K1 alternative splicing during adipogenesis. Mol. Cell Biol..

[176] Chao Y (2021). Regulatory roles and mechanisms of alternative RNA splicing in adipogenesis and human metabolic health. Cell Biosci..

[177] Zhang P (2022). RNA-binding proteins in the regulation of adipogenesis and adipose function. Cells.

[178] Peng L (2023). New insights into transcriptome variation during cattle adipocyte adipogenesis by direct RNA sequencing. iScience.

[179] Fidalgo MF (2022). Aerocyte specification and lung adaptation to breathing is dependent on alternative splicing changes. Life Sci. Alliance.

[180] Wiegel, D., Dammann, C. E. L. & Nielsen, H. C. ErbB4 alternative splicing mediates fetal mouse alveolar type II cell differentiation in vitro. *Pediatr. Res*. (2022). 10.1038/s41390-022-02013-y PMC9509489 35338350

[181] Kedzierska H, Piekielko-Witkowska A (2017). Splicing factors of SR and hnRNP families as regulators of apoptosis in cancer. Cancer Lett..

[182] Xu H (2022). Emerging roles of hnRNP A2B1 in cancer and inflammation. Int. J. Biol. Macromol..

[183] Zhu W (2020). Roles of PTBP1 in alternative splicing, glycolysis, and oncogensis. J. Zhejiang Univ. Sci. B.

[184] Gallardo M (2016). Aberrant hnRNP K expression: all roads lead to cancer. Cell Cycle.

[185] Cerasuolo A, Buonaguro L, Buonaguro FM, Tornesello ML (2020). The role of RNA splicing factors in cancer: regulation of viral and human gene expression in human papillomavirus-related cervical cancer. Front. Cell Dev. Biol..

[186] Obeng EA, Stewart C, Abdel-Wahab O (2019). Altered RNA processing in cancer pathogenesis and therapy. Cancer Discov..

[187] McCutcheon IE (2004). Expression of the splicing regulator polypyrimidine tract-binding protein in normal and neoplastic brain. Neuro Oncol..

[188] Manke MC (2021). ANXA7 regulates platelet lipid metabolism and Ca(2+) release in arterial thrombosis. Circ. Res..

[189] Ferrarese R (2014). Lineage-specific splicing of a brain-enriched alternative exon promotes glioblastoma progression. J. Clin. Investig..

[190] Tomas A, Futter CE, Eden ER (2014). EGF receptor trafficking: consequences for signaling and cancer. Trends Cell Biol..

[191] White ZB (2019). Impact of ANXA7 I1 expression on PDGFRA and MET endosomal trafficking in glioblastoma. Int. J. Radiat. Oncol..

[192] Rao JH (2020). Nogo-B is a key mediator of hepatic ischemia and reperfusion injury. Redox Biol..

[193] Mashtalir N (2020). A structural model of the endogenous human BAF complex informs disease mechanisms. Cell.

[194] Sokpor G, Xie YB, Rosenbusch J, Tuoc T (2017). Chromatin remodeling BAF (SWI/SNF) complexes in neural development and disorders. Front. Mol. Neurosci..

[195] Aldave G (2018). The aberrant splicing of BAF45d links splicing regulation and transcription in glioblastoma. Neuro Oncol..

[196] Moroni RF (2006). Distinct expression pattern of microtubule-associated protein/microtubule affinity-regulating kinase 4 in differentiated neurons. Neuroscience.

[197] Rovina D (2014). Microtubule-associated protein/microtubule affinity-regulating kinase 4 (MARK4) plays a role in cell cycle progression and cytoskeletal dynamics. Eur. J. Cell Biol..

[198] Wang K (2022). PTBP1 knockdown promotes neural differentiation of glioblastoma cells through UNC5B receptor. Theranostics.

[199] Golan-Gerstl R (2011). Splicing factor hnRNP A2/B1 regulates tumor suppressor gene splicing and is an oncogenic driver in glioblastoma. Cancer Res..

[200] Deng JM (2016). Effects of hnRNP A2/B1 knockdown on inhibition of glioblastoma cell invasion, growth and survival. Mol. Neurobiol..

[201] Yin DC, Kong CX, Chen MH (2020). Effect of hnRNPA2/B1 on the proliferation and apoptosis of glioma U251 cells via the regulation of AKT and STAT3 pathways. Biosci. Rep..

[202] Fabian C, Han M, Bjerkvig R, Niclou SP (2021). Novel facets of glioma invasion. Int. Rev. Cell Mol. Biol..

[203] Decorsiere A, Cayrel A, Vagner S, Millevoi S (2011). Essential role for the interaction between hnRNP H/F and a G quadruplex in maintaining p53 pre-mRNA 3’-end processing and function during DNA damage. Genes Dev..

[204] Lefave CV (2011). Splicing factor hnRNPH drives an oncogenic splicing switch in gliomas. EMBO J..

[205] Le Bras M (2022). Translational regulation by hnRNP H/F is essential for the proliferation and survival of glioblastoma. Cancers.

[206] Zhou X (2019). Splicing factor SRSF1 promotes gliomagenesis via oncogenic splice-switching of MYO1B. J. Clin. Investig..

[207] Liu H (2021). SRPK1/2 and PP1alpha exert opposite functions by modulating SRSF1-guided MKNK2 alternative splicing in colon adenocarcinoma. J. Exp. Clin. Cancer Res..

[208] Maimon A (2014). Mnk2 alternative splicing modulates the p38-MAPK pathway and impacts Ras-induced transformation. Cell Rep..

[209] Mogilevsky M (2018). Modulation of MKNK2 alternative splicing by splice-switching oligonucleotides as a novel approach for glioblastoma treatment. Nucleic Acids Res..

[210] Song X (2019). SRSF3-regulated RNA alternative splicing promotes glioblastoma tumorigenicity by affecting multiple cellular processes. Cancer Res..

[211] Vikhreva P, Melino G, Amelio I (2018). p73 alternative splicing: exploring a biological role for the C-terminal isoforms. J. Mol. Biol..

[212] Fuentes-Fayos AC (2020). Splicing machinery dysregulation drives glioblastoma development/aggressiveness: oncogenic role of SRSF3. Brain.

[213] Morana O, Wood W, Gregory CD (2022). The apoptosis paradox in cancer. Int. J. Mol. Sci..

[214] Bugg D (2022). MBNL1 drives dynamic transitions between fibroblasts and myofibroblasts in cardiac wound healing. Cell Stem Cell.

[215] Fernandez-Costa JM, Llamusi MB, Garcia-Lopez A, Artero R (2011). Alternative splicing regulation by muscleblind proteins: from development to disease. Biol. Rev..

[216] Gates DP, Coonrod LA, Berglund JA (2011). Autoregulated splicing of muscleblind-like 1 (MBNL1) Pre-mRNA. J. Biol. Chem..

[217] Konieczny P (2017). Autoregulation of MBNL1 function by exon 1 exclusion from MBNL1 transcript. Nucleic Acids Res..

[218] Voss DM (2020). The alternative splicing factor, MBNL1, inhibits glioblastoma tumor initiation and progression by reducing hypoxia-induced stemness. Cancer Res..

[219] Xie Y (2016). Ferroptosis: process and function. Cell Death Differ..

[220] Sun S (2022). RNA binding protein NKAP protects glioblastoma cells from ferroptosis by promoting SLC7A11 mRNA splicing in an m(6)A-dependent manner. Cell Death Dis..

[221] Wang X (2022). Targeting the splicing factor NONO inhibits GBM progression through GPX1 intron retention. Theranostics.

[222] Zhang M (2019). Axonogenesis is coordinated by neuron-specific alternative splicing programming and splicing regulator PTBP2. Neuron.

[223] Zhao L (2020). Comprehensive characterization of alternative mRNA splicing events in glioblastoma: implications for prognosis, molecular subtypes, and immune microenvironment remodeling. Front. Oncol..

[224] Szklener K (2022). New directions in the therapy of glioblastoma. Cancers.

[225] Siegel RL, Miller KD, Fuchs HE, Jemal A (2021). Cancer statistics, 2021. CA Cancer J. Clin..

[226] Harbeck N, Gnant M (2017). Breast cancer. Lancet.

[227] Yu S (2020). Comprehensive analysis and establishment of a prediction model of alternative splicing events reveal the prognostic predictor and immune microenvironment signatures in triple negative breast cancer. J. Transl. Med..

[228] Yang Q (2019). Aberrant alternative splicing in breast cancer. J. Mol. Cell Biol..

[229] Liu Z (2021). Hypoxia-induced suppression of alternative splicing of MBD2 promotes breast cancer metastasis via activation of FZD1. Cancer Res..

[230] Gao FY (2023). c-MYC mediates the crosstalk between breast cancer cells and tumor microenvironment. Cell Commun. Signal..

[231] Du JX (2021). Splicing factor SRSF1 promotes breast cancer progression via oncogenic splice switching of PTPMT1. J. Exp. Clin. Cancer Res..

[232] Deng L (2023). MYC-driven U2SURP regulates alternative splicing of SAT1 to promote triple-negative breast cancer progression. Cancer Lett..

[233] Yu Y, Fang L (2022). CircRPAP2 regulates the alternative splicing of PTK2 by binding to SRSF1 in breast cancer. Cell Death Discov..

[234] Wang S (2019). High-throughput chemical screening identifies focal adhesion kinase and aurora kinase B inhibition as a synergistic treatment combination in Ewing sarcoma. Clin. Cancer Res..

[235] Fan T (2016). Bit1 knockdown contributes to growth suppression as well as the decreases of migration and invasion abilities in esophageal squamous cell carcinoma via suppressing FAK-paxillin pathway. Mol. Cancer.

[236] Fish L (2021). A prometastatic splicing program regulated by SNRPA1 interactions with structured RNA elements. Science.

[237] Han BY, Liu Z, Hu X, Ling H (2022). HNRNPU promotes the progression of triple-negative breast cancer via RNA transcription and alternative splicing mechanisms. Cell Death Dis..

[238] Zhou L (2006). Monocyte chemoattractant protein-1 induces a novel transcription factor that causes cardiac myocyte apoptosis and ventricular dysfunction. Circ. Res..

[239] Chen F (2021). MCPIP1-mediated NFIC alternative splicing inhibits proliferation of triple-negative breast cancer via cyclin D1-Rb-E2F1 axis. Cell Death Dis..

[240] Yang J (2015). LIN28A modulates splicing and gene expression programs in breast cancer cells. Mol. Cell Biol..

[241] Kim SJ (2020). RNA-binding protein NONO contributes to cancer cell growth and confers drug resistance as a theranostic target in TNBC. Theranostics.

[242] Iino K (2020). RNA-binding protein NONO promotes breast cancer proliferation by post-transcriptional regulation of SKP2 and E2F8. Cancer Sci..

[243] Naro C (2021). The oncogenic kinase NEK2 regulates an RBFOX2-dependent pro-mesenchymal splicing program in triple-negative breast cancer cells. J. Exp. Clin. Cancer Res..

[244] Shen DW, Pouliot LM, Hall MD, Gottesman MM (2012). Cisplatin resistance: a cellular self-defense mechanism resulting from multiple epigenetic and genetic changes. Pharm. Rev..

[245] Gokmen-Polar Y (2019). Splicing factor ESRP1 controls ER-positive breast cancer by altering metabolic pathways. EMBO Rep..

[246] Ali S, Coombes RC (2002). Endocrine-responsive breast cancer and strategies for combating resistance. Nat. Rev. Cancer.

[247] Xu Y (2021). ERalpha is an RNA-binding protein sustaining tumor cell survival and drug resistance. Cell.

[248] Elhasnaoui J (2021). The estrogen receptor alpha signaling pathway controls alternative splicing in the absence of ligands in breast cancer cells. Cancers.

[249] Wang C (2020). SRPK1 acetylation modulates alternative splicing to regulate cisplatin resistance in breast cancer cells. Commun. Biol..

[250] Choudhary C (2009). Lysine acetylation targets protein complexes and co-regulates major cellular functions. Science.

[251] Huang R (2020). The construction of bone metastasis-specific prognostic model and co-expressed network of alternative splicing in breast cancer. Front. Cell Dev. Biol..

[252] Coomer AO (2019). Alternative splicing in lung cancer. Biochim Biophys. Acta Gene Regul. Mech..

[253] Liu J (2022). Prognostic alternative splicing events related splicing factors define the tumor microenvironment and pharmacogenomic landscape in lung adenocarcinoma. Aging.

[254] Wu Q (2022). Multi-omics analysis reveals RNA splicing alterations and their biological and clinical implications in lung adenocarcinoma. Signal Transduct. Target Ther..

[255] de Miguel FJ (2016). A large-scale analysis of alternative splicing reveals a key role of QKI in lung cancer. Mol. Oncol..

[256] Cao Y (2021). RBM10 regulates tumor apoptosis, proliferation, and metastasis. Front. Oncol..

[257] Wang JZ (2021). QKI-5 regulates the alternative splicing of cytoskeletal gene ADD3 in lung cancer. J. Mol. Cell Biol..

[258] Zhu S (2021). SAM68 promotes tumorigenesis in lung adenocarcinoma by regulating metabolic conversion via PKM alternative splicing. Theranostics.

[259] Jamal-Hanjani M (2017). Tracking the evolution of non-small-cell lung cancer. New Engl. J. Med..

[260] Zhao J (2017). Functional analysis reveals that RBM10 mutations contribute to lung adenocarcinoma pathogenesis by deregulating splicing. Sci. Rep..

[261] Lv Y (2021). SRSF1 inhibits autophagy through regulating Bcl-x splicing and interacting with PIK3C3 in lung cancer. Signal Transduct. Target Ther..

[262] Nanjo S (2022). Deficiency of the splicing factor RBM10 limits EGFR inhibitor response in EGFR-mutant lung cancer. J. Clin. Investig..

[263] Zhang S (2020). RNA binding motif protein 10 suppresses lung cancer progression by controlling alternative splicing of eukaryotic translation initiation factor 4H. EBioMedicine.

[264] Truitt ML, Ruggero D (2017). New frontiers in translational control of the cancer genome (vol 16, pg 288, 2016). Nat. Rev. Cancer.

[265] Li S (2022). Nuclear Aurora kinase A switches m(6)A reader YTHDC1 to enhance an oncogenic RNA splicing of tumor suppressor RBM4. Signal Transduct. Target Ther..

[266] Bray F (2018). Global cancer statistics 2018: GLOBOCAN estimates of incidence and mortality worldwide for 36 cancers in 185 countries. CA Cancer J. Clin..

[267] Charalampakis N (2018). Medical management of gastric cancer: a 2017 update. Cancer Med..

[268] Feng H (2020). Identification and validation of critical alternative splicing events and splicing factors in gastric cancer progression. J. Cell Mol. Med..

[269] Jun Y (2022). Comprehensive analysis of alternative splicing in gastric cancer identifies epithelial-mesenchymal transition subtypes associated with survival. Cancer Res..

[270] Zhang F (2021). LncRNA CRNDE attenuates chemoresistance in gastric cancer via SRSF6-regulated alternative splicing of PICALM. Mol. Cancer.

[271] Di Matteo A (2023). Alternative splicing changes promoted by NOVA2 upregulation in endothelial cells and relevance for gastric cancer. Int. J. Mol. Sci..

[272] Siegel RL, Miller KD, Jemal A (2017). Cancer statistics, 2017. CA Cancer J. Clin..

[273] Lee J (2022). ESRP1-regulated isoform switching of LRRFIP2 determines metastasis of gastric cancer. Nat. Commun..

[274] He Q (2022). LINC00924-induced fatty acid metabolic reprogramming facilitates gastric cancer peritoneal metastasis via hnRNPC-regulated alternative splicing of Mnk2. Cell Death Dis..

[275] Liang X (2018). PTBP3 contributes to the metastasis of gastric cancer by mediating CAV1 alternative splicing. Cell Death Dis..

[276] Wang X (2021). CircURI1 interacts with hnRNPM to inhibit metastasis by modulating alternative splicing in gastric cancer. Proc. Natl. Acad. Sci. USA.

[277] Lee SCW (2016). Modulation of splicing catalysis for therapeutic targeting of leukemia with mutations in genes encoding spliceosomal proteins (vol 22, pg 672, 2016). Nat. Med..

[278] Yoshimi A, Abdel-Wahab O (2017). Molecular pathways: understanding and targeting mutant spliceosomal proteins. Clin. Cancer Res..

[279] Yoshimi A (2019). Coordinated alterations in RNA splicing and epigenetic regulation drive leukaemogenesis. Nature.

[280] Inoue D (2019). Spliceosomal disruption of the non-canonical BAF complex in cancer. Nature.

[281] Lee SC (2018). Synthetic lethal and convergent biological effects of cancer-associated spliceosomal gene mutations. Cancer Cell.

[282] Tran TM, Rao DS (2022). RNA binding proteins in MLL-rearranged leukemia. Exp. Hematol. Oncol..

[283] Hodson DJ, Screen M, Turner M (2019). RNA-binding proteins in hematopoiesis and hematological malignancy. Blood.

[284] De Kouchkovsky I, Abdul-Hay M (2016). ‘Acute myeloid leukemia: a comprehensive review and 2016 update’. Blood Cancer J..

[285] Al-Harbi S (2020). An update on the molecular pathogenesis and potential therapeutic targeting of AML with t(8;21)(q22;q22.1);RUNX1-RUNX1T1. Blood Adv..

[286] Mandoli A (2016). The hematopoietic transcription factors RUNX1 and ERG prevent AML1-ETO oncogene overexpression and onset of the apoptosis program in t(8;21) AMLs. Cell Rep..

[287] Trombly DJ (2015). Genome-wide co-occupancy of AML1-ETO and N-CoR defines the t(8;21) AML signature in leukemic cells. BMC Genomics.

[288] Grinev VV (2021). RUNX1/RUNX1T1 mediates alternative splicing and reorganises the transcriptional landscape in leukemia. Nat. Commun..

[289] Rivera OD (2021). Alternative splicing redefines landscape of commonly mutated genes in acute myeloid leukemia. Proc. Natl. Acad. Sci. USA.

[290] Radzisheuskaya A (2019). PRMT5 methylome profiling uncovers a direct link to splicing regulation in acute myeloid leukemia. Nat. Struct. Mol. Biol..

[291] Wang E (2019). Targeting an RNA-binding protein network in acute myeloid leukemia. Cancer Cell.

[292] Waldman T (2020). Emerging themes in cohesin cancer biology. Nat. Rev. Cancer.

[293] Singh AK (2023). Cohesin regulates alternative splicing. Sci. Adv..

[294] Yang YT (2023). Evaluation of the clinical significance of global mRNA alternative splicing in patients with acute myeloid leukemia. Am. J. Hematol..

[295] Wang E (2023). Modulation of RNA splicing enhances response to BCL2 inhibition in leukemia. Cancer Cell.

[296] Wei Y (2013). Toll-like receptor alterations in myelodysplastic syndrome. Leukemia.

[297] Zhao HG, Deininger M (2023). Always stressed but never exhausted: how stem cells in myeloid neoplasms avoid extinction in inflammatory conditions. Blood.

[298] Florian MC (2012). Cdc42 activity regulates hematopoietic stem cell aging and rejuvenation. Cell Stem Cell.

[299] Fang J (2017). Ubiquitination of hnRNPA1 by TRAF6 links chronic innate immune signaling with myelodysplasia. Nat. Immunol..

[300] Zhou Y (2020). Posttranslational regulation of the exon skipping machinery controls aberrant splicing in leukemia. Cancer Discov..

[301] Schmitz R (2012). Burkitt lymphoma pathogenesis and therapeutic targets from structural and functional genomics. Nature.

[302] Yamazaki T, Liu LZ, Conlon EG, Manley JL (2020). Burkitt lymphoma-related TCF3 mutations alter TCF3 alternative splicing by disrupting hnRNPH1 binding. Rna Biol..

[303] Orlando EJ (2018). Genetic mechanisms of target antigen loss in CAR19 therapy of acute lymphoblastic leukemia. Nat. Med..

[304] Roberts KG (2018). Genetics and prognosis of ALL in children vs adults. Hematol.-Am. Soc. Hematol..

[305] Zhao YQ (2021). Tumor-intrinsic and -extrinsic determinants of response to blinatumomab in adults with B-ALL. Blood.

[306] Rabilloud T (2021). Single-cell profiling identifies pre-existing CD19-negative subclones in a B-ALL patient with CD19-negative relapse after CAR-T therapy. Nat. Commun..

[307] Bagashev A (2018). CD19 alterations emerging after CD19-directed immunotherapy cause retention of the misfolded protein in the endoplasmic reticulum. Mol. Cell Biol..

[308] Sotillo E (2015). Convergence of acquired mutations and alternative splicing of CD19 enables resistance to CART-19 immunotherapy. Cancer Discov..

[309] Asnani M (2020). Retention of CD19 intron 2 contributes to CART-19 resistance in leukemias with subclonal frameshift mutations in CD19. Leukemia.

[310] Cortes-Lopez M (2022). High-throughput mutagenesis identifies mutations and RNA-binding proteins controlling CD19 splicing and CART-19 therapy resistance. Nat. Commun..

[311] Shinohara H (2016). Perturbation of energy metabolism by fatty-acid derivative AIC-47 and imatinib in BCR-ABL-harboring leukemic cells. Cancer Lett..

[312] Chang AJ, Autio KA, Roach M, Scher HI (2014). High-risk prostate cancer-classification and therapy. Nat. Rev. Clin. Oncol..

[313] Munkley J, Livermore K, Rajan P, Elliott DJ (2017). RNA splicing and splicing regulator changes in prostate cancer pathology. Hum. Genet..

[314] Marima R (2021). MicroRNA and alternative mRNA splicing events in cancer drug response/resistance: potent therapeutic targets. Biomedicines.

[315] Wach S, Taubert H, Cronauer M (2020). Role of androgen receptor splice variants, their clinical relevance and treatment options. World J. Urol..

[316] Rebello RJ (2021). Prostate cancer. Nat. Rev. Dis. Prim..

[317] Del Giudice M (2022). FOXA1 regulates alternative splicing in prostate cancer. Cell Rep..

[318] Urbanski L (2022). MYC regulates a pan-cancer network of co-expressed oncogenic splicing factors. Cell Rep..

[319] Koh CM (2015). MYC regulates the core pre-mRNA splicing machinery as an essential step in lymphomagenesis. Nature.

[320] Phillips JW (2020). Pathway-guided analysis identifies Myc-dependent alternative pre-mRNA splicing in aggressive prostate cancers. Proc. Natl. Acad. Sci. USA.

[321] Chen X (2023). The RNA-binding proteins hnRNP H and F regulate splicing of a MYC-dependent HRAS exon in prostate cancer cells. Proc. Natl. Acad. Sci. USA.

[322] Li WJ (2021). Profiling PRMT methylome reveals roles of hnRNPA1 arginine methylation in RNA splicing and cell growth. Nat. Commun..

[323] Labrecque MP (2021). RNA splicing factors SRRM3 and SRRM4 distinguish molecular phenotypes of castration-resistant neuroendocrine prostate cancer. Cancer Res..

[324] Zhang W (2021). The bone microenvironment invigorates metastatic seeds for further dissemination. Cell.

[325] Zeng Y (2013). Stress-response protein RBM3 attenuates the stem-like properties of prostate cancer cells by interfering with CD44 variant splicing. Cancer Res..

[326] Zhang S (2023). RBM3 suppresses stemness remodeling of prostate cancer in bone microenvironment by modulating N6-methyladenosine on CTNNB1 mRNA. Cell Death Dis..

[327] Tripathi V (2016). Direct regulation of alternative splicing by SMAD3 through PCBP1 is essential to the tumor-promoting role of TGF-beta. Mol. Cell.

[328] Chen Q (2021). TGF-beta1 promotes epithelial-to-mesenchymal transition and stemness of prostate cancer cells by inducing PCBP1 degradation and alternative splicing of CD44. Cell Mol. Life Sci..

[329] Fabris L (2016). The potential of microRNAs as prostate cancer biomarkers. Eur. Urol..

[330] Paschalis A (2018). Alternative splicing in prostate cancer. Nat. Rev. Clin. Oncol..

[331] Liu LL (2014). Mechanisms of the androgen receptor splicing in prostate cancer cells. Oncogene.

[332] Lu Y (2023). Emerging pharmacotherapeutic strategies to overcome undruggable proteins in cancer. Int. J. Biol. Sci..

[333] Chou FJ (2020). Preclinical studies using cisplatin/carboplatin to restore the Enzalutamide sensitivity via degrading the androgen receptor splicing variant 7 (ARv7) to further suppress Enzalutamide resistant prostate cancer. Cell Death Dis..

[334] Xi Y, Xu P (2021). Global colorectal cancer burden in 2020 and projections to 2040. Transl. Oncol..

[335] Zhou L (2022). Hypoxia-induced lncRNA STEAP3-AS1 activates Wnt/beta-catenin signaling to promote colorectal cancer progression by preventing m(6)A-mediated degradation of STEAP3 mRNA. Mol. Cancer.

[336] Huan L (2020). Hypoxia induced LUCAT1/PTBP1 axis modulates cancer cell viability and chemotherapy response. Mol. Cancer.

[337] Mochizuki Y (2021). Alternative microexon splicing by RBFOX2 and PTBP1 is associated with metastasis in colorectal cancer. Int. J. Cancer.

[338] Liu W (2023). Comprehensive analysis of RNA-binding protein SRSF2-dependent alternative splicing signature in malignant proliferation of colorectal carcinoma. J. Biol. Chem..

[339] Wan L (2019). SRSF6-regulated alternative splicing that promotes tumour progression offers a therapy target for colorectal cancer. Gut.

[340] Han F (2022). GLTSCR1 coordinates alternative splicing and transcription elongation of ZO1 to regulate colorectal cancer progression. J. Mol. Cell Biol..

[341] Zhao S (2023). Exosomal transfer of miR-181b-5p confers senescence-mediated doxorubicin resistance via modulating BCLAF1 in breast cancer. Br. J. Cancer.

[342] Zhou X (2014). BCLAF1 and its splicing regulator SRSF10 regulate the tumorigenic potential of colon cancer cells. Nat. Commun..

[343] Pan YJ (2022). Alternative splicing of HSPA12A pre-RNA by SRSF11 contributes to metastasis potential of colorectal cancer. Clin. Transl. Med..

[344] Tam BY (2020). The CLK inhibitor SM08502 induces anti-tumor activity and reduces Wnt pathway gene expression in gastrointestinal cancer models. Cancer Lett..

[345] Hellborg F (2001). Human wig-1, a p53 target gene that encodes a growth inhibitory zinc finger protein. Oncogene.

[346] Fearon ER (2011). Molecular genetics of colorectal cancer. Annu. Rev. Pathol..

[347] Janic A (2018). DNA repair processes are critical mediators of p53-dependent tumor suppression. Nat. Med..

[348] Muys BR (2021). The p53-induced RNA-binding protein ZMAT3 is a splicing regulator that inhibits the splicing of oncogenic CD44 variants in colorectal carcinoma. Genes Dev..

[349] Muys BR (2023). Matrin3 regulates mitotic spindle dynamics by controlling alternative splicing of CDC14B. Cell Rep..

[350] Ueda J (2014). Epithelial splicing regulatory protein 1 is a favorable prognostic factor in pancreatic cancer that attenuates pancreatic metastases. Oncogene.

[351] Cheng DK (2021). Oncogenic KRAS engages an RSK1/NF1 pathway to inhibit wild-type RAS signaling in pancreatic cancer. Proc. Natl. Acad. Sci. USA.

[352] Baer JM (2023). Fibrosis induced by resident macrophages has divergent roles in pancreas inflammatory injury and PDAC. Nat. Immunol..

[353] Wan L (2023). Splicing factor SRSF1 promotes pancreatitis and KRASG12D-mediated pancreatic cancer. Cancer Discov..

[354] Mo Y (2022). Circular RNA circPVT1 promotes nasopharyngeal carcinoma metastasis via the beta-TrCP/c-Myc/SRSF1 positive feedback loop. Mol. Cancer.

[355] Biffi G (2019). IL1-induced JAK/STAT signaling is antagonized by TGFbeta to shape CAF heterogeneity in pancreatic ductal adenocarcinoma. Cancer Discov..

[356] Jones S (2008). Core signaling pathways in human pancreatic cancers revealed by global genomic analyses. Science.

[357] Jbara A (2023). RBFOX2 modulates a metastatic signature of alternative splicing in pancreatic cancer. Nature.

[358] Huang XT (2021). HNRNPC impedes m(6)A-dependent anti-metastatic alternative splicing events in pancreatic ductal adenocarcinoma. Cancer Lett..

[359] Yang G (2021). Integrative genomic analysis of gemcitabine resistance in pancreatic cancer by patient-derived xenograft models. Clin. Cancer Res..

[360] Calabretta S (2016). Modulation of PKM alternative splicing by PTBP1 promotes gemcitabine resistance in pancreatic cancer cells. Oncogene.

[361] Wang P (2019). Targeted regulation of Rell2 by microRNA-18a is implicated in the anti-metastatic effect of polyphyllin VI in breast cancer cells. Eur. J. Pharm..

[362] Li Z (2023). DHX38 restricts chemoresistance by regulating the alternative pre-mRNA splicing of RELL2 in pancreatic ductal adenocarcinoma. PLoS Genet..

[363] Tian JB (2022). Aberrant RNA splicing is a primary link between genetic variation and pancreatic cancer risk. Cancer Res..

[364] Xia CF (2022). Cancer statistics in China and United States, 2022: profiles, trends, and determinants. Chin. Med. J..

[365] Li X (2021). The immunological and metabolic landscape in primary and metastatic liver cancer. Nat. Rev. Cancer.

[366] Huang S (2021). PCBP1 regulates the transcription and alternative splicing of metastasis‑related genes and pathways in hepatocellular carcinoma. Sci. Rep..

[367] Zhang Y, Qian J, Gu C, Yang Y (2021). Alternative splicing and cancer: a systematic review. Signal Transduct. Target Ther..

[368] Caruso S, Nault JC (2019). A dive into the deep heterogeneity of hepatocellular carcinoma. Gastroenterology.

[369] Lee SE, Alcedo KP, Kim HJ, Snider NT (2020). Alternative splicing in hepatocellular carcinoma. Cell Mol. Gastroenterol. Hepatol..

[370] Chen H (2019). Long-read RNA sequencing identifies alternative splice variants in hepatocellular carcinoma and tumor-specific isoforms. Hepatology.

[371] Luo C (2017). SRSF2 regulates alternative splicing to drive hepatocellular carcinoma development. Cancer Res..

[372] Liu L (2023). HBV enhances sorafenib resistance in hepatocellular carcinoma by reducing ferroptosis via SRSF2-mediated abnormal PCLAF splicing. Int. J. Mol. Sci..

[373] Chang C (2022). The aberrant upregulation of exon 10-inclusive SREK1 through SRSF10 acts as an oncogenic driver in human hepatocellular carcinoma. Nat. Commun..

[374] Sen S, Langiewicz M, Jumaa H, Webster NJ (2015). Deletion of serine/arginine-rich splicing factor 3 in hepatocytes predisposes to hepatocellular carcinoma in mice. Hepatology.

[375] Chen D (2021). PPM1G promotes the progression of hepatocellular carcinoma via phosphorylation regulation of alternative splicing protein SRSF3. Cell Death Dis..

[376] Wang H (2019). A coiled-coil domain containing 50 splice variant is modulated by serine/arginine-rich splicing factor 3 and promotes hepatocellular carcinoma in mice by the Ras signaling pathway. Hepatology.

[377] Liu X (2022). SRSF10 stabilizes CDC25A by triggering exon 6 skipping to promote hepatocarcinogenesis. J. Exp. Clin. Cancer Res..

[378] Shilo A (2014). Splicing factor hnRNP A2 activates the Ras-MAPK-ERK pathway by controlling A-Raf splicing in hepatocellular carcinoma development. RNA.

[379] Zhou ZJ (2014). HNRNPAB induces epithelial-mesenchymal transition and promotes metastasis of hepatocellular carcinoma by transcriptionally activating SNAIL. Cancer Res..

[380] Wang H (2018). Long noncoding RNA miR503HG, a prognostic indicator, inhibits tumor metastasis by regulating the HNRNPA2B1/NF-kappaB pathway in hepatocellular carcinoma. Theranostics.

[381] Shen L (2020). Skipping of exon 10 in Axl pre-mRNA regulated by PTBP1 mediates invasion and metastasis process of liver cancer cells. Theranostics.

[382] Yu L (2020). MTR4 drives liver tumorigenesis by promoting cancer metabolic switch through alternative splicing. Nat. Commun..

[383] Liu D (2022). HNRNPC downregulation inhibits IL-6/STAT3-mediated HCC metastasis by decreasing HIF1A expression. Cancer Sci..

[384] Zhu GQ (2022). Targeting HNRNPM inhibits cancer stemness and enhances antitumor immunity in Wnt-activated hepatocellular carcinoma. Cell Mol. Gastroenterol. Hepatol..

[385] Li SL (2019). Transcriptome-wide analysis reveals the landscape of aberrant alternative splicing events in liver cancer. Hepatology.

[386] Qiao Y (2022). RNA binding protein RALY activates the cholesterol synthesis pathway through an MTA1 splicing switch in hepatocellular carcinoma. Cancer Lett..

[387] Sohail M, Xie JY (2015). Evolutionary emergence of a novel splice variant with an opposite effect on the cell cycle. Mol. Cell Biol..

[388] Wen C (2022). SRSF3 and HNRNPH1 regulate radiation-induced alternative splicing of protein arginine methyltransferase 5 in hepatocellular carcinoma. Int. J. Mol. Sci..

[389] Hu Z (2020). Splicing regulator p54(nrb)/non-POU domain-containing octamer-binding protein enhances carcinogenesis through oncogenic isoform switch of MYC Box-dependent interacting protein 1 in hepatocellular carcinoma. Hepatology.

[390] Kahles A (2018). Comprehensive analysis of alternative splicing across tumors from 8,705 patients. Cancer Cell.

[391] Ajiro M (2016). A genome landscape of SRSF3-regulated splicing events and gene expression in human osteosarcoma U2OS cells. Nucleic Acids Res..

[392] Jia R (2019). Oncogenic splicing factor SRSF3 regulates ILF3 alternative splicing to promote cancer cell proliferation and transformation. RNA.

[393] Zhang S (2016). MYCN controls an alternative RNA splicing program in high-risk metastatic neuroblastoma. Cancer Lett..

[394] Slansky JE, Spellman PT (2019). Alternative splicing in tumors—a path to immunogenicity?. New Engl. J. Med..

[395] Bonnal SC, Lopez-Oreja I, Valcarcel J (2020). Roles and mechanisms of alternative splicing in cancer - implications for care. Nat. Rev. Clin. Oncol..

[396] Desterro J, Bak-Gordon P, Carmo-Fonseca M (2020). Targeting mRNA processing as an anticancer strategy. Nat. Rev. Drug Discov..

[397] Kalev P (2021). MAT2A inhibition blocks the growth of MTAP-deleted cancer cells by reducing PRMT5-dependent mRNA splicing and inducing DNA damage. Cancer Cell.

[398] Poulard C (2023). Nuclear PRMT5 is a biomarker of sensitivity to tamoxifen in ERalpha(+) breast cancer. EMBO Mol. Med..

[399] Banasavadi-Siddegowda YK (2018). PRMT5 as a druggable target for glioblastoma therapy. Neuro Oncol..

[400] Braun CJ (2017). Coordinated Splicing Of Regulatory Detained Introns Within Oncogenic Transcripts Creates An Exploitable Vulnerability In Malignant Glioma. Cancer Cell.

[401] Seufert L, Benzing T, Ignarski M, Muller RU (2022). RNA-binding proteins and their role in kidney disease. Nat. Rev. Nephrol..

[402] Low YH, Asi Y, Foti SC, Lashley T (2021). Heterogeneous nuclear ribonucleoproteins: implications in neurological diseases. Mol. Neurobiol..

[403] Krach F (2022). Aberrant NOVA1 function disrupts alternative splicing in early stages of amyotrophic lateral sclerosis. Acta Neuropathol..

[404] Neumann M (2009). Phosphorylation of S409/410 of TDP-43 is a consistent feature in all sporadic and familial forms of TDP-43 proteinopathies. Acta Neuropathol..

[405] Bjork RT, Mortimore NP, Loganathan S, Zarnescu DC (2022). Dysregulation of translation in TDP-43 proteinopathies: deficits in the RNA supply chain and local protein production. Front. Neurosci..

[406] MacNair L (2016). MTHFSD and DDX58 are novel RNA-binding proteins abnormally regulated in amyotrophic lateral sclerosis. Brain.

[407] Martinez FJ (2016). Protein-RNA networks regulated by normal and ALS-associated mutant HNRNPA2B1 in the nervous system. Neuron.

[408] Scheltens P (2021). Alzheimer’s disease. Lancet.

[409] Gu J (2017). Transactive response DNA-binding protein 43 (TDP-43) regulates alternative splicing of tau exon 10: Implications for the pathogenesis of tauopathies. J. Biol. Chem..

[410] van Bergeijk P (2019). SRSF1 and PTBP1 are trans-acting factors that suppress the formation of a CD33 splicing isoform linked to Alzheimer’s disease risk. Mol. Cell Biol..

[411] Zhang Q (2021). Nuclear speckle specific hnRNP D-like prevents age- and AD-related cognitive decline by modulating RNA splicing. Mol. Neurodegener..

[412] Bhatnagar A (2023). Tip60’s novel RNA-Binding function modulates alternative splicing of pre-mRNA targets implicated in Alzheimer’s disease. J. Neurosci..

[413] Tabrizi SJ (2022). Potential disease-modifying therapies for Huntington’s disease: lessons learned and future opportunities. Lancet Neurol..

[414] Elorza A (2021). Huntington’s disease-specific mis-splicing unveils key effector genes and altered splicing factors. Brain.

[415] Li D, Yu W, Lai M (2023). Towards understandings of serine/arginine-rich splicing factors. Acta Pharm. Sin. B.

[416] Ibanez-Costa A (2022). Splicing machinery is impaired in rheumatoid arthritis, associated with disease activity and modulated by anti-TNF therapy. Ann. Rheum. Dis..

[417] Ren P (2021). Alternative splicing: a new cause and potential therapeutic target in autoimmune disease. Front. Immunol..

[418] Wang J (2012). SpliceDisease database: linking RNA splicing and disease. Nucleic Acids Res..

[419] Monteuuis G (2019). The changing paradigm of intron retention: regulation, ramifications and recipes. Nucleic Acids Res..

[420] Ingelfinger JR, Jarcho JA (2017). Increase in the incidence of diabetes and its implications. New Engl. J. Med..

[421] Zheng Y, Ley SH, Hu FB (2018). Global aetiology and epidemiology of type 2 diabetes mellitus and its complications. Nat. Rev. Endocrinol..

[422] Chen X (2022). Advances in the study of RNA-binding proteins in diabetic complications. Mol. Metab..

[423] Nutter CA, Kuyumcu-Martinez MN (2018). Emerging roles of RNA-binding proteins in diabetes and their therapeutic potential in diabetic complications. Wiley Interdiscip. Rev. RNA.

[424] Alvelos MI (2018). When one becomes many-Alternative splicing in beta-cell function and failure. Diabetes Obes. Metab..

[425] Ravanidis S, Kattan FG, Doxakis E (2018). Unraveling the pathways to neuronal homeostasis and disease: mechanistic insights into the role of RNA-binding proteins and associated factors. Int. J. Mol. Sci..

[426] Marcheva B (2020). A role for alternative splicing in circadian control of exocytosis and glucose homeostasis. Genes Dev..

[427] McCartney CR, Marshall JC (2016). CLINICAL PRACTICE. Polycystic ovary syndrome. New Engl. J. Med..

[428] Wang F (2015). Alternative splicing of the androgen receptor in polycystic ovary syndrome. Proc. Natl. Acad. Sci. USA.

[429] Younossi Z (2019). Global perspectives on nonalcoholic fatty liver disease and nonalcoholic steatohepatitis. Hepatology.

[430] Del Rio-Moreno M (2019). Dysregulation of the splicing machinery is associated to the development of nonalcoholic fatty liver disease. J. Clin. Endocrinol. Metab..

[431] Kumar D (2019). Degradation of splicing factor SRSF3 contributes to progressive liver disease. J. Clin. Investig..

[432] Zhang Z (2020). Hepatic HuR modulates lipid homeostasis in response to high-fat diet. Nat. Commun..

[433] Li Y (2021). DRAK2 aggravates nonalcoholic fatty liver disease progression through SRSF6-associated RNA alternative splicing. Cell Metab..

[434] Hyun J (2021). Dysregulation of the ESRP2-NF2-YAP/TAZ axis promotes hepatobiliary carcinogenesis in non-alcoholic fatty liver disease. J. Hepatol..

[435] Soussan P (2008). Expression of defective hepatitis B virus particles derived from singly spliced RNA is related to liver disease. J. Infect. Dis..

[436] Duriez M (2017). Alternative splicing of hepatitis B virus: a novel virus/host interaction altering liver immunity. J. Hepatol..

[437] de Bruin RG, Rabelink TJ, van Zonneveld AJ, van der Veer EP (2017). Emerging roles for RNA-binding proteins as effectors and regulators of cardiovascular disease. Eur. Heart J..

[438] Liu J (2019). RNA binding protein 24 deletion disrupts global alternative splicing and causes dilated cardiomyopathy. Protein Cell.

[439] Gan P (2022). RBPMS is an RNA-binding protein that mediates cardiomyocyte binucleation and cardiovascular development. Dev. Cell.

[440] Paronetto MP, Passacantilli I, Sette C (2016). Alternative splicing and cell survival: from tissue homeostasis to disease. Cell Death Differ..

[441] Agosto LM, Lynch KW (2018). Alternative pre-mRNA splicing switch controls hESC pluripotency and differentiation. Genes Dev..

[442] Yuan S, Norgard RJ, Stanger BZ (2019). Cellular plasticity in cancer. Cancer Discov..

[443] Leclair NK (2020). Poison exon splicing regulates a coordinated network of SR protein expression during differentiation and tumorigenesis. Mol. Cell.

[444] Muller-McNicoll M, Rossbach O, Hui J, Medenbach J (2019). Auto-regulatory feedback by RNA-binding proteins. J. Mol. Cell Biol..

[445] Zhao Z (2023). QKI shuttles internal m(7)G-modified transcripts into stress granules and modulates mRNA metabolism. Cell.

[446] Simmler P (2023). Mutant SF3B1 promotes malignancy in PDAC. eLife.

[447] Zhang J (2019). Disease-causing mutations in SF3B1 alter splicing by disrupting interaction with SUGP1. Mol. Cell.

[448] Shuai S (2019). The U1 spliceosomal RNA is recurrently mutated in multiple cancers. Nature.

[449] Bowling EA (2021). Spliceosome-targeted therapies trigger an antiviral immune response in triple-negative breast cancer. Cell.

[450] Ziegenhain C (2017). Comparative analysis of single-cell RNA sequencing methods. Mol. Cell.

[451] Ardui S, Ameur A, Vermeesch JR, Hestand MS (2018). Single molecule real-time (SMRT) sequencing comes of age: applications and utilities for medical diagnostics. Nucleic Acids Res..

[452] Liao L (2023). Lysine 2-hydroxyisobutyrylation of NAT10 promotes cancer metastasis in an ac4C-dependent manner. Cell Res..

[453] Luo QJ (2021). RNA structure probing reveals the structural basis of Dicer binding and cleavage. Nat. Commun..

[454] Wan Y, Qu K, Ouyang Z, Chang HY (2013). Genome-wide mapping of RNA structure using nuclease digestion and high-throughput sequencing. Nat. Protoc..

[455] Mehta M, Raguraman R, Ramesh R, Munshi A (2022). RNA binding proteins (RBPs) and their role in DNA damage and radiation response in cancer. Adv. Drug Deliv. Rev..

[456] Gu X (2023). The midnolin-proteasome pathway catches proteins for ubiquitination-independent degradation. Science.

[457] Rossi G, Manfrin A, Lutolf MP (2018). Progress and potential in organoid research. Nat. Rev. Genet..

[458] Fang E (2022). Advances in COVID-19 mRNA vaccine development. Signal Transduct. Target Ther..

[459] Wang Y (2021). mRNA vaccine: a potential therapeutic strategy. Mol. Cancer.

[460] Warzecha CC (2009). ESRP1 and ESRP2 are epithelial cell-type-specific regulators of FGFR2 splicing. Mol. Cell.

[461] Burguera D (2017). Evolutionary recruitment of flexible Esrp-dependent splicing programs into diverse embryonic morphogenetic processes. Nat. Commun..

[462] Derham JM, Kalsotra A (2023). The discovery, function, and regulation of epithelial splicing regulatory proteins (ESRP) 1 and 2. Biochem. Soc. Trans..

[463] Pai TP (2013). Drosophila ORB protein in two mushroom body output neurons is necessary for long-term memory formation. Proc. Natl. Acad. Sci. USA.

[464] Lantz V, Ambrosio L, Schedl P (1992). The Drosophila orb gene is predicted to encode sex-specific germline RNA-binding proteins and has localized transcripts in ovaries and early embryos. Development.

[465] Kozlov E (2021). The role of CPEB family proteins in the nervous system function in the norm and pathology. Cell Biosci..

[466] Huang YS, Kan MC, Lin CL, Richter JD (2006). CPEB3 and CPEB4 in neurons: analysis of RNA-binding specificity and translational control of AMPA receptor GluR2 mRNA. EMBO J..

[467] Fernandez-Miranda G, Mendez R (2012). The CPEB-family of proteins, translational control in senescence and cancer. Ageing Res. Rev..

[468] Lachiondo-Ortega S (2022). Hu antigen R (HuR) protein structure, function and regulation in hepatobiliary tumors. Cancers.

[469] Majumder M (2022). HuR as a molecular target for cancer therapeutics and immune-related disorders. Adv. Drug Deliv. Rev..

[470] Geuens T, Bouhy D, Timmerman V (2016). The hnRNP family: insights into their role in health and disease. Hum. Genet..

[471] Yisraeli JK (2005). VICKZ proteins: a multi-talented family of regulatory RNA-binding proteins. Biol. Cell.

[472] Zhu TY, Hong LL, Ling ZQ (2023). Oncofetal protein IGF2BPs in human cancer: functions, mechanisms and therapeutic potential. Biomark. Res..

[473] Ramesh-Kumar D, Guil S (2022). The IGF2BP family of RNA binding proteins links epitranscriptomics to cancer. Semin. Cancer Biol..

[474] Gheldof A (2012). Evolutionary functional analysis and molecular regulation of the ZEB transcription factors. Cell Mol. Life Sci..

[475] Verstappen G (2008). Atypical Mowat-Wilson patient confirms the importance of the novel association between ZFHX1B/SIP1 and NuRD corepressor complex. Hum. Mol. Genet..

[476] Scott CL, Omilusik KD (2019). ZEBs: novel players in immune cell development and function. Trends Immunol..

[477] Li H (2021). Zinc finger E-box binding homeobox 1 and atherosclerosis: new insights and therapeutic potential. J. Cell Physiol..

[478] Briata P (2016). Diverse roles of the nucleic acid-binding protein KHSRP in cell differentiation and disease. Wiley Interdiscip. Rev. RNA.

[479] Patel P (2022). Intra-axonal translation of Khsrp mRNA slows axon regeneration by destabilizing localized mRNAs. Nucleic Acids Res..

[480] Stavraka C, Blagden S (2015). The La-related proteins, a family with connections to cancer. Biomolecules.

[481] Lizarrondo J, Dock-Bregeon AC, Martino L, Conte MR (2021). Structural dynamics in the La-module of La-related proteins. Rna Biol..

[482] Maraia RJ, Mattijssen S, Cruz-Gallardo I, Conte MR (2017). The La and related RNA-binding proteins (LARPs): structures, functions, and evolving perspectives. Wiley Interdiscip. Rev. RNA.

[483] Shyh-Chang N, Daley GQ (2013). Lin28: primal regulator of growth and metabolism in stem cells. Cell Stem Cell.

[484] Kudinov AE, Karanicolas J, Golemis EA, Boumber Y (2017). Musashi RNA-binding proteins as cancer drivers and novel therapeutic targets. Clin. Cancer Res..

[485] Smialek MJ, Ilaslan E, Sajek MP, Jaruzelska J (2021). Role of PUM RNA-binding proteins in cancer. Cancers.

[486] Li Z (2021). The RNA-binding motif protein family in cancer: friend or foe?. Front. Oncol..

[487] Sutherland LC, Rintala-Maki ND, White RD, Morin CD (2005). RNA binding motif (RBM) proteins: a novel family of apoptosis modulators?. J. Cell Biochem..

[488] Sanchez-Jimenez F, Sanchez-Margalet V (2013). Role of Sam68 in post-transcriptional gene regulation. Int. J. Mol. Sci..

[489] Fernandez-Gomez A, Izquierdo JM (2022). The multifunctional faces of T-cell intracellular antigen 1 in health and disease. Int. J. Mol. Sci..

[490] Velasco BR, Izquierdo JM (2022). T-cell intracellular antigen 1-like protein in physiology and pathology. Int. J. Mol. Sci..

[491] Guo AX, Cui JJ, Wang LY, Yin JY (2020). The role of CSDE1 in translational reprogramming and human diseases. Cell Commun. Signal.

[492] Carabet LA (2019). Computer-aided discovery of small molecules targeting the RNA splicing activity of hnRNP A1 in castration-resistant prostate cancer. Molecules.

[493] Supradit K (2022). Inhibition of serine/arginine-rich protein kinase-1 (SRPK1) prevents cholangiocarcinoma cells induced angiogenesis. Toxicol. Vitr..

[494] Han T (2017). Anticancer sulfonamides target splicing by inducing RBM39 degradation via recruitment to DCAF15. Science.

[495] Marcel V (2014). Modulation of p53beta and p53gamma expression by regulating the alternative splicing of TP53 gene modifies cellular response. Cell Death Differ..

[496] Sako Y (2017). Development of an orally available inhibitor of CLK1 for skipping a mutated dystrophin exon in Duchenne muscular dystrophy. Sci. Rep..

[497] Salvador F, Gomis RR (2018). CLK2 blockade modulates alternative splicing compromising MYC-driven breast tumors. EMBO Mol. Med..

[498] Chu WH (2023). Discovery of tetrahydroisoquinolineindole derivatives as first dual PRMT5 inhibitors/hnRNP E1 upregulators: design, synthesis and biological evaluation. Eur. J. Med. Chem..

[499] Datta A (2017). Manumycin A suppresses exosome biogenesis and secretion via targeted inhibition of Ras/Raf/ERK1/2 signaling and hnRNP H1 in castration-resistant prostate cancer cells. Cancer Lett..

[500] Benavides-Serrato A (2020). Repurposing potential of riluzole as an ITAF inhibitor in mTOR therapy resistant glioblastoma. Int. J. Mol. Sci..

[501] Meng N (2019). Novel role of heterogeneous nuclear ribonucleoprotein E1 in regulation of apoptosis and autophagy by a triazole derivative in vascular endothelial cells. Int. J. Biol. Sci..

[502] Shorrock HK, Gillingwater TH, Groen EJN (2018). Overview of current drugs and molecules in development for spinal muscular atrophy therapy. Drugs.

[503] Bondu S (2019). A variant erythroferrone disrupts iron homeostasis in SF3B1-mutated myelodysplastic syndrome. Sci. Transl. Med..

[504] Leivonen SK (2017). Alternative splicing discriminates molecular subtypes and has prognostic impact in diffuse large B-cell lymphoma. Blood Cancer J..

[505] Brander DM (2019). SET alpha and SET beta mRNA isoforms in chronic lymphocytic leukaemia. Br. J. Haematol..

[506] Moulton VR, Gillooly AR, Tsokos GC (2014). Ubiquitination regulates expression of the serine/arginine-rich splicing factor 1 (SRSF1) in normal and systemic lupus erythematosus (SLE) T cells. J. Biol. Chem..

[507] Kletzl H (2019). The oral splicing modifier RG7800 increases full length survival of motor neuron 2 mRNA and survival of motor neuron protein: results from trials in healthy adults and patients with spinal muscular atrophy. Neuromuscul. Disord..

[508] Seiler M (2018). H3B-8800, an orally available small-molecule splicing modulator, induces lethality in spliceosome-mutant cancers. Nat. Med..

[509] Krach F (2022). An alternative splicing modulator decreases mutant HTT and improves the molecular fingerprint in Huntington’s disease patient neurons. Nat. Commun..

[510] Ratni H (2016). Specific correction of alternative survival motor neuron 2 splicing by small molecules: discovery of a potential novel medicine to treat spinal muscular atrophy. J. Med. Chem..

[511] Yoda A (2021). CTX-712, a novel Clk inhibitor targeting myeloid neoplasms with SRSF2 mutation. Blood.

